# Template-based copying in chemically fuelled dynamic combinatorial libraries

**DOI:** 10.1038/s41557-024-01570-5

**Published:** 2024-07-16

**Authors:** Christine M. E. Kriebisch, Ludwig Burger, Oleksii Zozulia, Michele Stasi, Alexander Floroni, Dieter Braun, Ulrich Gerland, Job Boekhoven

**Affiliations:** 1 School of Natural Sciences, Department of Bioscience, Technical University of Munich, Garching, Germany; 2 Systems Biophysics Center for Nano-Science and Origins Cluster Initiative, Department of Physics, Ludwig-Maximilians-Universität München, Munich, Germany

**Keywords:** Origin of life, Dynamic combinatorial chemistry, Supramolecular polymers

## Abstract

One of science’s greatest challenges is determining how life can spontaneously emerge from a mixture of molecules. A complicating factor is that life and its molecules are inherently unstable—RNA and proteins are prone to hydrolysis and denaturation. For the de novo synthesis of life or to better understand its emergence at its origin, selection mechanisms are needed for unstable molecules. Here we present a chemically fuelled dynamic combinatorial library to model RNA oligomerization and deoligomerization and shine new light on selection and purification mechanisms under kinetic control. In the experiments, oligomers can only be sustained by continuous production. Hybridization is a powerful tool for selecting unstable molecules, offering feedback on oligomerization and deoligomerization rates. Moreover, we find that templation can be used to purify libraries of oligomers. In addition, template-assisted formation of oligomers within coacervate-based protocells changes its compartment’s physical properties, such as their ability to fuse. Such reciprocal coupling between oligomer production and physical properties is a key step towards synthetic life.

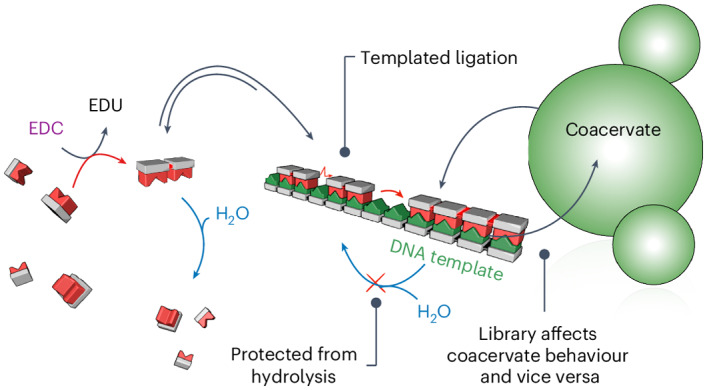

## Abstract (web summary)

Selection mechanisms were critical at the emergence of life and will also be important for the synthesis of life. Now, it has been shown that template-based copying controls the selection of unstable molecules in a chemically fuelled dynamic combinatorial library. Moreover, when encapsulated inside coacervate droplets, these mechanisms change the coacervate’s physical properties.

## Main

In dynamic combinatorial libraries, molecules react reversibly to form a mixture of interchanging library members^1–3^. A statistical distribution of products arises in which the thermodynamically most stable products are favoured. Self-assembly and hybridization further increase a product’s thermodynamic stability, thus increasing the likelihood of selection^3–8^. These mixtures of interchanging molecules are an appealing approach for studying mechanisms by which the molecules of life were selected from prebiotic molecular mixtures^1,4,9,10^. Dynamic combinatorial libraries have also been demonstrated to lead to Darwinian-like evolution of self-replicating molecules as the system evolves towards equilibrium^4,9–15^. However, life cannot exist in equilibrium. Life constantly transduces energy to escape equilibrium and uses that energy to propagate information and structure^16^. For life, selection rules and mechanisms that apply under kinetic control are relevant. Such mechanisms probably differ from those under thermodynamic control.

**Fig. 1:**
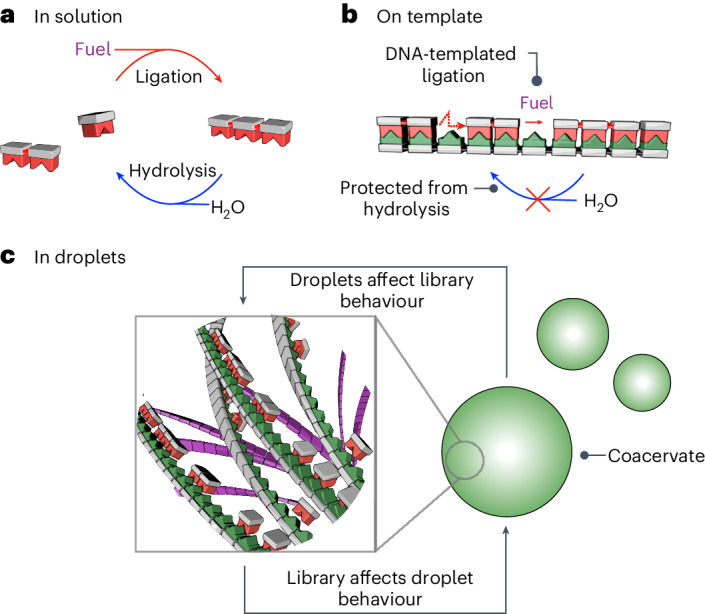
Template-based copying controls selection in chemically fuelled libraries, and when this happens inside a coacervate it alters the coacervate’s physical properties. **a**, Fuel oligomerizes monomers and spontaneous hydrolysis deoligomerizes the oligoanhydrides in solution. **b**, Interaction of monomers and oligoanhydrides with a template accelerates oligomerization by selectively concentrating longer oligomers. It simultaneously decelerates deoligomerization through a protection mechanism. **c**, Template-assisted formation of oligomers within coacervate-based protocells (green droplets) changes the coacervate’s physical properties, such as their ability to fuse. Throughout, the green pictograms represent the DNA or RNA template and the red pictograms depict the oligomers.

**Fig. 2:**
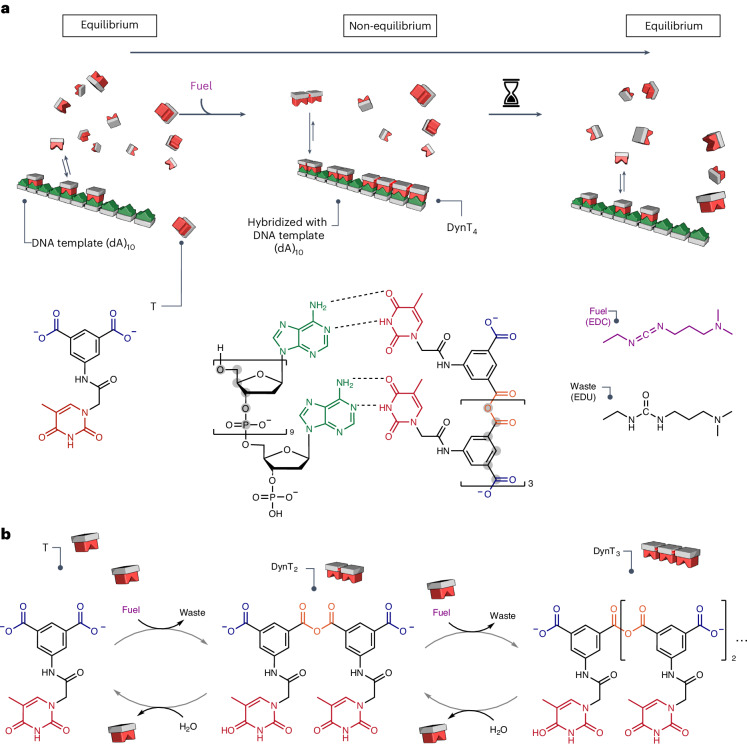
Description of the chemically fuelled library. **a**, Schematic representation of the concept of the chemically fuelled dynamic combinatorial library and the role of the template. Oligomers interact with the template. The six-atom repeating backbone unit of DNA and the oligomers (highlighted with grey shading) match. The molecular structures of the DNA template (dA)_10_, thymine-labelled monomer T and its oligomers, fuel 1-ethyl-3-(3-(dimethylamino)propyl)carbodiimide (EDC) and waste 1-ethyl-3-(3-(dimethylamino)propyl)urea (EDU) are shown. The reactive molecule sites, recognition motifs (for example, thymine) and complete molecular structures are colour-coded as follows: monomer acid (blue), thymine (red), adenine (green), oligomer anhydride (orange), fuel (purple) and waste (black). The pictograms for thymine oligomers are coloured red and the DNA template is coloured green. **b**, The chemical reaction system converts a chemical fuel (EDC) into waste (EDU) while building up oligomers that are part of a transient dynamic combinatorial library.

In this work we introduce templation in chemically fuelled dynamic combinatorial libraries to study how templates affect kinetic selection in dynamic combinatorial libraries (Fig. 1)^17–20^. Unlike conventional dynamic combinatorial libraries, chemically fuelled dynamic combinatorial libraries are in a non-equilibrium state where bonds form at the expense of high-energy molecules, which we call fuels. The newly formed bonds revert spontaneously through hydrolysis (Fig. 2a). The thermodynamically stable state is thus the initial pool of molecules, whereas mechanisms of kinetic selection dictate which products are most favoured. Fundamentally, this resembles RNA or DNA oligomerization, an energetically uphill process, whereas deoligomerization through hydrolysis is spontaneous. We found that hybridization is a powerful tool for kinetic selection: hybridization protects the oligomers from hydrolysis and favours their oligomerization. We refer to these mechanisms—protection from hydrolysis and favorization of oligomerization—as negative and positive feedback of the template on the oligomers, respectively. These feedback mechanisms can select molecules of specific lengths and sequences. Besides, we demonstrate that the library can change the physical properties of its environment. Thus, the compartment with its template influences the composition of the library, whereas the library influences the compartment (Fig. 1). Such reciprocal coupling between phenotype and genotype brings us closer to exploring Darwinian evolution in our systems in future work.

## Results and discussion

### Template-based selection in chemically fuelled libraries

**Extended Data Fig. 1:**
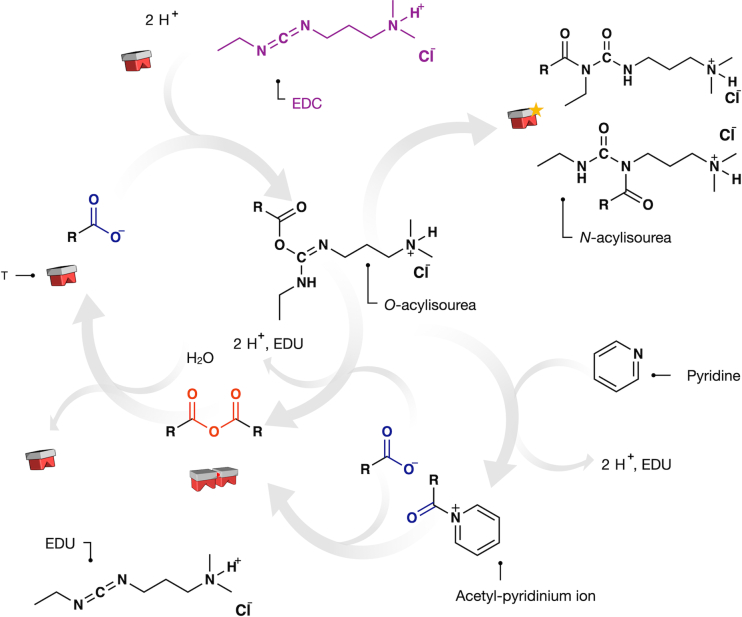
The chemical reaction cycle. Monomer **T** catalyzes the conversion of fuel EDC to waste EDU. Reactive molecule sites, or complete molecular structures, are color-coded as follows: monomer acid (blue), fuel (purple), waste (black), and oligomer anhydride (orange).

**Extended Data Fig. 2:**
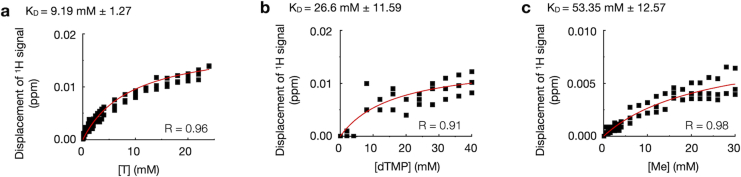
Determination of dissociation constant via ^1^H-NMR titration. The plot of the displacement of the chemical shift of adenine protons A1H8 of (dA)_10_ versus the concentration of **(a) T**, **(b)** dTMP or **(c) Me**. The curve is a fit, giving for **T** a K_D, 10_ of 9.19 mM ± 1.27 mM, for dTMP a K_D, 10_ of 26.6 mM ± 11.59 mM and for **Me** a K_D, 10_ of 53.55 mM ± 12.57 mM for the template, and thus a K_D, 1_ of 91.91 mM ± 12.7 mM, K_D, 1_ of 266 mM ± 115.9 mM and K_D, 1_ of 535.5 mM ± 125.7 mM per binding site, respectively. Source data

We used isophthalic acid as a monomer that can oligomerize at the expense of the condensing agent EDC^17^. These oligoanhydrides spontaneously deoligomerize through hydrolysis. We attached the nucleobase thymine to the isophthalic acid to form the thymine-labelled monomer T (Fig. 2a). Monomer T can dimerize to DynT_2_, which can further react to form other oligomers (DynT_*n*_; Fig. 2b and Extended Data Fig. 1). Alternatively, fuel-driven oligomerization can also occur when oligomers react with one another. We designed the six-atom repeat in the backbone of the oligoanhydride with a bond length of 7.14 Å such that it matches the six-atom length of the backbone of the repetition unit of RNA or DNA with a bond length of 7.65 Å (Fig. 2a, Supplementary Table 1 and Supplementary Notes). Our monomer T interacts with (dA)_10_ DNA templates with a dissociation constant *K*_D_ of 91.9 ± 12.7 mM per binding site determined via an NMR titration, which is about threefold lower than the *K*_D_ value we measured for deoxythymidine monophosphate (dTMP; Extended Data Fig. 2a,b)^21^. It is likely that T binds more strongly due to the additional stacking of the isophthalic acid backbone and adenosine units of the template.

**Fig. 3:**
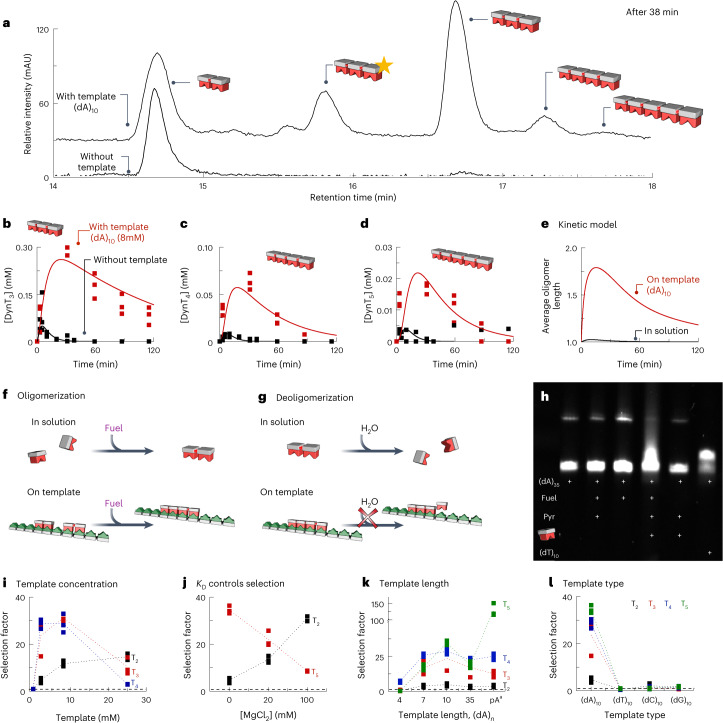
The template controls the kinetics of the chemically fuelled dynamic combinatorial library. **a**, HPLC chromatogram of the library with and without 8 mM template (dA)_10_ after 38 min in the reaction cycle. The yellow star denotes DynT_3_*, the unwanted N-acylisourea side product of DynT_3_. mAU, milli-absorbance units. **b**–**d**, The concentration (denoted by the square brackets) of oligomers DynT_3_ (**b**), DynT_4_ (**c**) and DynT_5_ (**d**) as a function of time with and without 8 mM template (dA)_10_. A kinetic model was used to fit the data. **e**, The kinetic model calculated the average oligomer length for the template and solution fractions in the presence of template (dA)_10_ (8 mM). **f**,**g**, Schematic representation of template-based selection for oligomerization (**f**) and deoligomerization (**g**). **h**, The gel of (dT)_10_ and DynT oligomers with (dA)_35_ in native gel electrophoresis. Dots on the gels are dust inclusions. The weaker but slow migrating band most probably originates from the self-association of (dA)_35_ at lower pH, high salt and high DNA concentrations^30^. Pyr, pyridine. In **a**–**d**,**f**–**h**, the pictograms for thymine oligomers are coloured red and the DNA template is coloured green. **i**–**l**, Selection factor *S*_31_ of the oligomers as a function of the concentration of the added template (dA)_10_ (**i**), MgCl_2_ concentration in the presence of template (dA)_10_ (**j**), template length for 2 mM (dA)_*n*_ (*n* = 4, 7, 10, 35) and polyadenine RNA pA (**k**) and template type for 2 mM (dX)_10_ (X = A, T, C, G) (**l**). Dotted lines are used to guide the eye. The labels C_2_, TC, C_3_ and the others denote DynC_2_, DynTC, DynC_3_ and so on, respectively. ^a^RNA was used instead of DNA. Source data

We dissolved 25 mM T in 200 mM MES-buffered water at pH 6 with 10 mM pyridine and added 10 mM EDC. Using HPLC and mass spectrometry, we analysed the evolution of the library composition in the presence and absence of a DNA template comprising ten adenosine units ((dA)_10_; Fig. 2a and Supplementary Tables 2–4). Without the template, we found that the fuel was consumed entirely after 30 min (Supplementary Fig. 1b), resulting in the transient oligomers DynT_2_–DynT_5_ (Fig. 3a and Supplementary Table 3). It is noteworthy that we did not observe oligomers greater than pentamers, even with higher fuel or lower pyridine concentrations. The major component was anhydride DynT_2_. By contrast, DynT_3_ had a roughly 19-fold lower maximum concentration at 0.05 mM. The maximum concentrations of DynT_4_ and DynT_5_ were even lower at 0.006 mM and 0.004 mM, respectively (Fig. 3b–d and Supplementary Fig. 1a). The maximum concentration decays with the oligomer length for two reasons. First, the product of one reaction is the precursor for the next; for example, DynT_2_ is needed for oligomerization towards DynT_3_. Second, the hydrolysis of higher oligomers yields shorter oligomers. As soon as all fuel was depleted, all oligomers had decayed, that is, the library exists only out of equilibrium and at the expense of chemical energy (Fig. 3b–d and Supplementary Fig. 1a,b). After the cycle, we found some unwanted side product *N*-acylisourea (T*), but its concentration remained below 0.5 mM (Supplementary Fig. 1c and Supplementary Table 4).

We devised a kinetic model to describe the time-dependent concentrations of all reactants in response to the fuel (see Methods and Extended Data Fig. 1). The model considers the fuel-driven activation and spontaneous hydrolysis of oligomers. Through least-squares fitting of the rate constants, the kinetic model predicted the experimental data of the evolution of fuel and oligomers well. Noteworthily, we observed that the oligomerization and deoligomerization were dependent on the oligomer length (Fig. 3b–d, Supplementary Fig. 1a,e–o and Supplementary Table 5).

We tested the response of the chemically fuelled library, now in the presence of a template. With the (dA)_10_ template, DynT_2_ was also the predominant oligomer early in the cycle (Fig. 3a). However, when all of the fuel had been consumed, the maximum concentrations of longer oligomers, for example, DynT_3_, DynT_4_ and DynT_5_, were higher by fivefold, 13-fold and sevenfold, respectively, compared to without the template (Fig. 3b–d and Supplementary Fig. 1b). Moreover, we found that DynT_2_–DynT_5_ are present for between one and two hours longer than in the experiments without the template. From these observations, we conclude that the template strongly influences oligomerization and deoligomerization (Fig. 3f,g, respectively). We hypothesize that the feedback mechanism of the template on oligomerization involves the pre-organization of oligomers on the template akin to the template-based ligation of DNA or RNA^22–27^. We also hypothesize that the template protects the oligomers from deoligomerization (vide infra). Interestingly, similar template-based protection has been reported for RNA, that is, double-stranded RNA is more stable than single-stranded RNA^28^, and has previously been explored as a mechanism to promote sequence copying^29^.

**Extended Data Fig. 3:**
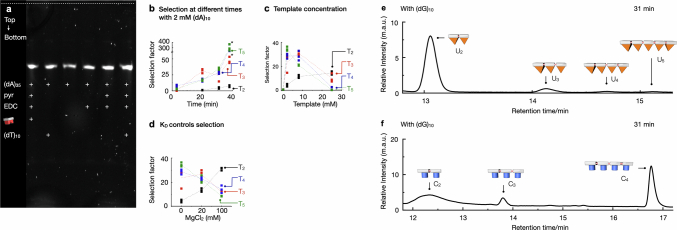
The template controls the kinetics of the chemical-fueled dynamic combinatorial library. **(a)** The gel of (dT)_10_ and **DynT** oligomers with (dA)_35_ in denaturing gel electrophoresis. Dots on the gels are dust inclusions. **(b)** Selection factor as a function of time in the presence of 2 mM template (dA)_10_ (expressed in monomer concentration). * **DynT**_**4**_ and **DynT**_**5**_ were not observed without template (dA)_10_ after 38 min. Their selection factors were calculated assuming concentrations of those oligomers below the detection limit of LC-MS. **(c-d)** Selection factor S_31_ of oligomers as a function of added **(c)** template concentration (dA)_10_, and **(d)** MgCl_2_ concentration in the presence of template (dA)_10_. **(e, f)** HPLC chromatogram of the **U (e)** or **C (f)** library with 2 mM template (dG)_10_ (expressed in monomer concentration) after 31 minutes in the reaction cycle. In b–f, the labels T_2_, T_3_, T_4_, T_5_, U_2_, U_3_, U_4_, U_5_, C_2_, C_3_, and C_4_ denote DynT_2_, DynT_3_, DynT_4_, DynT_5_, DynU_2_, DynU_3_, DynU_4_, DynU_5_, DynC_2_, DynC_3_, and DynC_4_, respectively. Source data

Mass spectrometry showed evidence of the complexes made from (dA)_10_ with monomer T and oligomers DynT_2_–DynT_5_ (Supplementary Table 6). Moreover, native gel electrophoresis showed a broadening and shifting towards higher molecular weights of the template band in the presence of DynT oligomers (Fig. 3h and Supplementary Table 7). For pure (dA)_35_ and mixtures that lack (dT)_10_, we observed a weaker but slow migrating band, most probably originating from the self-association of (dA)_35_ at lower pH, high salt and high DNA concentrations^30^. It is noteworthy that the bands of oligomer/template complexes do not shift under denaturing conditions, corroborating that our observations originate from hybridization (Extended Data Fig. 3a).

We extended our kinetic model to include the role of templation by adding reactions that can occur on the template. The model predicts the concentration of each library member in solution and on the template, using the dissociation constant *K*_D_ values between the library members and the template (see Supplementary Notes, Fig. 3b–d and Supplementary Fig. 1a–d). For the monomer, we used the experimentally determined value of the dissociation constant. We assumed that the binding constant decreases with increasing oligomer length, in line with the literature (Supplementary Table 8)^31^. We used the same rate constant values for oligomerization and deoligomerization in the solution and on the template that we used before. However, the kinetic model underestimates the concentration and lifetime of the oligomers. The experimental data can only be predicted well if we assume no deoligomerization on the template (Fig. 3b–d and Supplementary Figs. 1a–d and 2). This first observation concludes that hybridization protects from hydrolysis, which aligns with our experimental observations that oligomers survive longer with the template (Fig. 3g).

**Extended Data Fig. 4:**
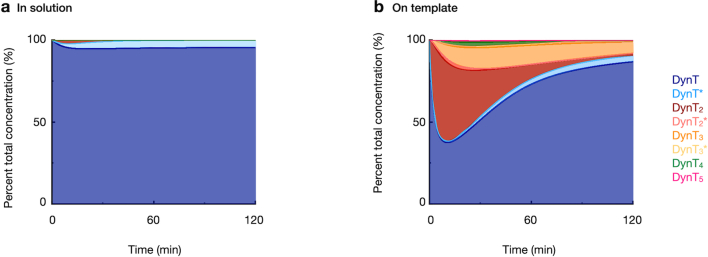
Dynamic library composition in solution and on the template. **(a, b)** The kinetic model calculated the fraction of library members in solution **(a)** and on the template **(b)** during the reaction cycle. Source data

Furthermore, the model predicts that library members in solution consumed 90.3% of the EDC, whereas library members on the template consumed only 9.7% of the EDC (Supplementary Fig. 3). Given the high monomer concentration in the solution, most of this 90.3% of the fuel was used to form DynT_2_. Specifically, in presence of the template, the average length in the solution was 1.02, consistent with the observation that monomers dominate the library (Fig. 3e and Extended Data Fig. 4a). By stark contrast, the average length on the template was 1.78, resulting from a high amount of dimers, trimers and tetramers (Fig. 3e and Extended Data Fig. 4b). In other words, oligomers accumulate on the template because they have a lower dissociation constant than monomers^31^. Therefore, ligations between oligomers occur frequently on the template (Fig. 3f, on template). Consequently, even though library members on the template consume far less EDC, on average, each ligation produces an oligomer of higher length than when in the solution.

**Extended Data Fig. 5:**
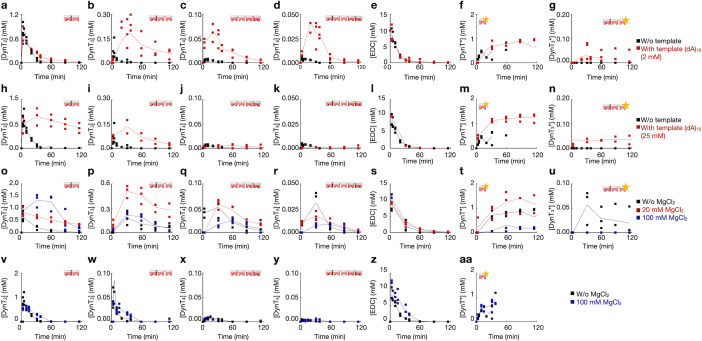
Kinetic characterization of the dynamic library as a function of (dA)_10_ or with varying [MgCl_2_] with and without template (dA)_10_. Concentration as a function of time for **(a-d, h-k, o-r, v-y)** oligomers **DynT**_**2**_-**DynT**_**5**_, **(e, l, s, z) EDC**, side products (f, m, t, aa) DynT*, and **(g, n, u) DynT**_3_* with **(a-g)** 2 mM and **(h-n)** 25 mM template (dA)_10_, at varying [MgCl_2_] **(o-u)** with 2 mM (dA)_10_, template, and with and without **(v-aa)** 100 mM MgCl_2_. Template concentrations are expressed in monomer concentration. Lines are guides for the eye, showing the evolution of the mean. Source data

We introduce the selection factor (*S*) to quantify templated oligomer production. For example, *S*_31_ is the factor between the oligomer concentration determined via HPLC with and without the template 31 min after adding the fuel. At low template concentrations, that is, 2 mM (dA)_10_ expressed in monomer units, the *S*_31_ value for DynT_4_ was roughly 28, whereas it was around five for DynT_2_. In other words, the template’s presence increased the concentration of DynT_4_ by more than 28-fold. Noteworthily, *S*_20_ and *S*_38_ showed the same trend, implying that the selection trend is generalizable in a time window (Extended Data Fig. 3b). The experiments contained T at 25 mM, so the template-to-library-member ratio was 0.08. Increasing the template concentration to 8 mM (dA)_10_ increased the *S*_31_ value of DynT_2_–DynT_4_ but not DynT_5_ (Fig. 3i and Extended Data Fig. 3c). The decrease in DynT_5_ is not entirely surprising, as the fifth base pair starts to turn in a B-DNA helix with a helical turn every 10.5 base pairs^32^. It seems that the oligomers cannot accommodate a twisted DNA angle, thus favouring shorter oligomers. Surprisingly, when we increased the template concentration further, for example, to 25 mM (dA)_10_, *S*_31_ decreased drastically for DynT_3_–DynT_5_ but not for DynT_2_—more template leads to shorter oligomers (Fig. 3i and Extended Data Fig. 5).

The positive feedback mechanism can explain these initially counterintuitive observations. The experiments are performed with substoichiometric amounts of fuel, that is, for each monomer, there are only 0.4 equivalents of fuel available. How this fuel is used will affect the oligomer length distributions, for example, one fuel molecule can link to monomers to form a dimer or two dimers to form a tetramer. The presence of the template affects this ‘efficiency’ of fuel usage. Without a template, the chance that two oligomers react to form a longer oligomer is small because the oligomer concentration is low. At a low template concentration, the library members compete for binding sites. This competition favours longer oligomers with higher binding constants to bind. Thus, the average oligomer length on the template is high, which favours the oligomerization of oligomers. At a higher template concentration, more binding sites are available, giving more space for monomers. Effectively, more template dilutes the oligomers on the template. This decreases the chance of two oligomers oligomerizing. In other words, too high a template concentration reduces the selective oligomerization of longer oligomers.

If the above mechanism is true, increasing the binding constant of the monomer should weaken the positive feedback of the template on the production of longer oligomers. Thus, we added the salt MgCl_2_, which is known to decrease the dissociation constant in native DNA^33–37^. In line with our expectation, the *S*_31_ value of DynT_5_ decreased gradually with increasing MgCl_2_ concentration, whereas more DynT_2_ was formed under these conditions (Fig. 3j and Extended Data Fig. 3d). Note that our control experiments without the template with and without MgCl_2_ show that MgCl_2_ does not affect the concentration of oligomers DynT_2_–DynT_5_. The individual kinetic profiles are shown in Extended Data Fig. 5.

**Extended Data Fig. 6:**
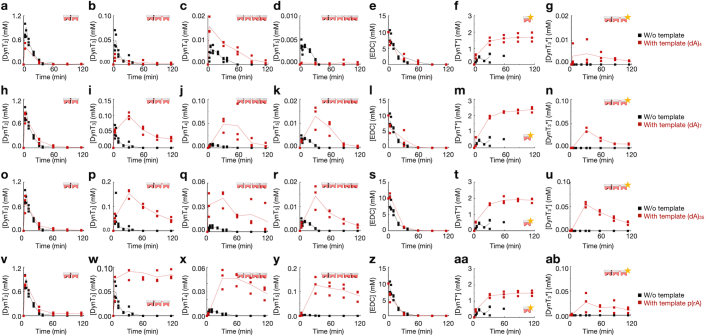
Kinetic characterization of the dynamic library with different template lengths. Concentration as a function of time for **(a-d, h-k, o-r, v-y)** oligomers **DynT**_**2**_-**DynT**_**5**_, **(e, l, s, z) EDC**, side products (f, m, t, aa) DynT*, and **(g, n, u, ab) DynT**_3_* with 2 mM template **(a-g)** (dA)_4_, **(h-n)** (dA)_7_, **(o-u)** (dA)_35_ and **(v-ab)** p(rA) (expressed in monomer concentration). Lines are guides for the eye, showing the evolution of the mean. Source data

On the basis of the above mechanism, we expect the template length to affect the length distribution of the dynamic library. To prove this, we tested DNA templates of different lengths, that is, (dA)_4_, (dA)_7_, (dA)_35_ and RNA-based polyadenine (pA; Fig. 3k and Extended Data Fig. 6). We analysed the changes in oligomer composition by monitoring *S*_31_. Interestingly, with template (dA)_4_, DynT_5_ was almost completely suppressed, whereas DynT_4_ was still formed. This seems to suggest that the template length controls the maximum length of the oligomer obtained (Fig. 3k and Extended Data Fig. 6a–d). Increasing the template length to (dA)_7_, (dA)_10_ and (dA)_35_ or switching to polyadenine RNA not only recovered the formation of DynT_5_ but increased the concentration of all oligomers DynT_2_–DynT_5_, and thus their selection factor values by factors of 3–5 for DynT_2_ and 16–35 for DynT_3_–DynT_5_ (Fig. 3k and Extended Data Fig. 6). It is of note that adding polyadenine RNA selectively increased the concentration of DynT_5_, by factors of 44 and 128.

**Extended Data Fig. 7:**
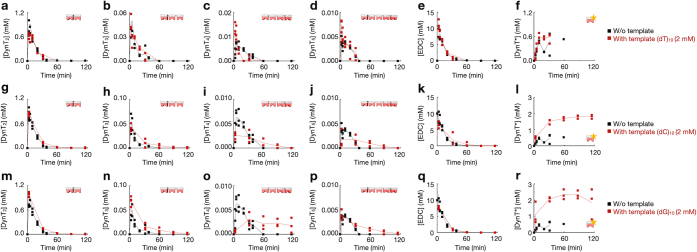
Kinetic characterization of the dynamic library with different types of templates. Concentration as a function of time for **(a-d, g-j, m-p)** oligomers **DynT**_**2**_-**DynT**_**5**_, **(e, k, q) EDC**, and side product (f, l, r) DynT* with 2 mM template **(a-f)** (dT)_10_, **(g-l)** (dC)_10_ and **(m-r)** (dG)_10_ (expressed in monomer concentration). Lines are guides for the eye, showing the evolution of the mean. Source data

Wobble base pairs, such as G•T, G•U or A•C base pairing, can lead to nucleobase mutations of the copied strands in DNA replication^38,39^. We tested if our synthetic system suffered from ‘wobble’ structures, too, by testing the oligomerization of T monomers in the presence of non-matching templates with the thymine ((dT)_10_), cytosine ((dC)_10_) and guanine ((dG)_10_) recognition motifs. We found only minimal changes in the selection factor, indicating that wobble base pairing does not play a large role in the case of DynT_n_ (Fig. 3l and Extended Data Fig. 7).

**Fig. 4:**
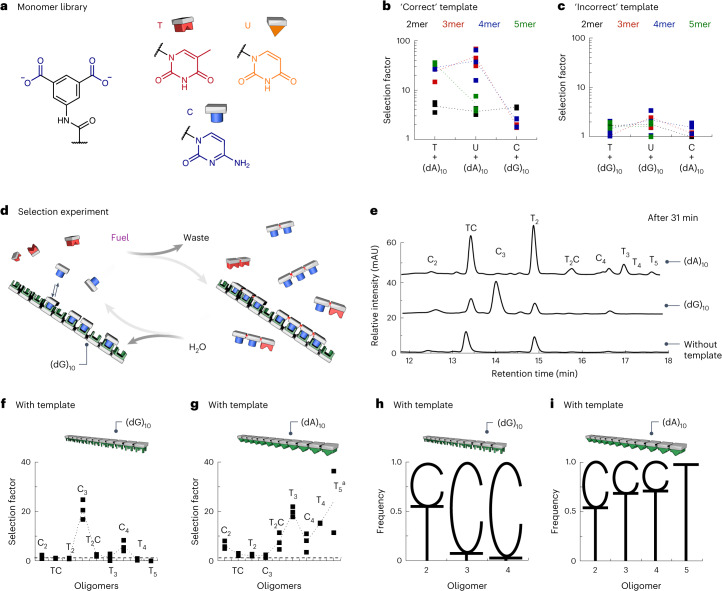
Selecting pairing nucleobases from non-pairing nucleobases. **a**, Molecular structures of monomers T, U and C. The reactive molecule sites, recognition motifs (for example, thymine) or complete molecular structures are colour-coded as follows: monomer acid (blue, left), thymine (red), uracil (orange) and cytosine (blue, right). **b**,**c**, Selection factor *S*_31_ for correct (**b**) and incorrect (**c**) templates (dG)_10_ and (dA)_10_ (at 2 mM) when fuelling with T, U or C. 2mer, dimer; 3mer, trimer; 4, tetramer; 5mer, pentamer. **d**, The chemical reaction system converts a chemical fuel (EDC) into waste (EDU) while building up mixed oligomers from monomers T and C. **e**, HPLC chromatogram at 31 min of the T/C oligomers without any template (bottom) and with 2 mM of template (dA)_10_ (top) or (dG)_10_ (middle). **f**,**g**, Selection factor *S*_31_ for the (dG)_10_ (**f**) and (dA)_10_ (**g**) template (at 2 mM). **h**,**i**, Sequence logo of the mixed T/C oligomers at 31 min in the reaction cycle with 2 mM of the templates (dG)_10_ (**h**) and (dA)_10_ (**i**). In **b**,**c**,**f**,**g**, the dotted lines are used to guide the eye. In **d**,**f**–**i**, the pictograms for the thymine oligomers are coloured red, the cytosine oligomers are coloured blue and the DNA template is coloured green with different shapes for (dA)_10_ (downtriangle) and (dG)_10_ (U shape). In **e**–**g**, the labels C_2_, TC, C_3_ and the others denote DynC_2_, DynTC, DynC_3_ and so on, respectively. ^a^DynT_5_ was not observed without template (dA)_10_. Source data

Next, we focused on other monomers besides T. We fuelled libraries with U (uracil) or C (cytosine) isophthalic monomers in the presence of (dA)_10_ and (dG)_10_. Noteworthily, the purine bases (G and A) were poorly soluble when attached to isophthalic acid and thus were not used. Fuelling with U, a demethylated version of T, a diverse oligomer library emerges, comprising DynU_2_–DynU_5_ oligomers (Extended Data Fig. 3e and Supplementary Tables 9 and 10). In the presence of the ‘correct’ template (dA)_10_, the oligomer yield and lifetime of DynU_2_–DynU_5_ increased. By contrast, the ‘incorrect’ template (dG)_10_ brought about only a slightly increased yield and lifetime of DynU_3_–DynU_5_ (Fig. 4a–c and Supplementary Fig. 4a–l). When fuelling with C, a diverse oligomer library emerged, comprising DynC_2_, DynC_3_ and DynC_4_ (Fig. 4a–c, Extended Data Fig. 3f and Supplementary Tables 11 and 12). Again, using the correct template (dG)_10_, the oligomer yields increased. However, the C library suffered from wobble base pairing, as the presence of (dA)_10_ increased the oligomer yields, too (Fig. 4a–c and Supplementary Fig. 4m–v).

### Selecting pairing nucleobases from non-pairing nucleobases

**Extended Data Fig. 8:**
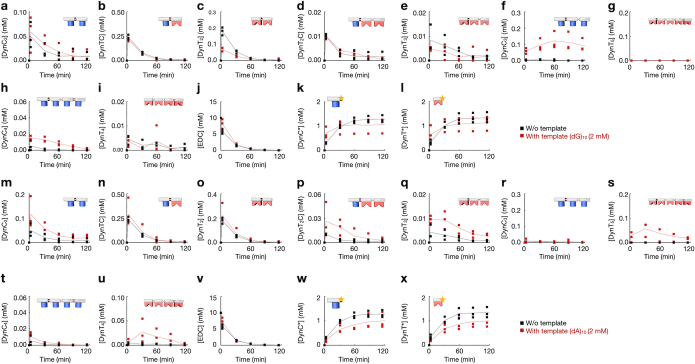
Kinetic characterization of the C/T dynamic library with different types of templates. Concentration as a function of time for **(a-i, m-u)** oligomers **DynC**_**2**_, **DynCT**, **DynT**_**2**_, **DynC**_**3**_, **DynCT**_**2**_, **DynT**_**3**_**, DynC**_**4**_, **DynT**_**4**_, and **DynT**_**5**_ **(j, v) EDC**, side products (k, w) DynC*, and (l, x) DynT* with 2 mM template **(a-l)** (dG)_10_, and **(m-x)** (dA)_10_ (expressed in monomer concentration). Lines are guides for the eye, showing the evolution of the mean. Source data

Given the selective oligomerization of hybridizing library members, we tested whether we could select T monomers from C monomers (Fig. 4d). We dissolved T (at 12.5 mM) and C (at 12.5 mM) in a similar buffer as described above and added a batch of 10 mM EDC. Without any template, we found eight oligomers: the three dimers DynT_2_, DynC_2_ and DynTC (DynCT and DynTC are the same molecules), three trimers (DynC_3_, DynT_3_ and DynT_2_C) and the two tetramers DynT_4_ and DynC_4_ (Fig. 4e and Supplementary Tables 3, 4 and 11–13). The dimers DynT_2_, DynC_2_ and DynTC were the major oligomers in the first minutes, of which the mixed dimer DynTC had the highest yield (Extended Data Fig. 8a–c), which is unsurprising because of its two formation pathways. The trimers and tetramers are in the same concentration range at around 0.01–0.02 mM, whereas pentamers were not observed (Extended Data Fig. 8d–j).

With template (dG)_10_ at 2 mM (expressed as the monomer concentration), the library composition remained similar for all oligomers apart from DynC_3_ and DynC_4_, whose concentrations increased from fourfold to 20-fold (Fig. 4e,f and Extended Data Fig. 8a–j). Interestingly, with template (dA)_10_ at 2 mM (expressed as monomer concentration), all of the oligomer concentrations increased. In particular, the concentrations of DynT_2_C, DynT_4_ and DynT_5_ increased drastically. As DynT_5_ was not observed without a template, its *S*_31_ value would be greater than 30 if we assumed concentrations below the detection limit of the liquid chromatography–mass spectrometry (LC–MS) measurements. Noteworthily, the concentrations of DynC_2_ and DynC_5_ increased roughly five and 20-fold, respectively, upon templating with (dA)_10_ (Fig. 4e,g and Extended Data Fig. 8m–x). We hypothesize that selection suffers from wobble base pairing, especially for A•C, which we have already demonstrated above. The sequence logos in Fig. 4h,i describe the relative frequency of monomers T and C within the oligomer groups (dimers, trimers, tetramers and pentamers) with the templates (dG)_10_ and (dA)_10_. In the presence of the templates, the dimers comprise roughly 55% T and 45% C. In the presence of template (dG)_10_, monomer T is less favoured (5%) in trimers and tetramers, whereas in the presence of template (dA)_10_, C is less favoured in tetramers (28%) and pentamers (0%). Taken together, the selective oligomerization of hybridizable monomers and their protection on the template increases the incorporation of T or C monomers into longer oligomers, respectively, for the correct template. However, selection for C suffers particularly from A•C wobble base pairing.

### Purifying nucleobases from mixtures

We wondered whether the selective binding of certain oligomers could be used to purify pools of monomers. The hypothesis is that a mixture of monomers becomes purer after a templated oligomerization–deoligomerization cycle, driven by different binding affinities and subsequent phase separation of the template–oligomer complexes^21,31,40,41^. Mechanisms here could also shine new light on purification mechanisms in a prebiotic setting—the prebiotic formation of the first nucleosides was unlikely to yield pure A, C, T and U, and purification mechanisms such as template-assisted ligation and selective precipitation probably played a role^42–46^.

**Fig. 5:**
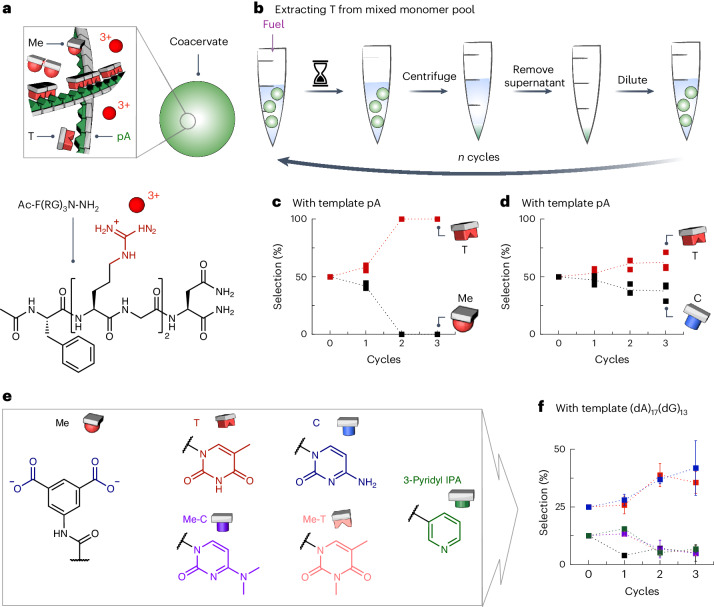
Extracting T from a mixed monomer pool. **a**, Coacervate droplets (green droplets) made from Ac-F(RG)_3_N-NH_2_ (shown as red spheres with a charge of +3; the cationic side groups of the molecular structure are highlighted in red) and pA (green pictogram). **b**, Schematic of the experimental set-up to extract T from a mixed monomer pool. **c**,**d**,**f**, Composition of the coacervate phase after each cycle for the mixed T/Me pool (**c**), mixed T/C pool (**d**) and mixed T/C/Me-C/Me-T/Me/3-pyridyl IPA pool (**f**). Error bars depict the standard deviation of the mean (*n* = 18). **e**, Molecular structures of Me, T, C, Me-C, Me-T, 3-pyridyl IPA. The reactive molecule sites and recognition motifs (for example, thymine) are colour-coded as follows: monomer acid (blue, left), thymine (red), cytosine (blue, right), methylated cytosine (purple), methylated thymine (pink) and 3-pyridyl IPA (green). The respective monomer pictograms are coloured the same as before. The DNA template is coloured green. In **c**,**d**,**f**, the dotted lines are used to guide the eye. Source data

**Extended Data Fig. 9:**
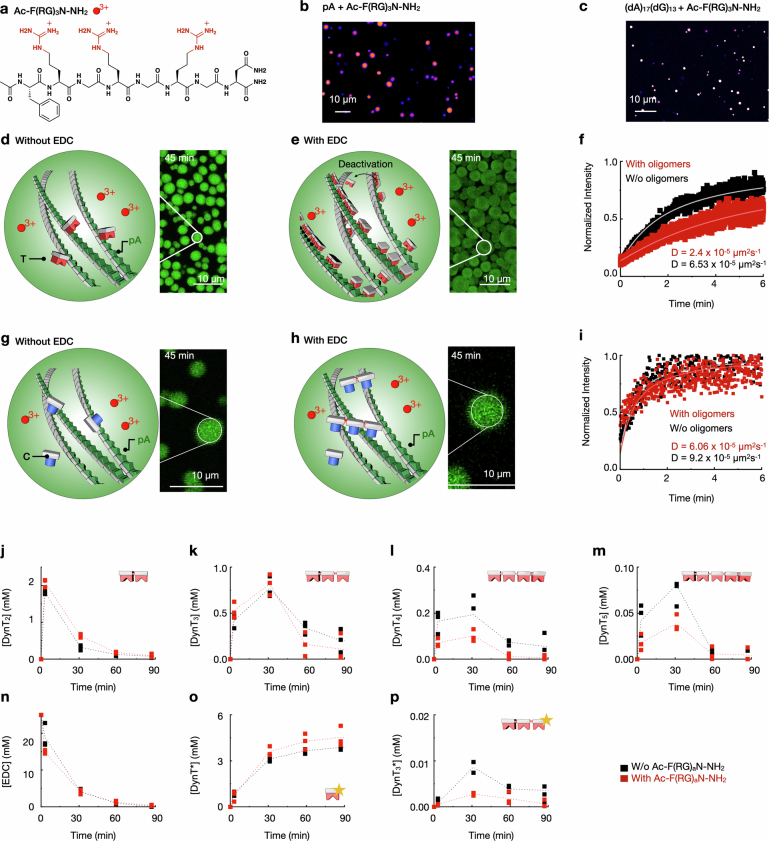
Changing physical properties of coacervate-based protocells. **(a)** Molecular structure of polycation Ac-F(RG)_3_N-NH_2_ (red spheres with the charge of 3+, cationic side groups are highlighted in red). **(b, c)** Complex coacervates formed with 5 mM Ac-F(RG)_3_N-NH_2_ and **(b)** 0.86 mM pA (expressed in monomer concentration) or **(c)** 0.6 mM (dA)_17_(dG)_13_ (expressed in monomer concentration) and 5 mM Ac-F(RG)_3_N-NH_2_ at pH 6, 200 mM MES buffer. Coacervates were stained with dye sulforhodamine B **(b)** and dye Cy5-(dU)_15_ **(c)**. **(d, e, g, h)** Schematic representation and confocal micrographs of coacervates droplets made from Ac-F(RG)_3_N-NH_2_ and pA, **T (d, e)** or **C (g, h)**, without **(d, g)** and with **(e, h)** fuel EDC. Coacervates were stained with dye Cy5-(dU)_15_. **(f, i)** FRAP recovery curves of Ac-F(RG)_3_N-NH_2_ and pA coacervates in the presence of **T (f)** or **C (i)**, pyridine **(d, g)** without, and **(e, h)** with EDC at 60 min. **(j-p)** Concentration as a function of time for **(j-m)** oligomers **DynT**_**2**_-**DynT**_**5**_, **(n) EDC**, side products (o) DynT*, and **(p) DynT**_3_* with and without 5 mM Ac-F(RG)_3_N-NH_2_ added to the library made from **T** fueled with 25 mM EDC with 4.31 mM pA and 5 mM pDexS. Source data

As a selection mechanism, we used the ability of pA (RNA) to form complex coacervates by mixing pA as the polyanion at 0.86 mM (expressed as monomer concentration) and the cationic peptide Ac-F(RG)_3_N-NH_2_ (at 5 mM) at pH 6 (Fig. 5a and Extended Data Fig. 9a,b; phenylalanine (F), arginine (R), glycine (G) and asparagine (N)). After applying 50 mM fuel, we centrifuged the reaction solution, removed the supernatant and resuspended the pellet by adding buffer. We homogenized the pellet via sonication and then reinitiated the cycle. The cycle was performed three times in total (Fig. 5b). After each cycle, we analysed the composition of the resuspended pellet via HPLC (Fig. 5c). After the first cycle, the droplet consisted of 55% T and 45% Me (that is, the monomer with no nucleobase recognition motif). After the second cycle, the library contained almost exclusively T. The third cycle did not change the composition further. We hypothesize that the inefficiency of the first cycle results from both monomers partitioning in the droplets non-selectively (log *P* is 1.51 ± 0.15 for T and 0.99 ± 0.15 for Me). Thus, after centrifugation, there is no selection after the first cycle. In the second cycle, the concentration of monomers is much lower because many monomers have been washed away in the first cycle, leading to more selective, templated uptake.

Next, we attempted purification between the pyrimidine nucleobases (T and C in Fig. 5d). Both monomers had similar partition coefficients of 1.51 ± 0.15 and 1.33 ± 0.16, respectively. After the first cycle, equal amounts of T and C were present in the coacervate phase. After the second cycle, the coacervate phase comprised roughly 60% T and 40% C (Fig. 5d). In other words, the selection was not particularly efficient, probably because of A•C wobble base pairing. We expanded the monomer pool further to six members, that is, T, C, Me, methylated T (Me-T), methylated C (Me-C) and a non-natural base of 3-pyridyl isophthalic acid (3-pyridyl IPA, Fig. 5e). The library consisted of 25% each of T and C and 12.5% each of Me, Me-T, Me-C and 3-pyridyl IPA. We used coacervate droplets formed with the template (dA)_17_(dG)_13_ (or 0.34 mM adenine and 0.26 mM guanine when expressed in monomer concentration) and 2.5 mM polycation Ac-F(RG)_3_N-NH_2_ at pH 6 (Extended Data Fig. 9c). After the first cycle, the library composition had hardly changed. By contrast, the second cycle decreased the concentrations of the methylated and non-natural nucleobases, whereas the concentrations of C and T were increased slightly to 37% and 39%, respectively. The third cycle did not change the library composition further (Fig. 5f). Finally, we carried out similar experiments using hydrogels that comprise pA instead of coacervate droplets. These hydrogels showed similar purification abilities (see Supplementary Notes and Supplementary Fig. 5). The ability to preferentially ligate monomers offers a simple mechanism for purifying libraries of monomers via phase separation and centrifugation.

### Template copying alters the system’s physical properties

We demonstrated how templation affects the library composition. Next, we tested whether the opposite can also be true, that is, can the library affect its medium? Such reciprocal coupling between sequences (such as DNA or RNA) and their environment (a protocell or the cell) brings us closer to exploring Darwinian evolution in our system. For example, the library’s presence could increase the likelihood of a droplet producing offspring or surviving^47–51^.

We measured the fluorescence recovery after photobleaching (FRAP) of complex coacervate droplets made of pA, the polycation peptide Ac-F(RG)_3_N-NH_2_ and monomers T or C before and after adding fuel. Before adding the fuel, FRAP experiments on the polyanion pA showed that micrometre-sized droplets recovered their fluorescence within minutes, from which we calculated the diffusion constant *D* of 6.53 × 10^−5^ µm^2^ s^−1^. By contrast, after adding the fuel, the diffusion constant decreased roughly threefold in the presence of T oligomers, whereas it decreased roughly 1.5-fold in the presence of C oligomers (Extended Data Fig. 9d–i). Thus, the internal viscosity increases drastically upon fuel-driven oligomerization, probably related to triple helices with pA, which have a larger persistence length and act as cross-linkers^52–57^. For the mismatching nucleobase oligomers C, the effect seems to be smaller.

**Fig. 6:**
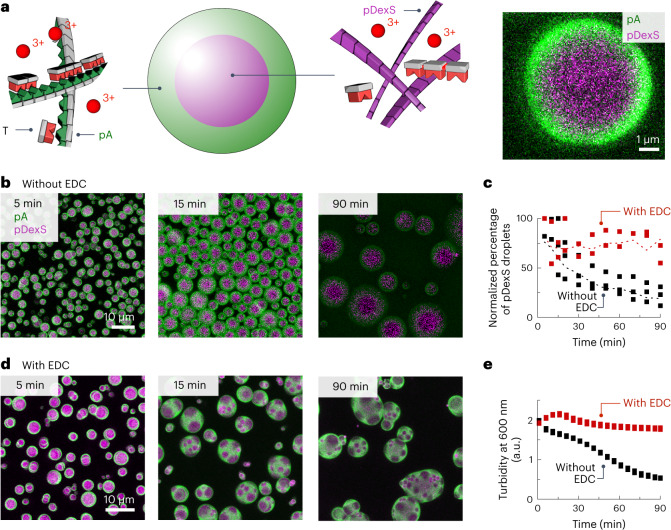
Microscopy analysis of coacervate droplets. **a**, Schematic representation of multiphase coacervates, with a pDexS core (purple) and a pA shell (green). The red spheres with the change of +3 represent the cationic peptide Ac-F(RG)_3_N-NH_2_. The pictograms for the thymine oligomers are coloured red, the DNA template is coloured green, pDexS is coloured purple and the coacervate is coloured a lighter green. **b**,**d**, Confocal micrographs of coacervate droplets made from Ac-F(RG)_3_N-NH_2_, pDexS, pA and T, without (**b**) and with (**d**) fuel. **c**, The normalized percentage of pDexS droplets with and without fuel. Dotted lines are used to guide the eye. **e**, Turbidity of coacervate droplets made from Ac-F(RG)_3_N-NH_2_, pDexS, pA and T, with and without fuel, reflects the fusion of the droplets. Source data

**Extended Data Fig. 10:**
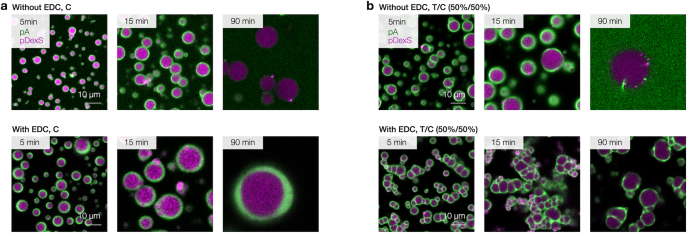
The template-oligomer composition controls the physical properties of its compartment. Confocal micrographs of coacervates droplets made from Ac-F(RG)_3_N-NH_2_, pDexS, pA, and **(a) C**, and **(b) T/C** (50%:50%), with and without fuel at 5 min, 15 min, and 90 min after adding 25 mM EDC. Source data

Coacervate-based droplets are known to fuse rapidly, which disadvantages a protocell as it loses its identity. The decrease in viscosity prompted us to explore if template-based copying could decrease fusion between protocells. For the protocells, we used the polyanions pDexS and pA. We mixed those with the cationic peptide Ac-F(RG)_3_N-NH_2_ and monomer T. We obtained multiphase complex coacervate droplets comprising a pDexS core and pA shell (Fig. 6a), which fused from roughly 130 multiphase droplets in a selected area to 19 droplets within 90 min (Fig. 6b,c). Their pA shell fused first, after which the pDexS core fused. The same experiment with fuel resulted in similar multiphase droplets in which the pA shell still fused. However, the increased viscosity resulted in a drastic decrease in the fusion of the pDexS cores, which led to large pA droplets with multiple pDexS cores inside them (Fig. 6c,d). By contrast, the same experiment with C instead of T did not decrease the fusion of the pDexS cores (Extended Data Fig. 10a). Mixing C and T in a 1:1 ratio, however, recovered the protection mechanism by which the pDexS cores stopped fusing (Extended Data Fig. 10b).

Turbidity measurements (Fig. 6e) further confirmed our confocal dataset shown in Fig. 6b,d. The turbidity of the multiphase droplets decayed, reflecting their fusion. By contrast, the turbidity did not decay when there was a drastic decrease in the fusion of the pDexS cores. It is noteworthy that the cationic peptide did not impact the DynT_2_ and DynT_3_ concentrations but decreased the concentrations of the DynT_4_ and DynT_5_ oligomers slightly (Extended Data Fig. 9j–p). The combined datasets show that template-based copying can change the phenotype of the protocell, in this case, its tendency to fuse.

## Conclusion

We developed a chemically fuelled dynamic combinatorial library to help unveil the mechanisms of selection of unstable, information-containing oligomers. We find that hybridization accelerates oligomerization by selectively concentrating longer oligomers. It simultaneously decelerates deoligomerization through a protection mechanism. These feedback mechanisms can favour oligomers of specific lengths and sequences, which can be used to purify libraries of oligomers. Finally, we showed that template-based copying changes the physical properties of coacervate-based protocells. This coupling between information-containing sequences and their compartments is a hallmark of life as we know it. With such models, although the molecules that are used are not prebiotically relevant, we foresee valuable insights into the fundamental mechanisms of minimalistic life, which is helpful for the synthesis of de novo life and for the mechanism that could have operated at its emergence. Furthermore, our system provides new tools for the transfer of macromolecular information, where such tools have been proved to be beneficial in applications such as cryptography^58,59^ and molecular data storage^60–64^. In future work, we will focus on such practical and functional aspects of fuel-driven dynamic combinatorial libraries and will broaden the scope of our system by introducing replication, mutation and eventually evolution in our system.

## Methods

### General sample preparation

All experiments were conducted in 200 mM MES-buffered water, pH 6 (T library) or 6.5 (C library and mixed T/C library) at 25 °C, made from nuclease-free Milli-Q water (MQ water).

Stock solutions of T, U, Me and Ac-F(RG)_3_N-NH_2_ were prepared by dissolving the acids in 200 mM MES-buffered water at pH 6 to give a final concentration of 50 mM (T, U, Me) and 100 mM (Ac-F(RG)_3_N-NH_2_). Stock solutions of C, Me-T and 3-pyridyl IPA were prepared by dissolving the acids in 400 mM NaOH solution before adding 500 mM MES-buffered water at pH 6.5 and nuclease-free water to give a final concentration of 50 mM in 200 mM MES-buffered water at pH 6.5. The pH of all stock solutions was adjusted to pH 6 or 6.5 using a 5 M sodium hydroxide solution. pDexS (at 83.3 mM, expressed in monomer concentration) was dissolved in nuclease-free MQ water and adjusted to pH 6 or 6.5 using a 5 M sodium hydroxide solution. The average length of pDexS was 42.72 (expressed in monomer units). Five hundred millimolar pyridine stock solutions, 1 M MgCl_2_ stock solutions, 3 M sodium acetate and 200 mM MES-buffered water were prepared by dissolving pyridine, MgCl_2_, sodium acetate and MES hydrate in nuclease-free MQ water, respectively. The pH of the 500 mM and 200 mM MES-buffered water was adjusted to pH 6 or 6.5 using sodium hydroxide pellets and 5 M sodium hydroxide solution. The stock solutions were stored at 8 °C in the fridge until further use. Stock solutions of EDC (500 mM or 3 M) and 400 mM benzylamine stock solutions were freshly prepared for all experiments by dissolving, respectively, EDC powder or benzylamine in nuclease-free MQ water.

Stock solutions of DNA oligomers, pA RNA (100–500 kDa) and RNA-based polyuracil (pU; 600–1,000 kDa) were prepared in nuclease-free MQ water to give a final concentration of 100 mM (DNA) and 41.3 mM (pA, pU; expressed as monomer concentration). Experiments were performed with 2, 8 and 25 mM DNA and 0.86, 2.00 and 4.31 mM pA or pU (expressed in monomer concentration). The average length of pA was 864 bases and of pU was 2,200 bases. The stock solutions were aliquoted and stored at −20 °C in the freezer until further use.

### Kinetic experiments

The experiments were carried out using micro inserts placed in 1.5 ml HPLC screw-cap vials (Sigma Aldrich) via the addition of 10 mM EDC to 25 mM T, U or C (40 µl), 10 mM pyridine and 0, 20 or 100 mM MgCl_2_ in 200 mM MES-buffered water at pH 6 (T, U) or pH 6.5 (C) in the presence or absence of the template. For mixed-library kinetics, 10 mM EDC was added to C and T (20 µl, each at 12.5 mM) and 10 mM pyridine, in 200 mM MES-buffered water at pH 6.5. All experiments were conducted at 25 ± 0.5 °C as the reaction system was relatively temperature-sensitive. Note that we added pyridine to suppress the formation of side product *N*-aclyisourea via the rearrangement of *O*-acylisourea^65^. The reaction kinetics of the chemical reaction system were monitored over time using analytical HPLC (vide infra). We identified oligomers by performing LC–MS measurements (vide infra; Supplementary Tables 3, 4 and 9–13).

### Kinetic model

The kinetic model is based on chemical rate equations, which are integrated numerically to compute the time evolution of the concentrations (Supplementary Notes). Unlike in stochastic simulations of combinatorial libraries^66,67^, where different compounds can appear or disappear dynamically, in chemical rate equations, all (relevant) chemical compounds need to be considered at each time step, which is memory-intensive. However, in the present context, the chemical rate equation approach has technical advantages: (1) a timescale separation between (de)hybridization and polymerization can be considered by solving a set of algebraic equations at each time step; and (2) the unknown rate constants can be estimated via standard least-squares curve fitting to the experimental data. Using this approach, we determined the rate constants for reactions in solution. Rate constants for reactions on the template were then varied to test for compatibility with the experimental data. Overall, the time-dependent concentrations of oligomers predicted by the resulting model agreed well with the experimental measurements. Additional observables, for example, the oligomer length distribution in solution and on the template, enabled us to identify the mechanisms of template-based oligomerization.

### Analytical reversed-phase HPLC

Concentration profiles of the chemical reaction systems were monitored via analytical reversed-phase HPLC (Thermo Fisher Dionex Ultimate 3000) using an Agilent HPLC column (Thermo Fisher Dionex Ultimate 3000, Hypersil Gold 250 × 4.8 mm). The samples were prepared as described using micro inserts placed in 1.5 ml HPLC screw-cap vials. The solutions were injected directly onto the column without further dilution (injection volume: 1 μl) and tracked using an ultraviolet/visible (UV/vis) detector at a wavelength (*λ*) of 220 nm (for EDC), 260 nm (pyridine, benzylamine, U, C, T/C oligomers, Me-T and 3-pyridyl IPA) or 290 nm (T, U and C side products). All compounds involved were separated using linear water and acetonitrile (ACN) gradients. Both eluents contained 0.1% trifluoroacetic acid. The water eluent contained 2% THF to decrease the peak broadening of the oligomers. The library components were eluted, applying an eluent flow rate of 1 ml min^−1^ and using the step-gradient HPLC method 1.

The purity of the recovered DNA oligomers (dA)_4_ and (dA)_10_ was measured using an analytical reversed-phase HPLC column (DNAPac RP, 4 µm, 2.1 × 100 mm). We applied a linear gradient of 0.1 M triethylammonium acetate and 0.1 M triethylammonium acetate/ACN (75:25 (v/v)). The DNA oligomers (dA)_4_ and (dA)_10_ were eluted, applying an eluent flow rate of 0.2 ml min^−1^, using HPLC method 2 with an injection volume of 2 µl and UV absorption at 260 nm.

The purity of the synthesized compounds T, U, C, Me, T-ester, Me-T, Me-C, 3-pyridyl IPA, (Boc)_2_-5-nitro-isophthalate and (Boc)_2_-5-amino-isophthalate was measured using HPLC method 1 or 3.

*HPLC method 1*. H_2_O:ACN from 98:2 to 72:28 in 14 min, from 72:28 to 65:35 in 4 min, from 65:35 to 50:50 in 2 min, from 50:50 to 2:98 in 2 min, 2:98 for 1 min, from 2:98 to 98:2 in 1 min and 98:2 for 4 min.

*HPLC method 2*. H_2_O:ACN from 100:0 to 50:50 in 25 min, 50:50 for 3 min, from 50:50 to 100:0 in 2 min and 100:0 for 10 min.

*HPLC method 3*. H_2_O:ACN from 98:2 to 2:98 in 13 min, 2:98 for 2 min, from 2:98 to 98:2 in 2 min and 98:2 for 3 min.

*HPLC method 4. H*_2_*O:ACN from 98:2 to 72:28 in 24* *min, from 72:28 to 65:35 in 4* *min, from 65:35 to 50:50 in 2* *min, from 50:50 to 2:98 in 2* *min, 2:98 for 11* *min, from 2:98 to 98:2 in 1* *min and 98:2 for 4* *min.*

### Preparative reversed-phase HPLC for purification

The crude products T-ester, T, U, C, Me-C, Me-T and 3-pyridyl IPA were purified using reversed-phase HPLC (Dionex Ultimate 3000, Hypersil Gold 250 × 4.8 mm, 5 μm pore size, C18 column) with a linear gradient of ACN and water, each with 0.1% trifluoroacetic acid.

*Preparative HPLC method*. H_2_O:ACN from 95:5 to 5:95 in 14 min, 5:95 for 2 min, from 5:95 to 95:5 in 1 min and 95:5 for 6 min.

### Determination of calibration values

Calibration curves for EDC (*λ* = 220 nm), pyridine (*λ* = 260 nm), T (*λ* = 260 nm, 290 nm), U (*λ* = 260 nm, 290 nm), C (*λ* = 260 nm, 290 nm), Me (*λ* = 260 nm, 290 nm), Me-T (*λ* = 260 nm, 290 nm), Me-C (*λ* = 260 nm, 290 nm) and 3-pyridyl IPA (*λ* = 260 nm, 290 nm) were performed in triplicate to determine the HPLC calibration values (Supplementary Tables 2–4 and 9–13). Calibration was impossible for the following: T*, U*, Me* and C*, the *N*-acylisourea side products of T, U, Me and C, respectively; the oligomeric anhydrides of T, U and C; BA T, BA U and BA C, the monobenzylamides of T, U and C, respectively; and BA 2T, BA 2U and BA 2C, the bisbenzylamides of T, U and C, respectively. In these cases, we used the respective calibration values of T, U and C at *λ* = 290 nm. We assumed that the absorbance at *λ* = 290 nm results predominantly from the aromatic ring of T, U and C, respectively, whereas benzylamine does not absorb at 290 nm. Evidence of our hypothesis is that the calibration value of the monobenzylamide of isophthalic acid is identical to the calibration value of isophthalic acid^17^.

We calibrated the anhydrides DynT_2_–DynT_5_, DynU_2_–DynU_5_ and DynC_2_–DynC_4_ by synthesizing these library members in situ, separating them via analytical HPLC, collecting the fractions and measuring the concentration of T, U and C for these fractions after hydrolysis. In detail, we fuelled the respective monomer (at 25 mM) using 25 mM EDC. We injected 8 µl of that reaction solution onto the reversed-phase analytical HPLC column three minutes after starting the reaction cycle. We collected the peak fractions of each oligomer and added 5 M sodium hydroxide solution (20 µl) to ensure complete hydrolysis of the anhydride to the monomer. Next, we injected 5 µl of the collected peak fractions onto the reversed-phase analytical HPLC column. We used the concentration that we obtained for the monomer to calculate back to the concentration of the respective anhydride in the collected fractions (Supplementary Fig. 6). That concentration, combined with the intensity of the peak on the chromatogram, was used to determine a calibration value. This procedure was repeated for different time points (3, 5 and 8 min) in the reaction cycle in triplicate to obtain a calibration value over a wider range of concentrations. We did not perform the calibration for mixed oligomers T and C. For the mixed oligomers, we took the calibration value of the respective T and C oligomers and added them proportionally, for example, for DynTC, we added 50% T and 50% C.

### Liquid chromatography–mass spectrometry

LC–MS experiments were conducted using an LCQ Fleet Ion Trap mass spectrometer (Thermo Scientific) or an Acquity Premier system (Waters). The samples were analysed in negative or positive modes. All recorded MS data were analysed using either the Thermo Xcalibur Qual Browser 2.2 SP1.48 software the LCQ Fleet Ion Trap mass spectrometer or the MassLynx V4.2 SCN1025 SCN1033 2021 software from Waters. The samples were injected directly onto an analytical reversed-phase column upstream of the three-dimensional ion trap (LCQ Fleet Ion Trap mass spectrometer). All compounds involved were separated by applying a flow rate of 0.7 ml min^−1^ (LC–MS method 1 or 2) or 0.4 ml min^−1^ (LC–MS method 3) and using linear gradients of water and ACN (linear gradient H_2_O:ACN from 95:5 to 5:95 in 8 min for purity checks; LC–MS method 1) for 20 min (library analysis; LC–MS method 2) or 15 min (library analysis, direct evidence of hybridization; LC–MS method 3). Both eluents contained 0.1% formic acid. The solutions were injected directly onto the column without further dilution (injection volume: 1 μl) and tracked via mass in negative or positive mode and with a UV/vis detector at 220 nm and 280 nm, respectively.

The mass error (in ppm) was calculated using free software from the Barrow group (https://warwick.ac.uk/fac/sci/chemistry/research/barrow/barrowgroup/calculators/mass_errors/).

*LC–MS method 1*. H_2_O:ACN 95:5 for 2 min to equilibrate the column before injecting, H_2_O:ACN from 95:5 to 5:95 in 7 min, from 95:5 to 0:100 in 1 min, 0:100 for 2 min, from 0:100 to 95:5 in 1 min and 95:5 for 1 min.

*LC–MS method 2*. H_2_O:ACN 95:5 for 4 min to equilibrate column before injecting, H_2_O:ACN from 95:5 to 5:95 in 19 min, from 95:5 to 0:100 in 1 min, 0:100 for 4 min, from 0:100 to 95:5 in 1 min and 95:5 for 1 min.

*LC–MS method 3*. H_2_O:ACN 95:5 for 1 min, H_2_O:ACN from 95:5 to 10:90 in 9 min, from 95:5 to 70:30 in 8 min, from 70:30 to 10:90 in 1 min, 10:90 for 2.5 min, from 10:90 to 95:5 in 1 min and 95:5 for 2.5 min.

### Matrix-assisted laser desorption/ionization–time of flight mass spectrometry (MALDI–TOF MS)

MALDI–TOF experiments were conducted using a MALDI SYNAPT XS mass spectrometer (Waters). The samples were analysed in negative modes. All recorded MS data were analysed using the MassLynx V4.2 SCN1025 SCN1033 2021 software. As the matrix, 30 mM α-cyano-4-hydroxycinnamic acid was used in 1:1 ACN:H_2_O. Twenty minutes after adding the EDC, the reaction solution was mixed in a 1:1 ratio with the matrix. A 10 µl aliquot of the reaction solution was filtered using a preactivated ZipTip C18 pipette tip (Merck). A 1 µl volume of the reaction solution was spotted onto the MALDI plate and dried in air.

### Time-lapse photography

Time-lapse software was programmed to image the sample in intervals of 60 s. All recorded images were analysed using ImageJ 1.52p software (Java 1.80_172 (64-bit)). Samples were prepared in a 1.5 ml HPLC vial for total volume of 200 µl.

### ^1^H NMR experiments

For purity analysis, ^1^H NMR experiments were conducted using Bruker AV400US (400 MHz) or Bruker AV300 AVIII (300 MHz) instruments. ^1^H NMR experiments for kinetic measurements and determination of the dissociation constant *K*_D_ were performed using a Varian Inova 300 (300 MHz) spectrometer. Chemical shifts are given as *δ* values in parts per million (ppm) relative to the internal standard 3-(trimethylsilyl)-1-propanesulfonic acid sodium salt (TPS; 0.05 mM) in D_2_O (*δ*_H_: 6.70) or the deuterated solvent peak of dimethyl sulfoxide-d_6_ (*δ*_H_: 2.50). For denoting the observed signal multiplicities, the following abbreviations were used: s (singlet), d (doublet), t (triplet) and m (multiplet).

^1^H NMR spectroscopy was used to determine the dissociation constant *K*_D_ by titrating T and Me to 5 mM (dA)_10_ and dTMP to 2.5 mM (dA)_10_ at 25 °C (expressed in monomer concentration). TPS (0.05 M) was used as the standard.

#### Calculation of *K*_D_

The dissociation constant was determined via ^1^H NMR titration in 200 mM MES-buffered D_2_O at pH 6, according to the following equation^31^:

1 $${\partial }_{{\rm{obs}}}=\frac{{\partial }_{\max }}{{K}_{{\rm{D}},10}}\times \frac{[{\rm{M}}]}{\left(1+\frac{[{\rm{M}}]}{{K}_{{\rm{D}},10}}\right)}$$

where *δ*_obs_ is the displacement of the chemical shift of the ^1^H signal, *δ*_max_ is the maximal displacement of the chemical shift and [M] is the monomer concentration. The chemical shift displacement was calculated for all shifting adenine ^1^H signals of (dA)_10_ (Extended Data Fig. 2). The obtained displacements were averaged. NMR titration was performed at least in duplicate.

The measured *K*_D,10_ value was multiplied by ten to give the *K*_D_ per binding site according to the following equation^68^:

2 $${K}_{n}=\frac{{K}_{{\rm{D}},10}\times (t-n+1)}{n}$$

where *K*_*n*_ is the *K*_D_ of the monomer (*n* = 1) per binding site, *t* is the number of possible binding sites and *n* is the ligand size (*n* = 1 here).

3 $${K}_{{\rm{D}},1}=\frac{{K}_{{\rm{D}},10}\times (10-1+1)}{1}=10\times {K}_{{\rm{D}},1}$$

### Confocal fluorescence microscopy

Confocal fluorescence microscopy was performed using a Leica TCS SP8 confocal microscope with a 63x water-immersed objective with a numerical aperture of 1.2. The pinhole was set to 1 Airy unit. Samples were imaged using a HyD detector, a pinhole of 111.4 µm and laser intensities of (1) 0.3 for the dye sulforhodamine B (0.2 μM) and (2) 1.0 for the dyes Cy5 (0.1 µM) and Cy3 (2 µM). The dyes sulforhodamine B and Cy3 were excited with a 552 nm laser and imaged at 563–625 nm. Dye Cy5 was excited with a 638 nm laser and imaged at 645–796 nm. Dyes were excited in sequential mode at 552 nm for dye Cy3-Dex or sulforhodamine B and at 638 nm for dye Cy5-(dU)_15_. Samples were prepared as described above with a total reaction volume of 20 μl directly in an ibidi µ-Slide angiogenesis well plate with a glass bottom. For droplet experiments, we used polyvinyl alcohol-coated ibidi µ-Slide angiogenesis well plates with a glass bottom. The fluorophore dyes were added before adding EDC. To analyse the coacervate evolution, time series were acquired in *z* stacks with 1 µm between the *z* planes (five planes in total). Recorded images were analysed using ImageJ 1.52p software (Java 1.80_172 (64-bit)). Imaging was performed at 22 °C.

### Fluorescence recovery after photobleaching

The viscosity of the coacervates made with Ac-F(RG)_3_N-NH_2_ and pA was measured by calculating the diffusivity inside the coacervates via FRAP experiments on the Cy5 dye of labelled polyanion pA. The samples were excited at 638 nm and detected between 640 and 780 nm with a photomultiplier tube detector. Coacervates at the bottom, close to the glass of the imaging chamber, were photobleached to minimize droplet movement during acquisition. A laser intensity of 0.1 with a pinhole of 222.9 µm was used to image the samples. Time series were acquired in one *z* plane to measure the initial fluorescence intensity and recovery after photobleaching. For photobleaching, a spot size with a diameter of 1 µm (for T with and without EDC) and 0.5 µm (for C with EDC) was chosen.

#### Calculation of diffusivity

Fluorescence recovery data were normalized according to the following equation derived from the double normalization equation by Wu and Allis from 2004^69^:

4 $$F(t)=\frac{{T}_{0}\times {I}_{t}}{{T}_{t}\times {I}_{0}}.$$

Here, *F*(*t*) is the normalized fluorescence intensity as a function of time *t*, *I*_*t*_ is the intensity of the bleached region of interest as a function of time *t*, *I*_0_ is the intensity of the bleached region of interest before photobleaching, *T*_*t*_ is the average intensity of the unbleached region of interest within the bleached coacervate as a function of time *t*, and *T*_0_ is the average intensity of the unbleached region of interest within the bleached coacervate before photobleaching. The following equation calculates fluorescence recovery^70^:

5 $$F(t)={F}_{\infty }\exp\left[-\frac{2}{1+\left(\frac{8tD}{{a}^{2}}\right)}\right]$$

where *F*_∞_ is the fluorescence at full recovery, *t* is the time, *a* is the bleached area and *D* represents the translational diffusion coefficient of the fluorescent probe^70^.

### Counting coacervates

Coacervate droplets were manually counted for image sizes of 62 × 62 µm using the multi-point tool provided by Fiji ImageJ.

### UV/vis spectroscopy

UV/vis measurements were carried out at 25 ± 0.5 °C using a Multiskan FC microplate reader (Thermo Fisher). Samples (50 μl) were prepared directly into a Greiner 96-well plate (half-area, UV-transparent). Each experiment was performed at 600 nm in triplicate.

### Gel electrophoresis

Native and denaturing polyacrylamide gel electrophoresis was used to analyse the DNA complexes using gels at 20% acrylamide concentration with a 37.5:1 acrylamide/bisacrylamide ratio (Bio-Rad) with 1x TB buffer, 10 mM MgCl_2_, 1x TBE buffer and 8.3 M urea. Gels were loaded with a final concentration of 350 nM DNA (expressed in monomer concentration), freshly prepared from 35 mM DNA stock solutions (expressed in monomer concentration; Supplementary Table 7) by diluting with 200 mM MES buffer, 100 mM NaCl and 12.5 mM MgCl_2_. NaCl and MgCl_2_ were added to prevent dissociation of (dA)_35_ with (dT)_10_ at low concentrations. We used 10% Ficoll 400 polymer in MQ water as a transparent loading dye, which was mixed with the diluted samples briefly before loading to give a 2.5% solution. Gels were pre-run in TB buffer or TBE buffer at electric fields of 100 V at 4 °C and 50 V at 25 °C for 5 min before they were run at 120 V at 4 °C for 3 h and 300 V at 25 °C for 25 min, respectively. Strand (dA)_35_ was covalently labelled with Cy5 dye for monitoring. Electrophoresis gels were imaged using a ChemiDoc MP multi-channel imager (Bio-Rad). Please note that TBE gels were run for 30 min at electric fields of 300 V at room temperature before loading.

## Online content

Any methods, additional references, Nature Portfolio reporting summaries, source data, extended data, supplementary information, acknowledgements, peer review information; details of author contributions and competing interests; and statements of data and code availability are available at 10.1038/s41557-024-01570-5.

### Supplementary information

Supplementary Information Supplementary Materials, Synthesis description, Notes, Kinetic model, Tables 1–13 and Figs. 1–21.

Supplementary Data 1 Source data of for Supplementary Figs. 1–7 and 19–21.

### Source data

Source Data Fig. 3 Source data and unprocessed gel.

Source Data Fig. 4 Source data.

Source Data Fig. 5 Source data.

Source Data Fig. 6 Source data.

Source Data Extended Data Fig. 2 Source data.

Source Data Extended Data Fig. 3 Source data and unprocessed gel.

Source Data Extended Data Fig. 4 Source data.

Source Data Extended Data Fig. 5 Source data.

Source Data Extended Data Fig. 6 Source data.

Source Data Extended Data Fig. 7 Source data.

Source Data Extended Data Fig. 8 Source data.

Source Data Extended Data Fig. 9 Source data.

Source Data Extended Data Fig. 10 Source data.

## Data Availability

All data that support the findings of this study are available within the paper and its Supplementary Information files. Raw data are available via figshare at 10.6084/m9.figshare.25550047 (ref. ^71^). Should raw data files be needed in another format, they are available from the corresponding author upon reasonable request. Source data are provided with this paper.

## Code availability

The kinetic model can be downloaded from GitHub at https://github.com/gerland-group/ChemicallyFueledOligomers.
