# Supplementary material for: Template-based copying in chemically fuelled dynamic combinatorial libraries
Source: Nat Chem. 2024 Jul 16;16(8):1240–9. doi: 10.1038/s41557-024-01570-5 (PMC11321992; doi:10.1038/s41557-024-01570-5)
Supplement: Supplementary file 1 — Supplementary Materials, Synthesis description, Notes, Kinetic model, Tables 1–13 and Figs. 1–21. [file 41557_2024_1570_MOESM1_ESM.pdf]

nature chemistry

Supplementary information https://doi.org/10.1038/s41557-024-01570-5

Template-based copying in chemically fuelled dynamic combinatorial libraries

In the format provided by the authors and unedited

Table of content

Materials

Synthesis

Supporting Notes

pH measurements.
Analysis of the impact of hydrolysis during HPLC analysis.
Oligonucleotide recovery.
Calculating the backbone length per repetition unit.
Calculation of oligomer frequencies.
Coacervate droplet experiments.
Hydrogel experiments.
Fluorescent labeling of pA.
Poly(vinyl)alcohol (PVA) coating procedure.
Partitioning of Me, T, and C in coacervate droplets made of pA and Ac-F(RG)$_3$N-NH$_2$.

Kinetic Model

Supporting Tables

Supporting Figures

References

Materials.

Ammonium persulfate, alpha-cyano-4-hydroxycinnamic acid, anhydrous acetic anhydride (Ac_2O), acetonitrile (ACN), ammonium formate, benzylamine (99%), (1-[bis(dimethylamino)methylene]-1H-1,2,3-triazolo[4,5-b]pyridinium 3-oxide hexafluorophosphate (HATU), cytosine, cytosine-1-acetic acid ethyl ester, 2'-deoxyhymidine 5'-monophosphate disodium salt, deoxyribonucleotide (dTMP), dextran sulfate sodium salt from Leuconostoc spp.,Mr ~20,000 (pDexS), 5-dimethyl-amino-isophthalic acid, *N*,*N*'-Diisopropylcarbodiimide (DIC), dichloromethane (DCM), 4-(dimethylamino)pyridine (DMAP), dimethylformamide (DMF), dimethyl sulfoxide (DMSO), deuterated dimethyl sulfoxide (DMSO-d6), ethanol (HPLC grade), ethyl acetate (EtOAc), 1-ethyl-3-(3-dimethylaminopropyl)carbodiimide hydrochloride (EDC), Ficoll 400, 1-hydroxybenzotriazole hydrate (HOBt), hydrochloric acid (HCl, 37%), hydroquinone, lithium hydroxide monohydrate ($LiOH \times H_2O$), magnesium chloride ($MgCl_2$), methyl iodide (CH_3I), 4-morpholinethane sulfonic acid (MES) buffer, *N*,*N*-Diisopropylethylamine (DIPEA), nile red, 5-nitro-isophthalic acid, *O*-(benzotriazol-1-yl)-*N,N,N,N',N'*-tetramethyluronium hexaflurophosphate (HBTU), palladium on carbon 10 wt.%, poly adenylic acid potassium salt (pA 100-500 kDa), polyuridylic acid potassium salt (pU, 600-1000kDa), potassium carbonate (K_2CO_3), polyvinylacohol M_w ~ 145 000 (PVA), anhydrous pyridine, 3-pyridylacetic acid hydrochloride, sodium acetate, sodium bicarbonate ($NaHCO_3$), sodium chloride, sodium hydroxide pellets, sodium sulfate (Na_2SO_4), tetramethylethylendiamin (TEMED), tert-butanol (*t*BuOH, 99%), tetrahydrofuran (THF, hplc grade), trifluoroacetic acid (TFA), 3-(Trimethylsilyl)-1-propanesulfonic acid sodium salt (TPS), triisopropylsilane (TIPS), tris-borate-EDTA buffer (TBE), tris hydrochloride (TRIS), thymine acetic acid, and triethylammonium acetate buffer (1 M) were purchased from Sigma-Aldrich. 2-(2,4-Dioxo-3,4-dihydropyrimidin-1(2H)-yl)acetic acid was purchased from abcr. DNA oligomers $(dA)_4$, $(dA)_{10}$, $(dAG)_{10}$ (AAAAAGGGGG), $(dG)_{10}$, $(dC)_{10}$, $(dT)_{10}$, $(dA)_{35}$, $(dA)_{35}$-Cy5, and $(dA)_{17}(dG)_{13}$ (AGA GAG AAA AAA AAA AAG GGG GGG GGG AAA) were purchased from Baseclick and Sigma-Aldrich. DNA oligomer Cy5-$(dU)_{15}$ was purchased from biomers.net GmbH. Cy3-Dextran sulfate, MW 40kDa, was purchased from CD Bioparticles. Acrylamide, Bisacrylamide were purchased from BioRad, Germany. High-performance liquid chromatography (HPLC) grade acetonitrile was purchased from VWR. All chemicals were used without any further purification unless otherwise indicated. Peptide Ac-F$(RG)_3$N-NH_2 was purchased from CASLO Aps (Denmark).

Synthesis

Synthesis of T.

Scheme S1. *Synthesis of **T**.*

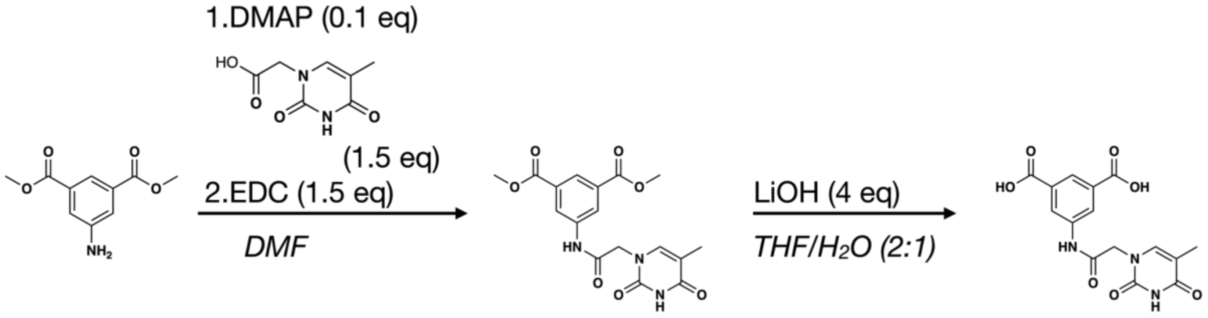

Synthesis of T-ester. Dimethyl-5-amino isophthalate (1 g, 4.7 mmol) was solubilized in dry DMF (20 mL). DMAP (0.06 g, 0.47 mmol, 0.1 eq) and thymine acetic acid (1.321 g, 7.17 mmol, 1.5 eq) were added under continuously stirring. After 15 min, the reaction solution was cooled to 0 °C. EDC (1.38 g, 7.17 mmol, 1.5 eq) was added portion-wise at 0 °C, and the reaction solution was stirred at 0 °C for 30 min, then for 16 h at room temperature. The reaction solution was shaken with ethyl acetate (20 mL). Subsequently, the organic layer was washed with 0.1 M aqueous HCL (3 × 20 mL), 10 % aqueous $NaHCO_3$ (3 × 10 mL), and brine (3 × 10 mL) and dried over Na_2SO_4. After evaporation of the solvent *in vacuo*, the residue gives **T-ester** as an orangish solid (1.62 g, 95 %), which was used in the next step without purification. An analytical sample was purified by preparative reversed-phase HPLC using a linear gradient of water/acetonitrile (each with 0.1% TFA).

T-ester: white powder (yield 1.62 g, 95 %), ^{1}H-NMR (400 MHz, DMSO-d_6): ∂(ppm) = 11.37 (s, 1H, NH), 10.74 (s, 1H, NH), 8.48 (d, J = 1.6 Hz, 2H, Ar-H), 8.19 (d, J = 1.9 Hz, 1H, Ar-H), 7.53 (d, J = 1.4 Hz, 1H, Ar-H), 4.54 (s, 2H, CH_2), 3.90 (s, 6H, CH_3), 1.79 (s, 3H, CH_3).^{13}C-NMR (101 MHz, DMSO-d_6): ∂(ppm) = 166.54, 165.16, 164.43, 151.12, 142.24, 139.51, 130.83, 124.29, 123.51, 108.18, 52.59, 11.93 (Fig. S7). LRMS (LC-MS): m/z_{calc} for $C_{17}H_{17}N_3O_7$: 375.11; found: $[M-H]^-$ = 374.01; HPLC: R_t = 15.2 min (HPLC-method 4, Fig. S6a).

Synthesis of T. **T-ester** (1.0 g, 2.6 mmol) was dissolved in 20 mL THF. The reaction solution was cooled to 0 °C. Subsequently, a solution of LiOH × H_2O (0.45 g, 10.6 mmol, 4 eq) in 10 mL of water was added dropwise at 0 °C. The reaction solution was stirred at 0 °C for 30 min, then stirred at room temperature for 12 h. After evaporation of the solvent *in vacuo*, the aqueous residue was acidified using 1 M HCl to neutral pH. The aqueous solution was purified by preparative reversed-phase HPLC using a linear gradient of water/acetonitrile (each with 0.1% TFA).

T: white powder (yield 498 mg, 55.4%), ^{1}H-NMR (400 MHz, DMSO-d_6): ∂(ppm) = 13.29 (s, 2H, COOH), 11.37 (s, 1H, NH), 10.72 (s, 1H, NH), 8.43 (d, *J* = 1.6 Hz, 2H, Ar-H), 8.18 (t, *J* = 1.5 Hz, 1H, Ar-H), 7.53 (d, *J* = 1.4 Hz, 1H, Ar-H), 4.55 (s, 2H, CH_2), 1.79 (d, *J* = 1.2 Hz, 3H, CH_3). ^{13}C-NMR (101 MHz, DMSO-d_6): ∂(ppm) = 166.87, 164.91, 151.61, 142.77, 139.66, 132.45, 125.29, 123.96, 108.61, 50.51, 12.3 (Fig. S8). LRMS (LC-MS): m/z_{calc} for $C_{15}H_{13}N_3O_7$: 347.08; found: $[M-H]^-$ = 346.21; HPLC: R_t = 8.8 min (HPLC method 4), 5.3 min (HPLC method 4, Fig. S6b).

Synthesis of Me-T.

Scheme S2. *Synthesis of* ***Me-T****.*

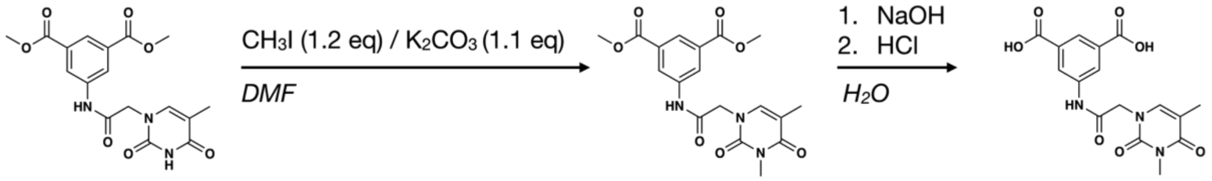

T-ester (300 mg, 0.8 mmol) was dissolved in 2 mL of DMF. K_2CO_3 (122 mg, 0.88 mmol, 1.1 eq.) was added under continuous stirring. After 30 minutes of stirring under argon at room temperature, CH_3I (136 mg, 60 µL, 1.2 eq.) was added dropwise. The reaction solution was stirred overnight at room temperature. Monitoring by LC-MS confirmed the complete conversion of the starting material to the **Me-T ester**. DMF was removed under a vacuum. The resulting oil was suspended in water to hydrolyze **Me-T ester** to **Me-T**. Concentrated NaOH was added dropwise while stirring until a transparent solution was obtained (pH ~ 12). After one hour of stirring, LC-MS confirmed the complete conversion of the **Me-T ester** to **Me-T**. **Me-T** precipitated through acidification of the reaction solution with concentrated HCl to pH ~ 1. The white precipitate was filtered off and dried to give pure **Me-T** (yield 0.201 g, 70%).

Me-T: white powder (yield 0.201 g, 70%), ^{1}H NMR (300 MHz, DMSO) δ 13.27 (s, 2H, COOH), 10.66 (s, 1H, NH), 8.41 (s, 2H, Ar-H), 8.18 (s, 1H, Ar-H), 7.59 (s, 1H, Ar-H), 4.60 (s, 2H, CH_2), 3.18 (s, 3H, CH_3), 1.84 (s, 3H, CH_3). ^{13}C NMR (75 MHz, DMSO) δ 166.33, 166.27, 163.41, 151.29, 140.74, 139.15, 131.87, 124.85, 123.53, 107.24, 51.17, 27.49, 12.58 (Fig. S9). LRMS (LC-MS): m/z_{calc} for $C_{16}H_{15}N_3O_7$: 361.09; found: $[M-H]^-$ = 360.81; HPLC: R_t = 10.5 min (HPLC method 1, Fig. S6c).

Synthesis Me.

Scheme S3. *Synthesis of* ***Me****.*

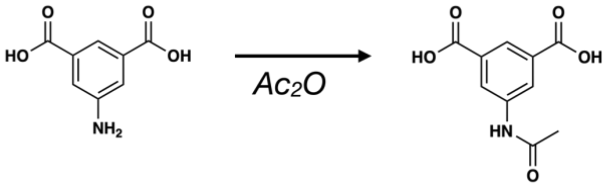

Dimethyl-5-amino isophthalate (194 mg, 1.07 mmol) was solubilized in anhydrous acetic anhydride (5 mL) under continuous stirring. The reaction solution was heated to 100°C for one hour and cooled to 0 °C. The product oil phase was extracted with ethyl acetate and dried over Na_2SO_4. Evaporation of the solvent in vacuo gave pure **Me**.

Me: white powder (yield 217 mg, 90.8%), ^{1}H-NMR (400 MHz, DMSO-d_6): ∂(ppm) = 13.23 (s, 2H, COOH), 10.30 (s, 1H, NH), 8.42 (d, J = 1.6 Hz, 2H, Ar-H), 8.14 (t, J = 1.6 Hz, 1H, Ar-H), 2.07 (s, 3H, CH_3). ^{13}C-NMR (101 MHz, DMSO-d_6): ∂(ppm) = 169.29, 166.96, 140.37, 132.15, 124.82, 123.82, 24.49 (Fig. S10). LRMS (LC-MS): m/z_{calc} for $C_{10}H_9NO_5$: 223.05; found: $[M-H]^-$ = 222.04; HPLC: R_t = 8.5 min (HPLC method 1), 5.4 min (HPLC method 3, Fig. S6d).

Synthesis C.

Scheme S4. *Synthesis of (Boc)$_2$-5-aminoisophthalate.*

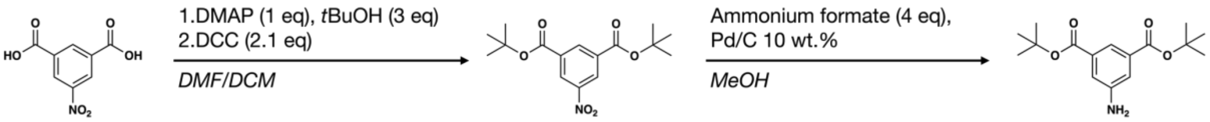

Synthesis of (Boc)$_2$-5-nitroisophthalate. 5-nitro isophthalate (6.33 g, 30 mmol), DMAP (1.226 g, 10 mmol, 1 eq), and tert-butanol (8.6 mL, 90 mmol, 3 eq) were solubilized in dry DMF (80 mL) and dry DCM (80 mL) using a modified literature procedure.[1] The reaction solution was cooled to 0°C for ten minutes before adding DCC (13.61 g, 66 mmol, 2.1 eq). The reaction solution was warmed up to room temperature and stirred overnight. DCU was filtered off. The reaction solution was washed with brine and dried over Na_2SO_4. After evaporation of the solvent in vacuo, the crude was recrystallized from methanol to give pure (Boc)$_2$-5-nitroisophthalate.

(Boc)$_2$-5-nitroisophthalate: white powder (yield 4.6 g, 47.8%), ^{1}H-NMR (400 MHz, DMSO-d_6): ∂(ppm) = 8.73 (d, J = 1.5 Hz, 2H, Ar-H), 8.67 (t, J = 1.6 Hz, 1H, Ar-H), 1.60 (s, 18H, CH_3). ^{13}C-NMR (101 MHz, DMSO-d_6): ∂(ppm) = 162.14, 148.00, 134.25, 133.09, 126.90, 82.69, 39.94,

39.73, 39.64, 39.52, 39.31, 39.10, 38.90, 38.69, 27.38 (Fig. S11). R_t = 14.6 min (HPLC method 3, Fig. S6e).

Synthesis of (Boc)$_2$-5-aminoisophthalate. (Boc)$_2$-5-nitroisophthalate (1 g, 3.1 mmol) was solubilized in 30 mL dry methanol under argon using a modified literature procedure.[1] 100 mg palladium on carbon 10 wt.% was added, and then ammonium formate (0.78 g, 12.4 mmol, 4 eq). The reaction solution was stirred for 30 minutes before filtering off palladium. The filtrate was diluted with DCM, washed with brine, and dried over Na_2SO_4 to give pure crude.

(Boc)$_2$-5-aminoisophthalate: white powder (yield 4.26 g, 100%), ^{1}H-NMR (400 MHz, DMSO-d$_6$): ∂(ppm) = 7.55 (t, J = 1.5 Hz, 1H, Ar-H), 7.31 (d, J = 1.6 Hz, 2H, Ar-H), 5.64 (s, 2H, NH_2), 1.53 (s, 18H, CH_3). ^{13}C-NMR (101 MHz, DMSO-d$_6$): ∂(ppm) = 164.79, 149.23, 132.16, 117.73, 116.74, 80.63, 40.15, 39.94, 39.73, 39.52, 39.31, 39.10, 38.90, 27.74 (Fig. S12). LRMS (LC-MS): m/z_{calc} for $C_{16}H_{23}NO_4$: 293.16; found: $[M-H]^+$ = 294.02; HPLC: R_t = 11.6 min (HPLC method 3, Fig. S6f).

Scheme S5. *Synthesis of* ***C****.*

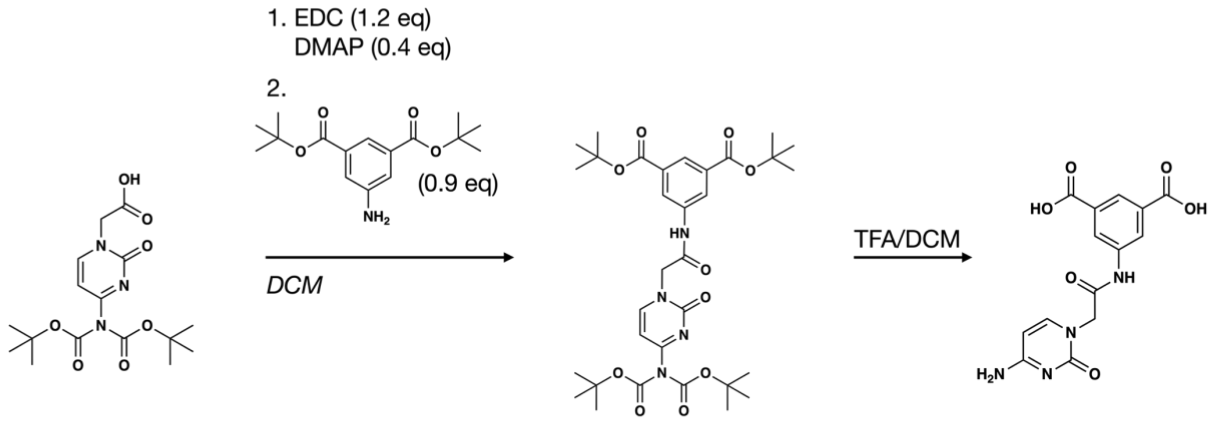

Synthesis of C. (Boc)$_2$-cytosine-1-acetic acid was synthesized according to literature from cytosine-1-acetic acid ethyl ester.[2] (Boc)$_2$-cytosine-1-acetic acid (150 mg, 0.41 mmol) was dissolved in dry DCM (2mL) followed by DMAP (20 mg, 0.4 mmol, 0.4 eq) and EDC (93.4 mg, 0.49 mmol, 1.2 eq) under argon. The reaction solution was stirred for five minutes under argon before (Boc)$_2$-5-aminoisophthalate (107 mg, 0.366 mmol, 0.9 eq) was added. The reaction progress was monitored by LC-MS. After the complete conversion of the starting materials, the reaction mixture was diluted with DCM (20 mL). The organic layer was washed with 1 M $KHSO_4$ (2 × 20 mL), saturated $NaHCO_3$ (2 × 20 mL), and MQ water (1 × 20 mL) and dried over anhydrous Na_2SO_4. After evaporating the solvent in vacuo, the crude was treated with 10 mL of 80% TFA in DCM for one hour under argon to remove the Boc-protecting groups. The concentration of the reaction solution and subsequent dilution with cold diethyl ether induced

precipitation of the final product. The yield was 95 mg (70%). The crude was purified by preparative reversed-phase HPLC using a linear gradient of water/acetonitrile (each with 0.1% TFA).

C: white powder (yield 95 g, 70%), ^{1}H-NMR (400 MHz, DMSO-d_6): ∂(ppm) = 10.78 (s, 2H, COOH), 9.24 (s, 1H, NH), 8.4 (d, J = 1.5 Hz, 3H, Ar-H), 8.18 (t, J = 1.6 Hz, 1H, Ar-H), 7.93 (d, J = 7.5 Hz, 1H, Ar-H), 4.69 (s, 2H, CH_2).^{13}C-NMR (101 MHz, DMSO-d_6): ∂(ppm) = 166.37, 165.72, 161.10, 158.36, 150.03, 149.59, 139.10, 131.98, 124.99, 123.56, 93.32, 51.33, 1.19 (Fig. S13). LRMS (LC-MS): m/z_{calc} for $C_{14}H_{12}N_4O_6$: 332.08; found: $[M-H]^-$ = 331.13; HPLC: R_t = 5.9 min (HPLC method 1, Fig. S6g).

Synthesis of Me-C

Scheme S6. *Synthesis of N,N-dimethylcytosine-1-acetic acid.*

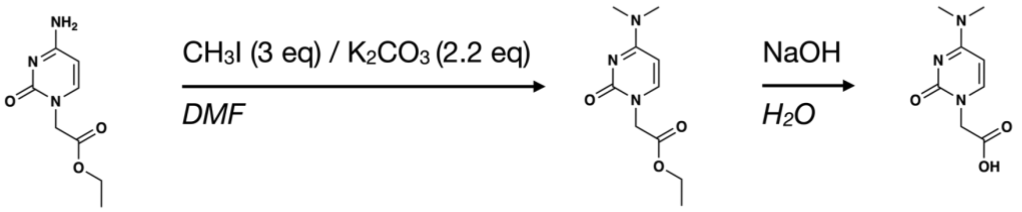

Synthesis of N,N-dimethylcytosine-1-acetic acid ethyl ester. Cytosine-1-acetic acid ethyl ester (200 mg, 1 mmol) was methylated with methyl iodide (190 µL, 3.04 mmol, 3 eq.) in the presence of K_2CO_3 (308 mg, 2.23 mmol, 2.2 eq.) in DMF at room temperature for three days. MeI was added in two steps. First, 2 eq. of MeI were added in one portion, followed by another 1 eq. of MeI after 24 hours. Reaction progress was monitored by LC-MS, confirming the formation of N,N-dimethylcytosine-1-acetic acid ethyl ester. The solvent and unreacted MeI were removed under a vacuum to give the crude. The crude was directly used in the next step without further purification.

Synthesis of N,N-dimethylcytosine-1-acetic acid. Ethyl ester protection was removed by suspending N,N-dimethylcytosine-1-acetic acid ethyl ester in water and adding dropwise concentrated NaOH while stirring until a clear solution was obtained (pH ~ 12). After 30 minutes of stirring, LC-MS confirmed the complete deprotection of N,N-dimethylcytosine-1-acetic acid ethyl ester. The mixture was neutralized with concentrated HCl. Evaporation of water gives a solid, which was re-suspended in DCM/MeOH (90/10 (v/v)). The precipitate was filtered off and dried to give N,N-dimethylcytosine-1-acetic acid ethyl acid (yield 152 mg, 60%). The crude was used in the following steps without further purification. An analytical sample of the compound was purified preparative reversed-phase HPLC using a linear gradient of water/acetonitrile (each with 0.1% TFA).

N,N-dimethylcytosine-1-acetic acid ethyl acid white powder (yield 152 mg), ^{1}H-NMR (300 MHz, DMSO-d_6): ∂(ppm) = 13.54 (1H, COOH), 9.53 (s, 1H, NH if relocated), 8.16 (d, J = 7.9 Hz, 1H, Ar-H), 6.51 (d, J = 8.0 Hz, 1H, Ar-H), 4.64 (s, 2H, CH_2), 3.42 (s, 3H, CH_3), 3.07 (s, 3H, CH_3). ^{13}C-NMR (75 MHz, DMSO-d_6): ∂(ppm) = 168.54, 157.92, 148.18, 148.11, 91.06, 50.80, 30.84, 30.56. (Fig. S14). LRMS (LC-MS): m/z_{calc} for $C_8H_{11}N_3O_3$: 197.08; found: $[M+H]^+$ = 198.05.

Scheme S7. *Synthesis of* ***Me-C****.*

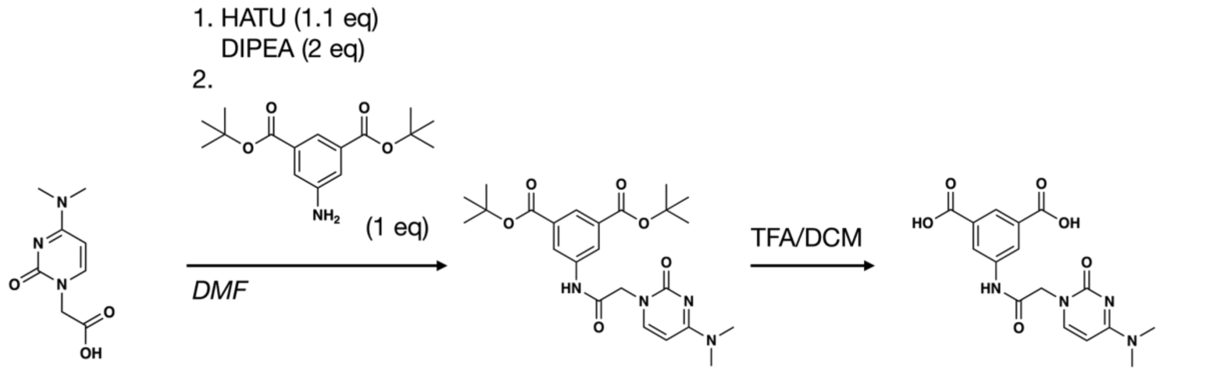

Synthesis of Me-C ester. N,N-dimethylcytosine-1-acetic acid (150 mg, 0.76 mmol) was dissolved in 3 mL of DMF. HATU (318 mg, 0,836 mmol, 1.1 eq.) and DIPEA (265 µL, 1.52 mmol, 2 eq.) were added under an argon atmosphere while stirring to activate the acid. After five minutes, $(Boc)_2$-5-nitroisophthalate (225 mg, 0.761 mmol, 1 eq.) was added, and the reaction solution was stirred for three hours. The reaction progress was monitored by LC-MS, confirming the formation of the **Me-C ester**. The **Me-C ester** was precipitated by pouring the reaction solution into water. The white precipitate was centrifugated and dried under a vacuum.

Synthesis of Me-C. Boc protection was removed by stirring crude **Me-C ester** in a 10 mL TFA/DCM/H_2O/TIPS (80/15/2.5/2.5 (v/v/v)) cocktail for one hour. The reaction solution was concentrated to half the initial volume and poured into 45 mL of cold diethyl ether leading to precipitate **Me-C**. The crude was purified by preparative reversed-phase HPLC using a linear gradient of water/acetonitrile (each with 0.1% TFA).

Me-C: white powder (yield 136 mg, 50%), ^{1}H-NMR (400 MHz, DMSO-d_6): ∂(ppm) = 13.34 (s, 2H, COOH), 10.86 (s, 1H, NH), 9.47 (s, 1H, Ar-H), 8.41 (d, J = 1.6 Hz, 2H, Ar-H), 8.33 – 8.06 (m, 2H, Ar-H), 6.55 (d, J = 8.0 Hz, 1H, Ar-H), 4.81 (s, 2H, CH_2), 3.44 (s, 3H, CH_3), 3.10 (s, 3H, CH_3). ^{13}C-NMR (101 MHz, DMSO-d_6): ∂(ppm) = 166.28, 165.13, 157.95, 148.75, 148.31, 138.95, 131.98, 123.55, 90.83, 52.44, 30.87, 30.62 (Fig. S15). LRMS (LC-MS): m/z_{calc} for $C_{16}H_{16}N_4O_6$: 360.11; found: $[M-H]^-$ = 361.26; HPLC: R_t = 6.2 min (HPLC method 1, Fig. S6h).

Synthesis of 3-pyridyl IPA.

Scheme S8. *Synthesis of 3-pyridyl IPA.*

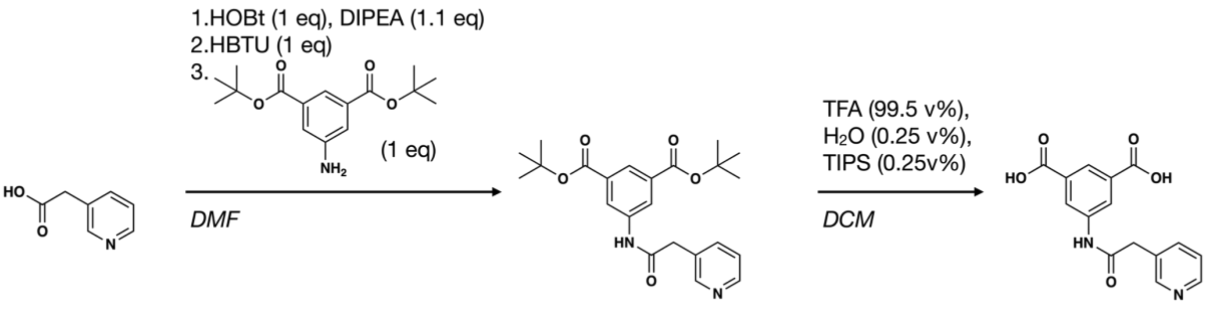

Synthesis of 3-pyridyl IPA ester. 3-pyridyl IPA (102.84 mg, 0.75 mmol), DIPEA (88.11 mg, 0.68 mmol, 1.1 eq), HOBt (92.12 mg, 0.67 mmol, 1 eq) were solubilized in dry DMF (3 mL). After 10 min HBTU (284.41 mg, 0.75 mmol, 1 eq) was added and stirred for 30 min before adding $(Boc)_2$-5-aminoisophthalate (200 mg, 0.68 mmol, 1 eq). After the reaction solution was stirred overnight, the reaction solution was slowly dropped into MQ water. The precipitate was filtered off, washed with water, and then lyophilized to give crude $(Boc)_2$-3-pyridyl IPA.

Synthesis of 3-pyridyl IPA. Boc protection was removed by stirring crude $(Boc)_2$-3-pyridyl IPA in a TFA/H_2O/TIPS (99.5/0.25/0.25 (v/v/v)) cocktail for two hours. TFA/H_2O/TIPS cocktail was removed by evaporation in vacuo. The crude was purified by preparative reversed-phase HPLC using a linear gradient of water/acetonitrile (each with 0.1% TFA).

3-pyridyl IPA: yellowish powder (yield 118 mg, 52.3%), ^{1}H-NMR (400 MHz, DMSO-d_6): ∂(ppm) = 13.17 (s, 2H, COOH), 10.73 (s, 1H, NH), 8.83 – 8.60 (m, 2H, Ar-H), 8.44 (d, J = 1.6 Hz, 2H, Ar-H), 8.32 – 8.19 (m, 1H, Ar-H), 8.16 (t, J = 1.6 Hz, 1H, Ar-H), 7.74 (dd, J = 8.0, 5.3 Hz, 1H, Ar-H), 3.89 (s, 2H, CH_2). ^{13}C-NMR (101 MHz, DMSO-d_6): ∂(ppm) = 168.38, 166.41, 158.58, 158.24, 145.79, 143.55, 142.97, 139.58, 133.69, 131.83, 125.30, 124.74, 123.59, 40.14 (Fig. S16). LRMS (LC-MS): m/z_{calc} for $C_{15}H_{12}N_2O_5$: 300.07; found: $[M-H]^-$ = 299.76; HPLC: R_t = 6.3 (HPLC method 1, Fig. S6i).

Synthesis of U.

Scheme S9. *Synthesis of U.*

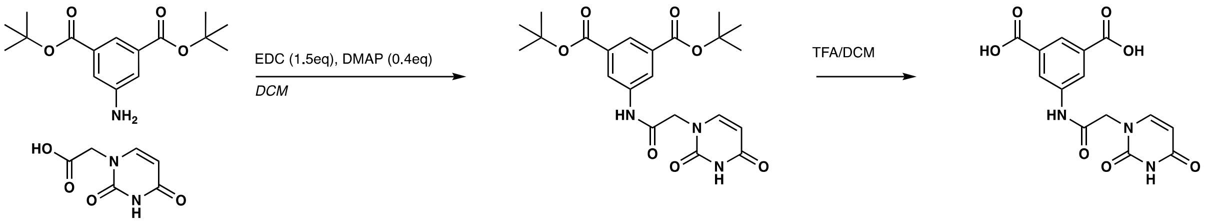

Synthesis of U-ester. 2-(2,4-Dioxo-3,4-dihydropyrimidin-1(2H)-yl)acetic acid (150 mg, 0.881 mmol), DMAP (43 mg, 0.352 mmol, 0.4 eq.) were dispersed in dry DCM (5 mL). After that EDC (253.5 mg, 1.32 mmol, 1.5 eq) was added, and the reaction mixture was stirred for 10 min, before adding $(Boc)_2$-5-aminoisophthalate (258,7 mg, 0.881 mmol, 1 eq). The resulting suspension was stirred overnight, leading to the dissolution of all the reagents. Reaction completion was monitored by LC-MS, showing the disappearance of the starting material and emergence of the peak of the product, the boc-protected **U**. The reaction was poured into the separation funnel with 30 mL of DCM and was washed twice with the same volume of 1M $KHSO_4$ solution and once with water. The solvent was evaporated on the rotary evaporator, leading to the crude (Boc)2-U (326 mg, 83%), which was then deprotected as described below.

Synthesis of U. Boc protection was removed by stirring crude $(Boc)_2$-U in 10 mL TFA/H_2O/TIPS (99.5/0.25/0.25 (v/v/v)) cocktail for two hours. The volume of TFA/H_2O/TIPS cocktail was then reduced in vacuo, and the concentrated solution was poured into 30 mL diethyl ether with the emergence of a white precipitate. The precipitate was filtered and purified by preparative reversed-phase HPLC using a linear gradient of water/acetonitrile (each with 0.1% TFA). The sample of the crude and purified product was analyzed by LC-MS, confirming the identity of **U**.

U: white powder (yield 161 mg, 66 %), ^{1}H-NMR (400 MHz, DMSO-d_6): ∂(ppm) = 13.27 (s, 2H, COOH), 11.35 (d, J = 2.3 Hz, 1H, NH), 10.66 (s, 1H, NH), 8.41 (d, J = 1.6 Hz, 2H, Ar-H), 8.18 (t, J = 1.5 Hz, 1H, Ar-H), 7.65 (d, J = 7.8 Hz, 1H, Ar-H), 5.62 (dd, J = 7.8 Hz,1H, Ar-H), 4.57 (s, 2H, CH_2). ^{13}C-NMR (101 MHz, DMSO-d_6): ∂(ppm) = 166.13, 166.07, 163.66, 150.94, 146.33, 138.97, 131.69, 124.64, 123.30, 100.51, 50.04. (Fig. S17). LRMS (LC-MS): m/z_{calc} for $C_{15}H_{12}N_2O_5$: 333.06; found: $[M-H]^-$ = 332.05; HPLC: R_t = 7.5 (HPLC method 1, Fig. S6j).

Supplementary Notes

pH measurements. pH changes before and after the addition of EDC for fueled kinetic experiments (**T** at 25 mM, 10 mM pyridine or **C** at 25 mM, 10 mM pyridine) were performed in 0.5 mL Eppendorf tubes on a volume of 100 µL using an HI 2210 pH meter from HANNA® Instruments. The pH remained stable between 6.0 to 6.04, and 6.5 to 6.51 (Fig. S18).

Analysis of the impact of hydrolysis during HPLC analysis. We used a quenching method described in previous work[3] to determine the effect of hydrolysis during HPLC analysis. Only minimal amounts of the anhydrides formed hydrolyzed (Fig. S19). We calculated the impact of hydrolysis by comparing the total anhydride concentration with and without quenching, as described previously.[4] We used the typical reaction conditions of 25 mM **T**, **U**, or **C** and 10 mM pyridine dissolved in 200 mM MES buffered water at pH 6 for **T**, **U**, or 6.5 for **C**, fueled with 10 mM EDC (total volume: 100 µL). At three time points during the reaction cycle, we took an aliquot of 5 µL and quenched it with 15 µL of 400 mM benzylamine, yielding a final concentration of 300 mM benzylamine. The resulting transparent solution (pH > 9) was subsequently measured *via* HPLC to determine the concentration of monobenzylamide **BA T**, **BA U**, and **BA C**. Bisbenzylamide **BA 2T**, and **BA 2C**, were not observed by mass spectrometry, whereas **BA 2U** was. 1 µL of the quenched solution was directly injected into the column without further dilution, and all compounds involved were separated using a linear gradient of ACN (2% to 98%) and MQ-water, each with 0.1% TFA, with a flow of 1 $mLmin^{-1}$ and UV-Vis detection at 290 nm (HPLC method 1). In the case of the non-quench experiments, 1 µL of the reaction solution was directly injected into the HPLC column.

Oligonucleotide recovery. We confirmed that templates $(dA)_4$ and $(dA)_{10}$ do not react with EDC, pyridine, **T**, or oligomers and do not degrade during the reaction cycle by recovering the oligomer after the reaction cycle. We fueled **T** at 25 mM and 10 mM pyridine in 200 mM MES buffered water at pH 6 with 500 mM EDC, in a total volume of 100 µL. After one day, we recovered template $(dA)_4$ or $(dA)_{10}$ by precipitation with ethanol according to the Cold Spring Habor protocol.[5] We added a 3 M sodium acetate solution to give a final concentration of 300 mM in a 100 µL reaction solution. Subsequently, we added 900 µL of ice-cold EtOH. The solution was kept in the fridge at 5 °C overnight, then for four hours in the freezer at -20 °C. The solution was centrifuged at 12300 xg for ten min with a MicroStar12 (VWR) before removing the supernatant. The residue was solubilized in 100 µL MQ water. The purity was checked by analytical reversed-phase HPLC on a DNA oligomer column. We compared the recovered DNA oligomer $(dA)_4$ with a fresh DNA oligomer $(dA)_4$ at 4 µM. Both samples looked identical (Fig. S20).

Calculating the backbone length per repetition unit. We calculated the total bond length of the repetition unit by adding up the single bond lengths. We measured the individual bond lengths on a dimer structure after optimization with the software Avogadro (Table S1).

Calculation of oligomer frequencies. For all observed oligomer lengths, we calculated the frequency of **T** and **C** as described below for the dimer.

Step 1

	T_2	TC	C_2	$[T]_{total}/[C]_{total}$
[T] (mM)	$2 \times [T_2]$	$1 \times [TC]$	$0 \times [C_2]$	$\Sigma([T])$
[C] (mM)	$0 \times [T_2]$	$1 \times [TC]$	$2 \times [C_2]$	$\Sigma([C])$

$c_{total} = \Sigma([T]_{total} + [C]_{total})$

Step 2

	T_2	TC	C_2	Frequency T_{total}/C_{total}
T	$(2 \times [T_2])/c_{total}$	$(1 \times [TC])/c_{total}$	$(0 \times [C_2])/c_{total}$	$\Sigma(T)$
C	$(0 \times [T_2])/c_{total}$	$(1 \times [TC])/c_{total}$	$(2 \times [C_2])/c_{total}$	$\Sigma(C)$

Coacervate droplet experiments. Complex coacervates form by mixing 0.86 mM pA (RNA, expressed in monomer concentration) with 5 mM polycation Ac-F(RG)$_3$N-NH$_2$ at pH 6 (phenylalanine (F), arginine (R), glycine (G), and asparagine (N), Fig. 4a, Extended Data Fig. 9a, b). Twenty minutes after starting the reaction cycle by adding fuel, we centrifuged the samples to separate coacervates and supernatant. We removed the supernatant and added buffer to fill up to a quarter of the initial volume to ensure that monomer concentrations remained high. We sonicated the sample and obtained a turbid suspension of coacervate droplets. Then we started the reaction cycle again by adding 50 mM EDC. After twenty minutes, we centrifuged the samples and separated the pellet and supernatant. We repeated the above-described procedure three times in total (Fig. 4b). After each cycle, we took an aliquot to analyze the composition of the coacervate phase by HPLC after 24 hours (Fig. 5c, d) to ensure that all oligomers had hydrolyzed to the respective monomers.

For the mixed libraries containing more than two precursors, we used coacervates formed between 0.6 mM (dA)$_{17}$(dG)$_{13}$ (expressed in monomer concentration) and 5 mM polycation Ac-F(RG)$_3$N-NH$_2$ at pH 6 (Fig. 5f, Extended Data Fig. 9c). For analysis of the library we used HPLC method 4.

Hydrogel experiments. We used the ability of pA (RNA) to form hydrogels when hybridized with **T**-oligomers.[6,7] We mixed a pool of 20 mM **T** and 20 mM **Me** monomers with 4.32 mM pA when expressed as monomer concentration. After applying 50 mM fuel, the clear solution formed a hydrogel (Fig. S5). Thirty minutes after starting the reaction cycle by adding fuel, we gently centrifuged the samples to separate the gel and supernatant. We removed the supernatant and added buffer to fill up to a quarter of the initial volume to ensure that monomer concentrations remained high. Noteworthy, we took an aliquot of the gel phase and waited 24 hours for the pellet to dissolve and all oligomers to deoligomerize before analyzing its composition by HPLC. After the first cycle, the hydrogel comprised equal amounts of **T** and **Me**. After the second cycle, the hydrogel comprised 70% **T** and 30% **Me**. The third cycle did not change the hydrogel composition any further. Noteworthy, droplets could purify the library effectively and more efficiently than hydrogels, likely because droplets are more easily separated from their supernatant than hydrogels.

Fluorescent labeling of pA. 41.3 mM pA (expressed as monomer concentration) was fluorescent-labeled by hybridization with 1.5 mM Cy5-$(dU)_{15}$ (expressed as monomer concentration) to give 8.6 mM fluorescent pA (expressed as monomer concentration). To guarantee efficient hybridization, pA and Cy5-$(dU)_{15}$ were annealed with a thermal cycler T100^{TM} (BioRad) following the protocol: heating from 25 °C in one minute, holding temperature for three minutes and cooling to 25 °C with 0.1 °Ch^{-1}. The fluorescent pA was stored at -20°C until further use. 0.086 mM (expressed as monomer concentration) of Cy5 labelled pA was freshly added to the confocal experiment as a dye.

Poly(vinyl)alcohol (PVA) coating procedure. We inhibited the wetting of coacervates by coating ibidi µ-slide angiogenesis well plates with a glass bottom with PVA using a modified literature procedure.[8] We washed the ibidi chambers three times with soap water, MQ water and dried them with nitrogen pressure. Then, we plasma-cleaned the slides for 3 minutes and added 50 µL of a freshly prepared 5% PVA solution in MQ water to each well. The slides were incubated for 15 minutes before we removed the PVA solution and washed the wells 3 times with MQ water, then dried with nitrogen pressure. The slides were placed in the oven for 1 hour at 80 °C for 30 minutes. The obtained coated wells were stored at room temperature and consumed within one week.

Partitioning of Me, T, and C in coacervate droplets made of pA and Ac-F$(RG)_3$N-NH_2. We determined the concentration of **Me**, **T**, and **C** inside the coacervates, by measuring their concentrations using HPLC. 1 µM pA was added to a solution containing 20 mM of **Me**, or **T** or **C** in 200 mM MES at pH 6 (**T**, **Me**) or 6.5 (**C**). The turbid suspension (V = 150 µL) was vortexed, incubated for 10 min, and then centrifuged for another 5 min at 12300 xg (MicroStar

12, VWR). We removed the supernatant and added it into an HPLC inlet. The coacervate pellet was dissolved by adding 20 µL of NaCl (4 M) aqueous solution. The resulting clear solution was injected into the HPLC and compared to the supernatant. To calculate the partitioning of **Me**, **T**, and **C** in the droplet phase, we estimated the total droplet volume, before dissolving with NaCl solution, by comparing it to size standards visually. We calculated the partition coefficient according to the following equation.[9]

$$P = \frac{C_{coacervate}}{C_{supernatant}}$$

Kinetic Model

The kinetic model can be downloaded from https://github.com/gerland-group/ChemicallyFueledOligomers

Kinetic model without template

We devised a kinetic model using Python3 to describe the concentrations of all relevant components of the fuel-driven dynamic chemical library as a function of time. The kinetic model calculates the relevant concentrations by integrating the chemical rate equations of the following five chemical reactions in solution numerically using the Runge-Kutta-Fehlberg method (RK45): the activation of monomer and oligomers, the ligation of activated monomers/oligomers with non-activated monomers/oligomers, the hydrolysis of activated monomer and activated oligomers, side product formation of N-acylisourea by a rearrangement reaction (N to O shift), the hydrolysis of the oligomers.

Reaction 0 ($k_{0, solution}$) – Hydration of EDC

The carbodiimide fuel EDC directly hydrates with a first-order rate constant of $k_{0, solution}$ = 0.01224 ± 0.002 (h^{-1}) as determined by NMR previously.[4] Noteworthy, we can consider the direct hydration of EDC as irrelevant since the timescale of hydration $\tau \sim 100$ h is long compared to experimental timescales of 1-3 h.

Scheme S10. *Reaction 0 ($k_{0, solution}$).*

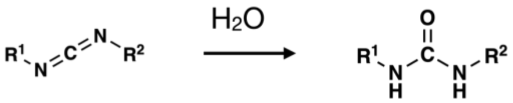

Reaction 1 ($k_{1, solution}$) – Activation of monomer and oligomers

Fuel activates monomers and oligomers to their corresponding activated state, the O-acylisourea in a second-order reaction.

Scheme S11. *Reaction 1 ($k_{1,\ solution}$).*

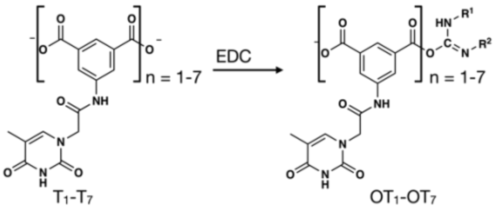

Reaction 2 ($k_{2,\ solution}$) – Ligation of activated monomer and activated oligomers

Activated monomers and activated oligomers can react with monomers and oligomers to form longer oligomers in a second-order reaction. The length of the oligomers formed by ligation equals the total length of both ingoing reactants. We considered a maximum oligomer length of l_{max} = 7.

Scheme S12. *Reaction 2 ($k_{2,\ solution}$).*

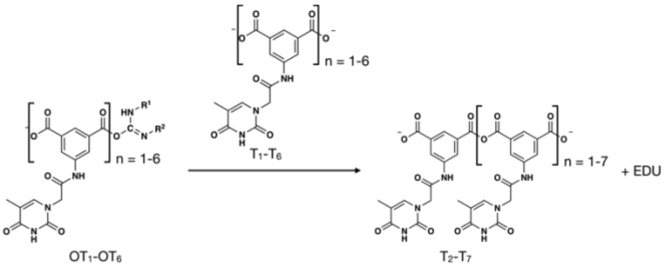

Reaction 3 ($k_{3,\ solution}$) – Hydrolysis of activated monomer and activated oligomers

Activated monomers and activated oligomers spontaneously hydrolyze in a pseudo-first-order reaction to monomer, oligomer and waste EDU.

Scheme S13. *Reaction 3 ($k_{3,\ solution}$).*

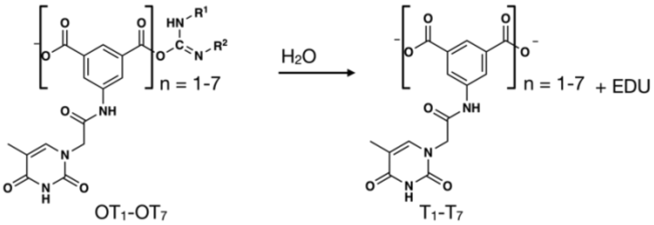

Reaction 4 ($k_{4,\ solution}$) – Formation of side product N-acylisourea through rearrangement O-to-N shift.

Activated monomers and activated oligomers rearrange in a first-order reaction to the unwanted side product N-acylisourea.

Scheme S14. *Reaction 4 ($k_{4,\ solution}$).*

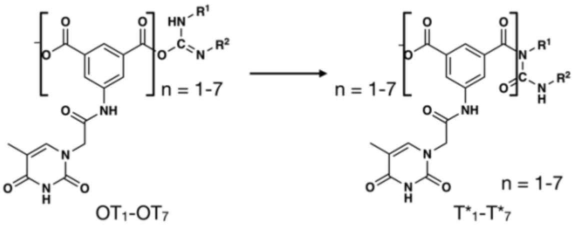

Reaction 5 ($k_{5,\ solution}$) – Hydrolysis of oligomers

Oligomers spontaneously hydrolyze in a pseudo-first-order reaction, yielding shorter oligomers and, finally, monomers. The length of the shorter oligomers equals in sum the length of the ingoing oligomer.

Scheme S15. *Reaction 5 ($k_{5,\ solution}$).*

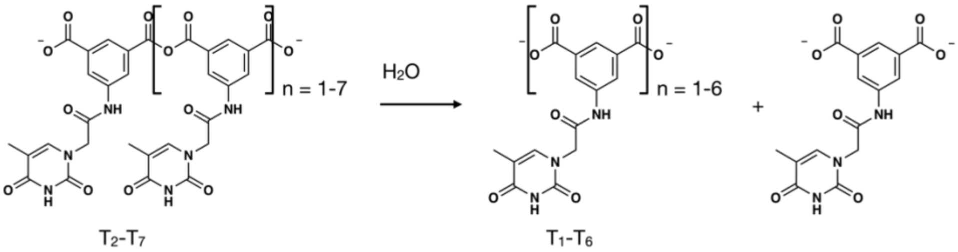

In the kinetic model, we neglected transacylation between oligomers. As transacylation is a second-order reaction and the concentrations of oligomers are low, the transacylation rate is expected to be small compared to the degradation of oligomers *via* anhydride hydrolysis. Moreover, we disregard the activation of unwanted side product N-acylisourea.

Computing the reaction rate constants by curve fitting

We used the method of least squares to determine the set of optimal parameters of reaction rate constants. We minimized the weighted sum of squared residuals (equation 1) since oligomer concentrations vary in order of magnitude depending on the oligomer length. Oligomer concentrations of **DynT$_4$** and **DynT$_5$** are two orders of magnitude smaller than **DynT$_2$**. Thus, the contributions of long oligomers to the sum of squared residuals are small, even for relatively bad fits. By minimizing the weighted sum of squared residuals (equation 1), we ensured that the curve fit is not dominated by finding the best fit for short oligomers without accounting for the quality of the curve fit of long oligomers.

$$\chi = \sum_{l=1}^{l_{max}} \sum_{g} \frac{\sum_{d=1}^{N_{l,g}} \left[c^{experiment}(l,g,t_d) - c^{theory}(l,g,t_d)\right]^2}{\left[\sum_{d=1}^{N_{l,g}} c^{experiment}(l,g,t_d)\right]^2}$$

Equation 1

Here, l is the length of the oligomer and g is the terminal group of the oligomer, *i.e.,* carboxyl group, O-Acylisourea group or N-Acylisourea group. $N_{l,g}$ is the number of experimentally measured data points, *i.e.,* oligomer concentrations of length l and terminal group g at different time points t_d. Due to the weight, the contributions of each type of oligomer to the sum of squared residuals are comparable, even though the concentrations differ significantly.

Curve fitting with length-independent reaction rate constants

We determined the five reaction rate constants with the above-described curve fitting procedure: $k_{1,\ solution}$, $k_{2,\ solution}$, $k_{3,\ solution}$, $k_{4,\ solution}$, $k_{5,\ solution}$ (Fig. S1, Table S5). We presumed that the rate constants are independent of oligomer length. Our length-independent rate constants underestimate experimentally determined maximum concentrations of **DynT$_4$** and **DynT$_5$**, 3-fold and 40-fold, respectively.

Curve fitting with length-dependent reaction rate constants

To recover the concentration profiles of **DynT$_4$** and **DynT$_5$**, we introduced five distinct length-dependent activation rate constants $k_{1,\ solution}$ (l = 1), $k_{1,\ solution}$ (l = 2), $k_{1,\ solution}$ (l = 3), $k_{1,\ solution}$ (l = 4), $k_{1,\ solution}$ (l ≥ 5) and five distinct independent anhydride hydrolysis rate constants $k_{5,\ solution}$ (l = 2), $k_{5,\ solution}$ (l = 3), $k_{5,\ solution}$ (l = 4), $k_{5,\ solution}$ (l ≥ 5). All other rate constants are length independent. With the adjustments in our kinetic description, we describe the concentration profiles of all library members well (Fig. 3b-d, Fig. S1a-d, 2, 3 Table S5). Note that we included a penalty that suppresses strong length dependence to avoid unrealistically strong length dependence and overfitting the data.

The EDC decay fixes the reaction rate constant for the activation $k_{1,\ solution}$ uniquely (Fig. S1k). The decay profile fixes the reaction rate constant for the hydrolysis $k_{5,\ solution}$ uniquely (Fig. S1l). The reaction rates of hydrolysis of O-acylisourea $k_{3,\ solution,}$ and rearrangement of O-acylisourea to N-acylisourea $k_{4,\ solution}$ depend linearly on the reaction rate of ligation $k_{2,\ solution}$. Here, the experimental data fixes the ratio of the reaction rate constants $k_{2,\ solution}$, $k_{3,\ solution}$, and $k_{4,\ solution}$, but not their absolute values. (Fig. S1m, n). Thus, we are free to choose the absolute values of those reaction rate constants as small as possible to minimize computational cost as long as we keep the correct ratio (Fig. S1k-o). Consequently, the absolute rate constants need to be treated with caution.

Kinetic model with template

We extended our kinetic model to include the role of templation. We used the same model to describe the rate of all the reactions occurring in the solution. However, we added reactions that could occur on the template by defining oligomer-template complexes. The oligomer length and position on the template uniquely characterize each oligomer-template complex. We use the short-hand notation $\mathrm{O}^d\!:\mathrm{T}(\boldsymbol{l},\boldsymbol{i},\boldsymbol{g})$ for a complex that contains d oligomers. $\boldsymbol{l}$ and $\boldsymbol{i}$

are d-dimensional vectors describing the length and starting position of each oligomer in the complex; $\boldsymbol{g}$ is a d-dimensional vector denoting each oligomer's terminal group in the complex. Noteworthy, we do not allow oligomers to overlap on the template, but allow overhangs beyond the template length. We expect unactivated, activated oligomers and oligomers with N-acylisourea-group can form complexes with the template. We considered a maximum oligomer length of $l_{\text{max}} = 7$. In this case, N ≈ 1.6×10^8 complexes need to be considered. We reduced the number of relevant complexes to N ≈ 3.0×10^5 by neglecting complexes of activated oligomers on the template and complexes containing more than four oligomers. Complexes with more than four oligomers are insignificant since the template size limits the number of hybridized oligomers.

We ignored the O-acylisourea formation on the template due to the computational complexity. Instead, the model assumes that after reacting with fuel, the activated species immediately reacts with a neighboring carboxylate ($k_{2,\ template}$) or rearranges to the side product N-acylisourea ($k_{4,\ template}$). Further, we describe the spontaneous hydrolysis of oligomers on the template as a pseudo-first-order reaction ($k_{5,\ template}$).

To calculate the reaction rates, the model predicted the concentration of each library member in the solution and on the template. The concentrations on the template and in the solution are calculated (*vide infra*) using the dissociation constants between the library members and the template. For the monomer, we used the experimentally determined value of the dissociation constant. For the oligomers, we assumed they are length dependent, in line with the literature (Table S8).[10,11] Noteworthy, we compute the dissociation constants of template-oligomer complexes containing multiple oligomers as the product of the respective oligomer dissociation constants (equation 2).

$$K_d(\mathrm{O}^d\colon\mathrm{T})(\boldsymbol{l},\boldsymbol{i},\boldsymbol{g}) = \prod_{j=1}^{d} K_d(l_j) \qquad \textit{Equation 2}$$

Here, d is the total number of oligomers contained in the complex and $K_d(l_j)$ is the dissociation constant of a single oligomer of length l_j. In this parametrization, the affinity of the complex for an oligomer is independent of the oligomers already hybridized to the template.

The model integrates the chemical rate equations numerically using the Runge-Kutta-Fehlberg method (RK45) to calculate the concentrations in solution and on the template. For each time step, we have two steps. First, the model integrates slow chemical reactions, subsequently, the model determines the fast hybridization/dehybridization equilibrium concentrations by solving equations 3-5. Finally, the new set of concentrations is the input for the next time step.

We split the integration since timescales of hybridization/dehybridization are up to 6 orders of magnitude higher than chemical reactions, *i.e.*, $\tau_{hybridization} \lesssim \tau_{polymerization}$.[12-14]

The hybridization/dehybridization equilibrium is computed by its chemical constraints as follows.

Due to the *law of mass action*, the equilibrium concentration of any complex is

$$[\mathrm{O}^d\!:\mathrm{T}(\boldsymbol{l},\boldsymbol{i},\boldsymbol{g})] = \frac{[\mathrm{T}]\prod_{j=1}^{d}[\mathrm{O}(l_j,g_j)]}{K_d\big(\mathrm{O}^d\!:\mathrm{T}(\boldsymbol{l},\boldsymbol{i},\boldsymbol{g})\big)} \qquad \textit{Equation 3}$$

According to *template mass conservation*, the total concentration of the template stays constant at any instance in time as DNA hydrolysis is slow compared to all experimental timescales.

$$[\mathrm{T}] + \sum_{\{d,\boldsymbol{l},\boldsymbol{i},\boldsymbol{c}\}} [\mathrm{O}^d\!:\mathrm{T}(\boldsymbol{l},\boldsymbol{i},\boldsymbol{c})] = [\mathrm{T}]_{total} \qquad \textit{Equation 4}$$

Here, $\{d,\boldsymbol{l},\boldsymbol{i},\boldsymbol{c}\}$ is the sum over all possible complexes.

The *oligomer mass conservation* states that the total concentration of oligomers of any given type at a given instance in time needs to be conserved,

$$[\mathrm{O}(l,g)] + \sum_{\{d,\boldsymbol{l},\boldsymbol{i},\boldsymbol{g} \mid \exists j,\, s.t.\ l_j=l \wedge g_j=g\}} w(d,\boldsymbol{l},\boldsymbol{i},\boldsymbol{g})[\mathrm{O}^d\!:\mathrm{T}(\boldsymbol{l},\boldsymbol{i},\boldsymbol{g})] = [\mathrm{O}(l,g)]_{total} \qquad \textit{Equation 5}$$

Here, $\{d,\boldsymbol{l},\boldsymbol{i},\boldsymbol{g} \mid \exists j,\ s.t.\ l_j = l \wedge g_j = g\}$ is the set of complexes that contain at least one oligomer of length l and terminal group g. In general, a complex can have more than one oligomer of this type. Thus, we multiplied the complex concentration by a corresponding integer weight $w(d,\boldsymbol{l},\boldsymbol{i},\boldsymbol{g})$.

We combined these equations into a set of 3 l_{max} + 1 coupled polynomial equations of power *d*, which we solved numerically to obtain the concentrations of oligomer and complex at equilibrium.

The kinetic model with the template is parametrized as follows: The reaction rate constants for reactions in solution are the same as in the system without template. For the ligation reaction $k_{2,\, template}$ and the side product formation $k_{4,\, template}$, we assumed that the rate constants are in a similar order of magnitude as the activation rate in the solution $k_{1,\, solution}$ because the activation rate constant is rate limiting. However, $k_{2,\, template}$, and $k_{4,\, template}$ cannot be identical as they must reproduce the experimentally measured branching ratio between ligation and side-product

formation (Table S5). The parameter set does not reflect the full chemistry of templated ligation. Still, it provides a coarse-grained description that enables us to check if the hypothesized features of template-directed oligomerizations agree with our experimental observations.

Supplementary Tables

Table S1. *Characterization of backbone length per repetition unit.*

Bond type		Bond length
**C1 – C2**	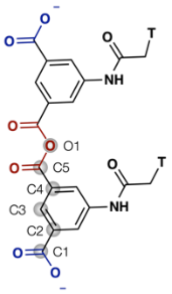	1.48524
**C2 – C3**		1.40119
**C3 – C4**		1.40389
**C4 – C5**		1.48722
**C5 – O1**		1.36201
**Sum**		7.13958
**P1 – O1**	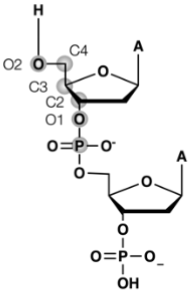	1.745558
**O1 – C1**		1.40362
**C1 – C2**		1.54298
**C2 – C3**		1.54248
**C3 – O2**		1.41664
**Sum**		7.65127

Table S2. *Characterization of precursors.*

Name	Structure	Exact mass ($gmol^{-1}$)	Mass found ($gmol^{-1}$)	Retention time (min)	Calibration value ($m.a.u.mM^{-1}$)
**EDC**	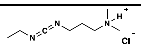	191.12<br>$C_8H_{17}N_3$	N/a	1.7	5.76 (220 nm)<br>0.0075<br>(260 nm)
**EDU**	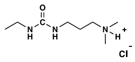	209.13<br>$C_8H_{19}N_3O$	N/a	N/a	N/a
**Pyridine**	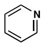	79.04<br>C_5H_5N	N/a	1.5	3.05 (260 nm)
**T**	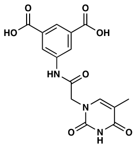	347.08<br>$C_{15}H_{13}N_3O_7$	$[M\text{-}H]^-$ = 346.21<br>Error = 404.54<br>$[2\times M\text{-}H]^-$ = 692.72	8.8	13.4 (260 nm)<br>3.2 (290 nm)
**Me**	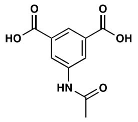	223.05<br>$C_{10}H_9NO_5$	$[M\text{-}H]^-$ = 222.04<br>Error = 0	8.5	6.1 (260 nm)<br>0.99 (290 nm)
**C**	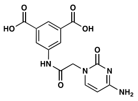	332.08<br>$C_{14}H_{12}N_4O_6$	$[M\text{-}H]^-$ = 331.13<br>Error = 181.23	5.9	9.65 (260 nm)<br>8.69 (290 nm)
**U**	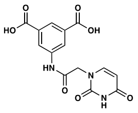	333.06<br>$C_{14}H_{11}N_3O_7$	$[M\text{-}H]^-$ = 332.05<br>Error = 1204.63	7.5	17.26<br>(260 nm)<br>1.55 (290 nm)
**Me-T**	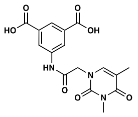	361.09<br>$C_{16}H_{15}N_3O_7$	$[M\text{-}H]^-$ = 360.48<br>Error = 1110.86	10.5	14.70<br>(260 nm)<br>2.49 (290 nm)
**Me-C**	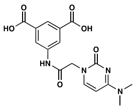	360.11<br>$C_{16}H_{16}N_4O_6$	$[M\text{-}H]^-$ = 361.26<br>Error = 415.38	6.2	8.09 (260 nm)<br>10.2 (290 nm)
**3-pyridyl IPA**	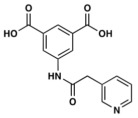	300.07<br>$C_{15}H_{12}N_2O_5$	$[M\text{-}H]^-$ = 299.76<br>Error = 2273.71	6.3	11.6 (260 nm)<br>0.73 (290 nm)
**Benzylamine**	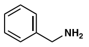	107.07<br>C_7H_9N	N/a	3.3	N/a

Name	Structure	Exact mass (gmol^{-1})	Mass found (gmol^{-1})	Retention time (min)	Calibration value (m.a.u.mM^{-1})
**Benzylamine**	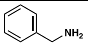	107.07<br>C_7H_9N	N/a	3.3	N/a
**BA-T**	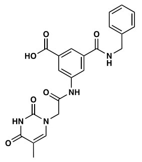	436.14<br>$C_{22}H_{20}N_4O_6$	$[M+H]^+$ = 436.93<br>Error = -503.25<br>$[2\times M+H]^+$ = 872.45	15.9	3.2 (290 nm)
**BA-C**	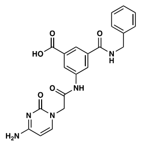	421.14<br>$C_{21}H_{19}N_5O_5$	$[M-H]^+$ = 422.12<br>Error = -71.06	14.5	8.69 (290 nm)
**BA-2C**	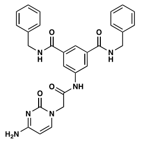	510.20<br>$C_{28}H_{26}N_6O_4$	$[M-H]^+$ = 511.09<br>Error = -234.73	16.7	8.69 (290 nm)
**BA-U**	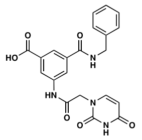	422.19<br>$C_{21}H_{18}N_4O_6$	$[M-H]^+$ = 423.05<br>Error = -189.06	15.4	1.55 (290 nm)
**BA-2U**	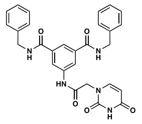	511.19<br>$C_{28}H_{25}N_5O_6$	$[M-H]^+$ = 512.15<br>Error = -78.09	16.9	1.55 (290 nm)
**$(dA)_{10}$**	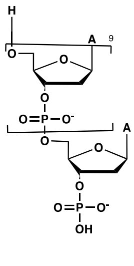	3068.62<br>$C_{100}H_{121}N_{50}O_{48}P_9$	$[M- 2\times H]^{2-}$ = 1533.42, 1533.91, 1534.42, 1535.42<br>$[M- 3\times H]^{3-}$ = 1021.91, 1022.25, 1022.59<br>$[M- 6\times H + Na^+]^{6-}$ = 515.21, 516.21, 517.28	n.a.	n.a.

Table S3. *Characterization of library members made with* ***T****. * in* ***C/T*** *mixtures.*

**Name**	**Structure**	**Exact mass ($gmol^{-1}$)**	**Mass found ($gmol^{-1}$)**<br>**Error (ppm)**	**Retention time (min)**	**Calibration value ($m.a.u.mM^{-1}$)**
**DynT$_2$**	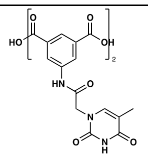	676.14<br>$C_{30}H_{24}N_6O_{13}$	$[M-H]^-$ = 675.15<br>Error = 29.62	14.5	18.73<br>(260 nm)
**DynT$_3$**	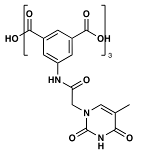	1005.20<br>$C_{45}H_{39}N_9O_{19}$	$[M-H]^-$ =<br>1003.83<br>Error = -368.45	16.7/17*	22.29<br>(260 nm)
**DynT$_4$**	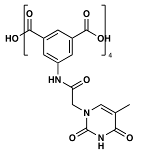	1335.09<br>$C_{60}H_{46}N_{12}O_{25}$	$[M-H]^-$ =<br>1333.92<br>Error = 495.02<br>$[M- 2\times H]^{2-}$ =<br>666.07<br>Error = -90.072	17.3	29.48<br>(260 nm)
**DynT$_5$**	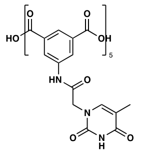	1664.36<br>$C_{75}H_{57}N_{15}O_{31}$	$[M-H]^-$ =<br>1662.32,<br>Error = -6.01<br>$[M- 2\times H]^{2-}$ =<br>830.46<br>Error = -240.77	17.5	40.16<br>(260 nm)
**cDynT$_3$**	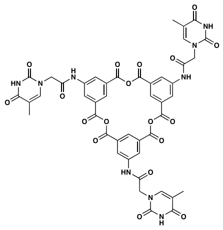	987.80<br>$C_{45}H_{33}N_9O_{18}$	$[M-H]^-$ = 986.01<br>Error = -182.52	17.8	22.29<br>(260 nm)

Table S4. *Characterization of side products made with* ***T****.*

Name	Structure	Exact mass (gmol^{-1})	Mass found (gmol^{-1}) Error (ppm)	Retention time (min)	Calibration value (m.a.u.mM^{-1})
**T***	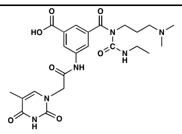	502.53<br>$C_{23}H_{30}N_6O_7$	$[M-H]^-$ = 501.50<br>Error = 578.59<br>$[2\times M-H]^-$ = 1002.59<br>Error = -827.17	7.9	3.2 (290 nm)
T	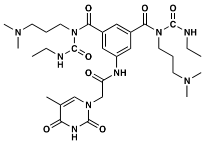	657.36<br>$C_{31}H_{47}N_9O_7$	$[M-H]^-$ = 656.51<br>Error = 289.49	7.3	3.2 (290 nm)
**DynT$_2$***	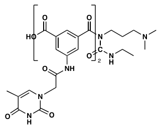	831.80<br>$C_{38}H_{41}N_9O_{13}$	$[M-H]^-$ = 831.03<br>Error = 915.36	13.8	5.89<br>(290 nm)
**DynT$_3$***	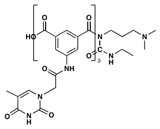	1161.06<br>$C_{53}H_{52}N_{12}O_{19}$	$[M-H]^-$ = 1158.78<br>Error = -483.03	15.9	5.57<br>(290 nm)

Table S5. *Rate constants.*

Rate constants	Wo oligomer length dependence	W oligomer length dependence	On template
**Activation**			
**k_1_T $(mMh)^{-1}$**	0.2	0.19	
**k_1_T_2 $(mMh)^{-1}$**	0.2	0.33	
**k_1_T_3 $(mMh)^{-1}$**	0.2	0.84	
**k_1_T_4 $(mMh)^{-1}$**	0.2	2.37	
**k_1_$T_{n>4}$ $(mMh)^{-1}$**	0.2	2.06	
**Ligation**			
**$k_{2,\ solution}$ $(mMh)^{-1}$**	5557	77.96	0.6
**Hydrolysis O-acylisourea**			
**k_3 $(h)^{-1}$**	2.5	514.82	0
**Formation of N-acylisourea**			
**k_4 $(h)^{-1}$**	6957	179.91	0.2
**Hydrolysis**			
**k_5_T_2 $(h)^{-1}$**	29.1	26.9	0
**k_5_T_3 $(h)^{-1}$**	29.1	17.81	0
**k_5_T_4 $(h)^{-1}$**	29.1	6.21	0
**k_5_$T_{n>4}$ $(h)^{-1}$**	29.1	3.06	0

Table S6. *Evidence of hybridization for hybridizing **T** library with (dA)$_{10}$ by mass spectrometry.*

Name	Exact mass (gmol^{-1})	Mass expected (gmol^{-1})	Mass found (gmol^{-1})
[T_1 + (dA)$_{10}$]	3415.9	[T_1 + (dA)$_{10}$ - 2H]$^{2-}$ = 1137.63	[T_1 + (dA)$_{10}$ - 2H]$^{2-}$ = 1137.01
[T_2 + (dA)$_{10}$]	3745.18	[T_2 + (dA)$_{10}$ - 5H + 2Na$^+$]$^{5-}$ = 757.2	[T_2 + (dA)$_{10}$ - 5H + 2Na$^+$]$^{5-}$ = 757.62
		[T_2 + (dA)$_{10}$ - 8H]$^{8-}$ = 467.14	[T_2 + (dA)$_{10}$ - 8H]$^{8-}$= 467.98
		[T_2 + (dA)$_{10}$ - 9H]$^{9-}$ = 415.13	[T_2 + (dA)$_{10}$ - 9H]$^{9-}$ = 415.15
[T_3 + (dA)$_{10}$]	4074.46	[T_3 + (dA)$_{10}$ - 3H]$^{3-}$ = 1357.15	[T_3 + (dA)$_{10}$ - 3H]$^{3-}$ = 1357.18
		[T_3 + (dA)$_{10}$ - 4H]$^{4-}$ = 1017.61	[T_3 + (dA)$_{10}$ - 4H]$^{4-}$ = 1017.81
		[T_3 + (dA)$_{10}$ - 7H]$^{7-}$ = 581.06	[T_3 + (dA)$_{10}$ - 7H]$^{7-}$ = 581.1197, 582.12
		[T_3 + (dA)$_{10}$ - 8H]$^{8-}$ = 508.3	[T_3 + (dA)$_{10}$ - 8H]$^{8-}$ = 508.04
		[T_3 + (dA)$_{10}$ - 9H + Na$^+$]$^{9-}$ = 454.27	[T_3 + (dA)$_{10}$ - 9H + Na$^+$]$^{9-}$ = 454.13
[T_4 + (dA)$_{10}$]	4403.71	[T_4 + (dA)$_{10}$ - 5H]$^{5-}$ = 1099.92	T_4 + (dA)$_{10}$ - 5H]$^{5-}$ = 1099.16
		[T_4 + (dA)$_{10}$ - 5H]$^{5-}$ = 879.74	[T_4 + (dA)$_{10}$ - 5H]$^{5-}$ = 879.732
		[T_4 + (dA)$_{10}$ - 5H + Na$^+$]$^{5-}$ = 884.47	[T_4 + (dA)$_{10}$ - 5H + Na$^+$]$^{5-}$ = 884.87
		[T_4 + (dA)$_{10}$ - 5H + 2Na$^+$]$^{5-}$ = 888.94	[T_4 + (dA)$_{10}$ - 5H + 2Na$^+$]$^{5-}$ = 888.10
[T_5 + (dA)$_{10}$]	4732.98	[T_5 + (dA)$_{10}$ - 8H]$^{8-}$ = 590.63	[T_5 + (dA)$_{10}$ - 8H]$^{8-}$ = 590.03
		[T_5 + (dA)$_{10}$ - 8H + Na$^+$]$^{8-}$ = 593.49	[T_5 + (dA)$_{10}$ - 8H + Na$^+$]$^{8-}$ = 593.03

Table S7. *Experimental conditions of gel electrophoresis.*

Nr.	Conditions
**1**	7 mM $(dA)_{35}$ – Cy5 + 2 mM $(dT)_{10}$ (expressed in monomer concentration) + 100 mM NaCl +12.5 mM $MgCl_2$ in buffer MES 200 mM, pH6
**2**	7 mM $(dA)_{35}$ – Cy5 (expressed in monomer concentration) + 100 mM NaCl in buffer MES 200 mM, pH 6
**3**	7 mM $(dA)_{35}$ – Cy5 (expressed in monomer concentration) + 25 mM EDC + 100 mM NaCl in buffer MES 200 mM, pH6
**4**	7 mM $(dA)_{35}$ – Cy5 (expressed in monomer concentration) + 25 mM EDC + 10 mM pyridine + 100 mM NaCl in buffer MES 200 mM, pH 6
**5**	7 mM $(dA)_{35}$ – Cy5 (expressed in monomer concentration) + 25 mM T + 10 mM pyridine + 100 mM NaCl in buffer MES 200 mM, pH6
**6**	7 mM $(dA)_{35}$ – Cy5 (expressed in monomer concentration) + 25 mM EDC + 10 mM pyridine + 25 mM T + 100 mM NaCl in buffer MES 200 mM, pH 6

Table S8. *Dissociation constants of monomers and oligomers.*

Library members	Dissociation constant K_D (mM)
**T**	$K_D(1)$ = 90 mM
**DynT$_2$**	$K_D(2)$ = 1.3 mM
**DynT$_3$**	$K_D(3)$ = 0.1 mM
**DynT$_4$**	$K_D(4)$ = 0.51 mM
**DynT$_5$**	$K_D(5)$ = 0.92 mM

__Table S9.__ Characterization of library members made with __U__.

Name	Structure	Exact mass (gmol^{-1})	Mass found (gmol^{-1}) Error (ppm)	Retention time (min)	Calibration value (m.a.u.mM^{-1})
**DynU$_2$**	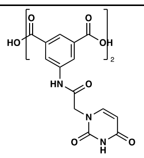	648.11<br>$C_{28}H_{20}N_6O_{13}$	$[M-H]^-$ = 647.42<br>Error = 494.15	12.8	27.01<br>(260 nm)
**DynU$_3$**	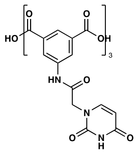	963.16<br>$C_{42}H_{29}N_9O_{19}$	$[M-H]^-$ = 962.31<br>Error = 166.29	14.3	34.52<br>(260 nm)
**DynU$_4$**	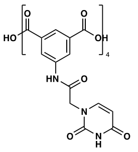	1278.21<br>$C_{56}H_{38}N_{12}O_{25}$	$[M-H]^-$ 1276.83<br>Error = -269.89	14.8	37.81<br>(260 nm)
**DynU$_5$**	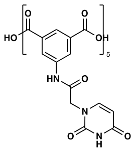	1593.26<br>$C_{70}H_{47}N_{15}O_{31}$	$[M-H]^-$ =<br>1591.89<br>Error = -226.09	15.2	40.41<br>(260 nm)
**cDynU$_3$**	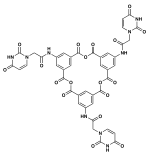	945.15<br>$C_{42}H_{27}N_9O_{18}$	$[M-H]^-$ = 944.09<br>Error = -52.95	N/a	34.52<br>(260 nm)

Table S10. *Characterization of side products made with* ***U***.

**Name**	**Structure**	**Exact mass ($gmol^{-1}$)**	**Mass found ($gmol^{-1}$) Error (ppm)**	**Retention time (min)**	**Calibration value ($m.a.u.mM^{-1}$)**
**U***	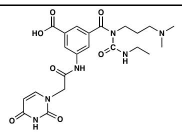	488.19<br>$C_{21}H_{26}N_6O_7$	$[M\text{-}H]^-$ = 487.69<br>Error = 1064.84	8.3	17.26<br>(260 nm)<br>1.55<br>(290 nm)
U	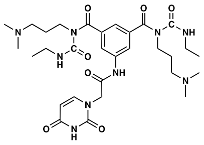	643.33<br>$C_{49}H_{43}N_9O_7$	$[M\text{-}H]^-$ = 642.14<br>Error = -1849.75	8.7	17.26<br>(260 nm)<br>1.55<br>(290 nm)

Table S11. _Characterization of library members made with **C**._

Name	Structure	Exact mass ($gmol^{-1}$)	Mass found ($gmol^{-1}$) Error (ppm)	Retention time (min)	Calibration value ($m.a.u.mM^{-1}$)
**DynC$_2$**	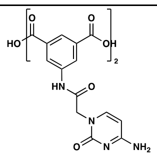	646.14<br>$C_{28}H_{22}N_8O_{11}$	$[M-H]^-$ = 645.68<br>Error = 852.54	12.5	23.28<br>(260 nm)
**DynC$_3$**	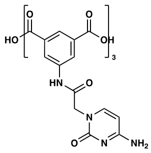	960.21<br>$C_{42}H_{32}N_{12}O_{16}$	$[M-H]^-$ = 959.12<br>Error = -83.40	13.8	25.98<br>(260 nm)
**DynC$_4$**	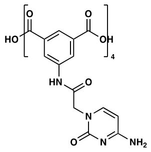	1274.27<br>$C_{56}H_{42}N_{16}O_{21}$	$[M-3H+3\times Na^+]^{3-}$ = 446.69<br>Error = 44.77	16.9	27.82<br>(260 nm)

Table S12. *Characterization of side products made with **C**.*

Name	Structure	Exact mass ($gmol^{-1}$)	Mass found ($gmol^{-1}$) Error (ppm)	Retention time (min)	Calibration value ($m.a.u.mM^{-1}$)
**C***	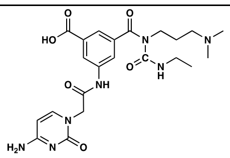	487.21 $C_{21}H_{27}N_{7}O_{6}$	$[M-H]^{-}$ = 486.10 Error = -226.23	6.8	8.69 (290 nm
C	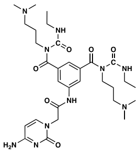	642.35 $C_{29}H_{44}N_{10}O_{6}$	$[M-H]^{-}$ = 642.98 Error = -226.23	7.2	8.69 (290 nm

__Table S13.__ Characterization of library members made with __C__ and __T__.

Name	Structure	Exact mass ($gmol^{-1}$)	Mass found ($gmol^{-1}$) Error (ppm)	Retention time (min)	Calibration value ($m.a.u.mM^{-1}$)
**Dyn(TC)**	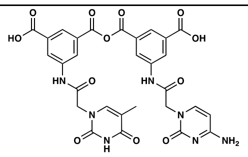	661.14<br>$C_{29}H_{23}N_7O_{12}$	$[M-H]^-$ =<br>660.46<br>Error = 499.90	13.3	21.00<br>(260 nm)
**Dyn(T_2C)**#	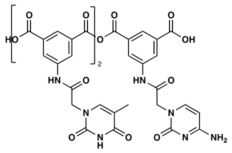	990.21<br>$C_{44}H_{34}N_{10}O_{18}$	$[M-H]^-$ =<br>989.18<br>Error = -20.21	15.69	23.552<br>(260 nm)

all isomers are possible.

Supplementary Figures

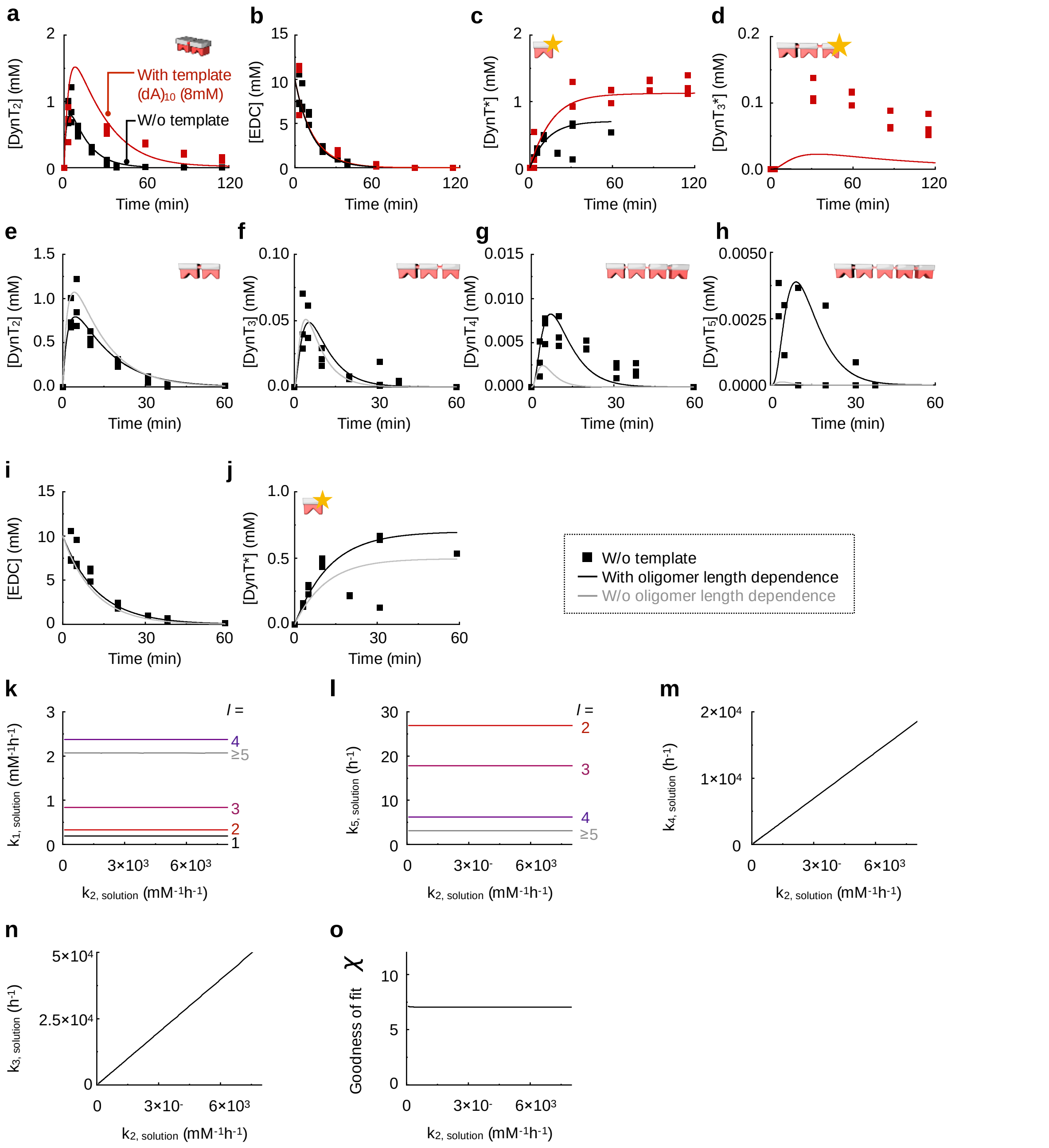

Figure S1. Kinetic characterization of the dynamic library with and without $(dA)_{10}$ by the kinetic model. (a-d) *Concentration as a function of time for* ***(a)*** ***$DynT_2$*** ***(b) EDC*** *and side products* ***(c) DynT**** *, and* ***(d)*** ***$DynT_3$**** *with and without 8 mM template $(dA)_{10}$ (expressed in monomer concentration).* ***(e-j)*** *Concentration as a function of time for* ***(e-h)*** *oligomers* ***$DynT_2$-$DynT_5$*** *,* ***(i) EDC*** *, and side product* ***(j) DynT**** *in solution. A kinetic model was used to fit the data, showing the kinetic model with (black) and without (grey) oligomer length-dependent activation and hydrolysis rate constants.* ***(k)*** *Activation rate $k_{1,\ solution}$ ($mM^{-1}h^{-1}$) and* ***(l)*** *anhydride hydrolysis rate $k_{5,\ solution}$ (h^{-1}) of different oligomers,* ***(m)*** *rearrangement rate $k_{4,\ solution}$ (h^{-1}) of O-acylisourea to N-acylisourea,* ***(n)*** *hydrolysis rate $k_{3,\ solution}$ (h^{-1}) of O-acylisourea and* ***(o)*** *the goodness of fit as a function $k_{2,\ solution}$ ($mM^{-1}h^{-1}$).*

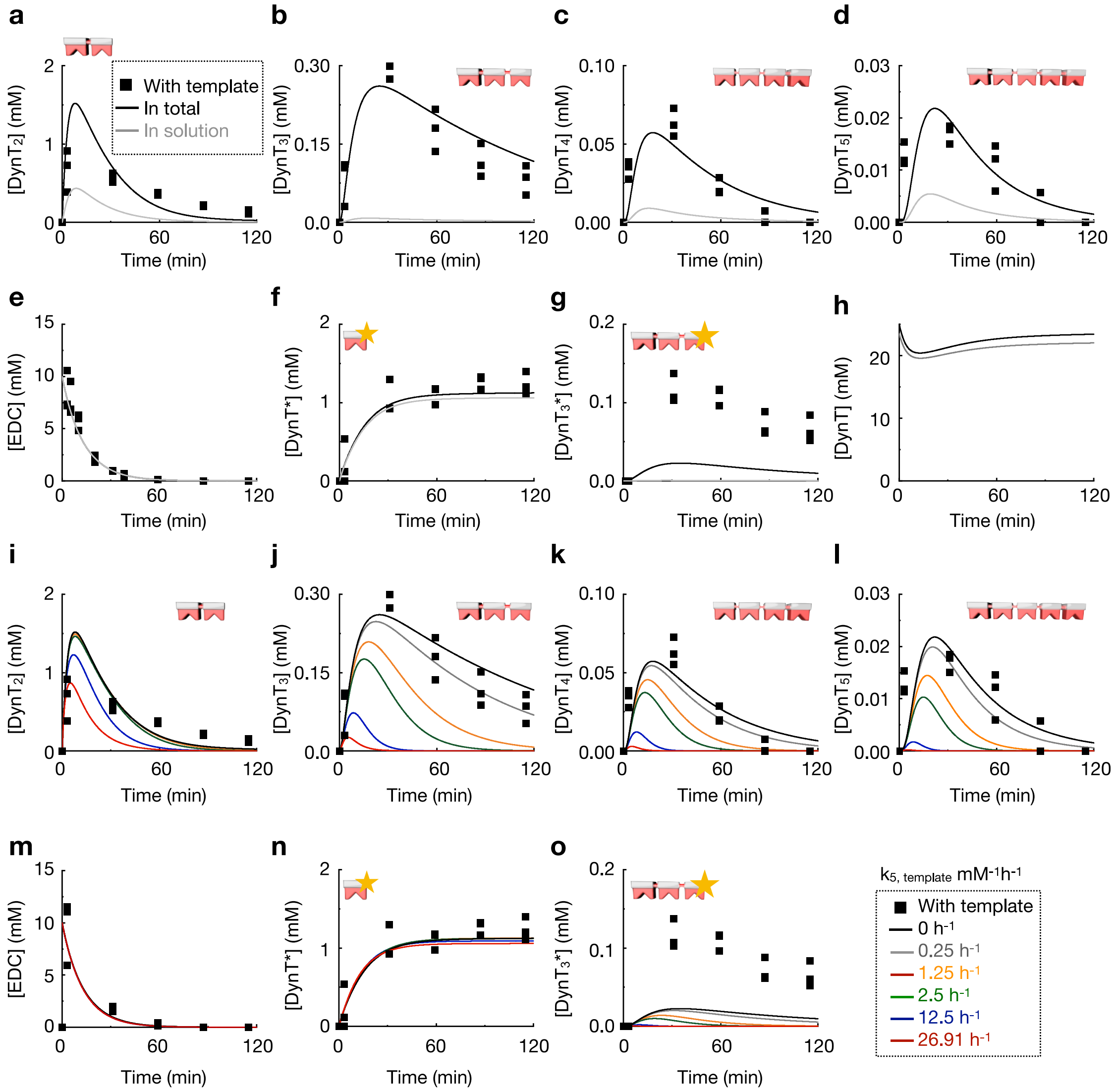

Figure S2. Kinetic characterization of the dynamic library with and without $(dA)_{10}$ by the kinetic model. (a-o) *Concentration as a function of time for* ***(a-d, i-l)*** *oligomers* ***$DynT_2$-$DynT_5$, (e, m) EDC****, side products* ***(f, n) DynT*****,* ***(g, o) $DynT_3$*****, and* ***(h) DynT*** *with 8 mM template $(dA)_{10}$ (expressed in monomer concentration). A kinetic model was used to fit the data, showing in* ***(a-h)*** *the fraction reacting in the solution (grey) and on the template (black). In* ***(i-o)*** *the kinetic model fits the data with increasing anhydride hydrolysis rates $k_{5,\ template}$ $mM^{-1}h^{-1}$.*

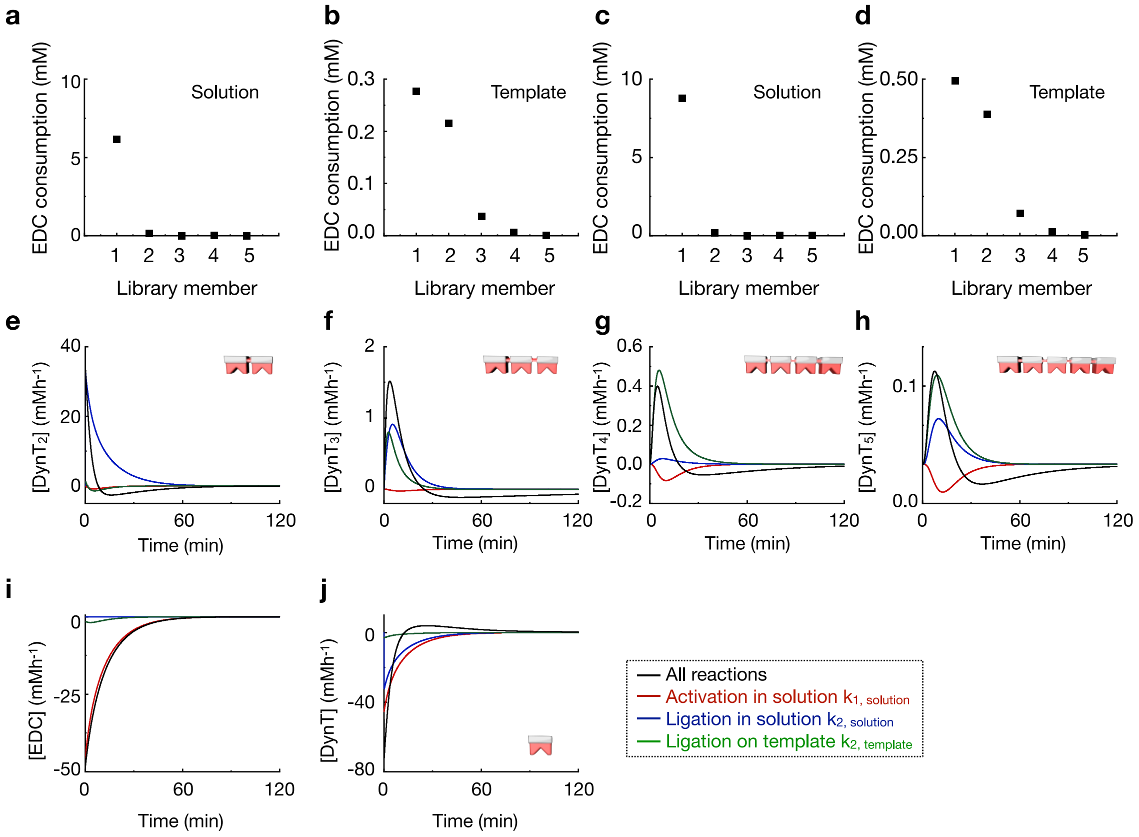

***Figure S3. Fuel consumption and dynamic library composition in solution and on the template.** **(a-d)** EDC consumption as a function for oligomers in the solution **(a, c)** and on the template **(b, d)** through ligation **(a, b)** or all reactions **(c, d)**. **(e-j)** Reaction rates for fuel and monomer consumption and the formation of oligomers **DynT$_2$-DynT$_5$** with 8 mM template (dA)$_{10}$ (expressed in monomer concentration) via activation in solution, ligation in solution, and ligation on the template.*

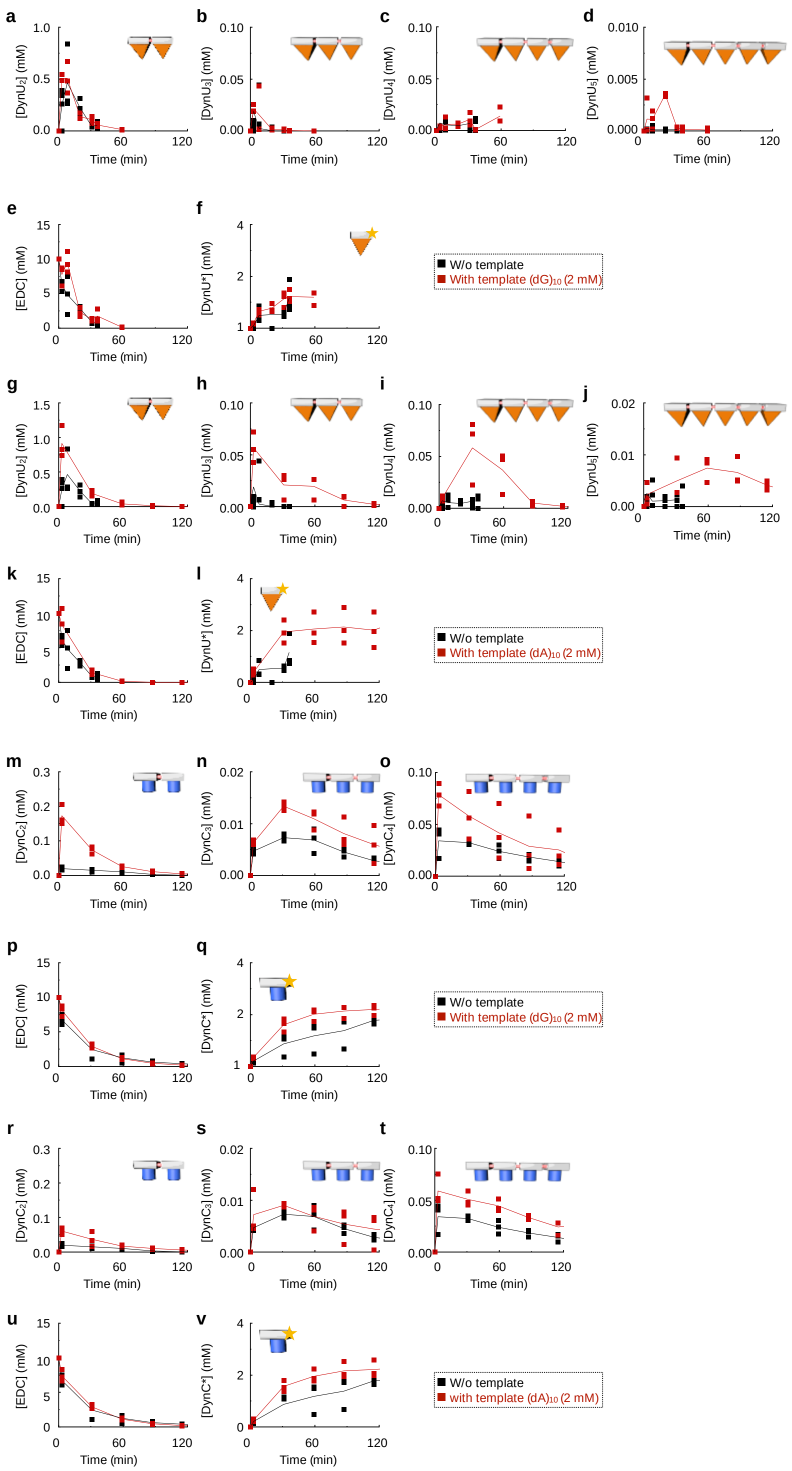

Figure S4. Kinetic characterization of the dynamic library as a function of template type. (a-j)** Concentration as a function of time for **(a-d, g-j)** oligomers **DynU$_2$-DynU$_5$**, **(e, k) EDC**, and side product **(f, l) DynU with 2 mM **(a-f)** template (dG)$_{10}$, or **(g-l)** template (dA)$_{10}$. **(m-v)** Concentration as a function of time for **(m-o, r-t)** oligomers **DynC$_2$-DynC$_4$**, **(p, u) EDC**, and side product **(q, v) DynC*** with 2 mM **(m-q)** template (dG)$_{10}$, or **(r-v)** template (dA)$_{10}$. Template concentrations are expressed in monomer concentration. Lines are guides for the eye, showing the evolution of the mean.*

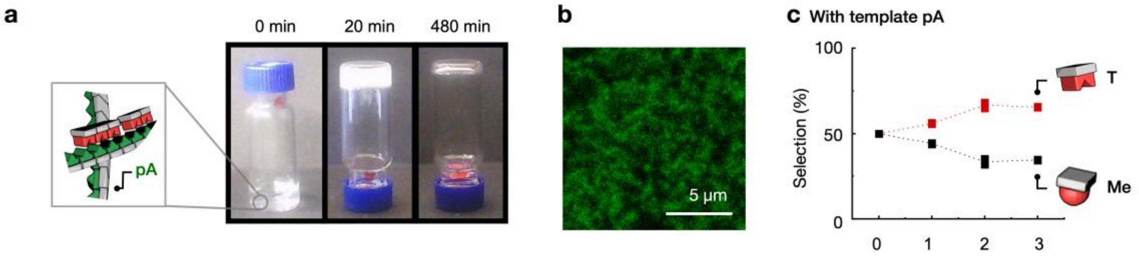

***Figure S5. Extracting T from mixed monomer pool. (a, b)** Photographs at different time points **(a)** and confocal micrograph at 30 minutes in the reaction cycle **(b)** when fueling **(a)** **T**/**Me**, in the presence of 4.31 mM pA (expressed in monomer concentration). **(c)** Composition of the hydrogel after each cycle for mixed **T**/**Me** pool.*

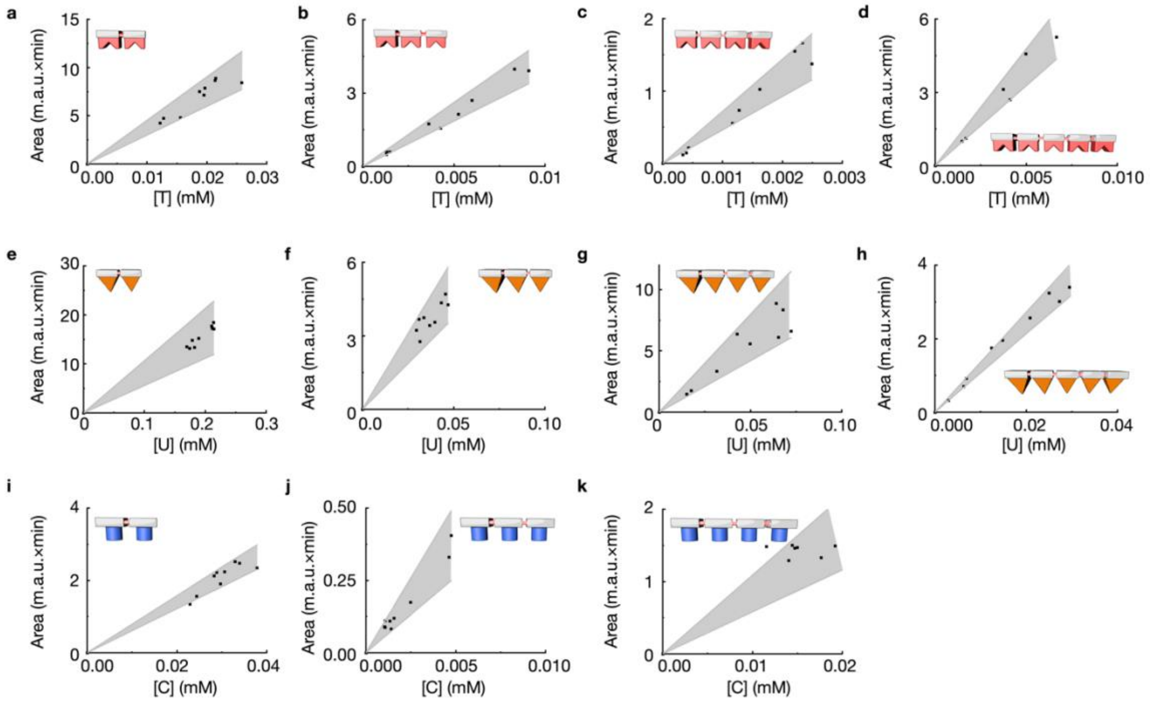

Figure S6. *Calibration curves of **DynT$_2$ (a)**, **DynT$_3$ (b)**, **DynT$_4$ (c)**, **DynT$_5$ (d)**, **DynU$_2$ (e)**, **DynU$_3$ (f)**, **DynU$_4$ (g)**, **DynU$_5$ (h)**, and **DynC$_2$ (i)**, **DynC$_3$ (j), DynC$_4$ (k)**. **(a-k)** Confidence intervals of 99% (grey) and standard error of the slope are shown.*

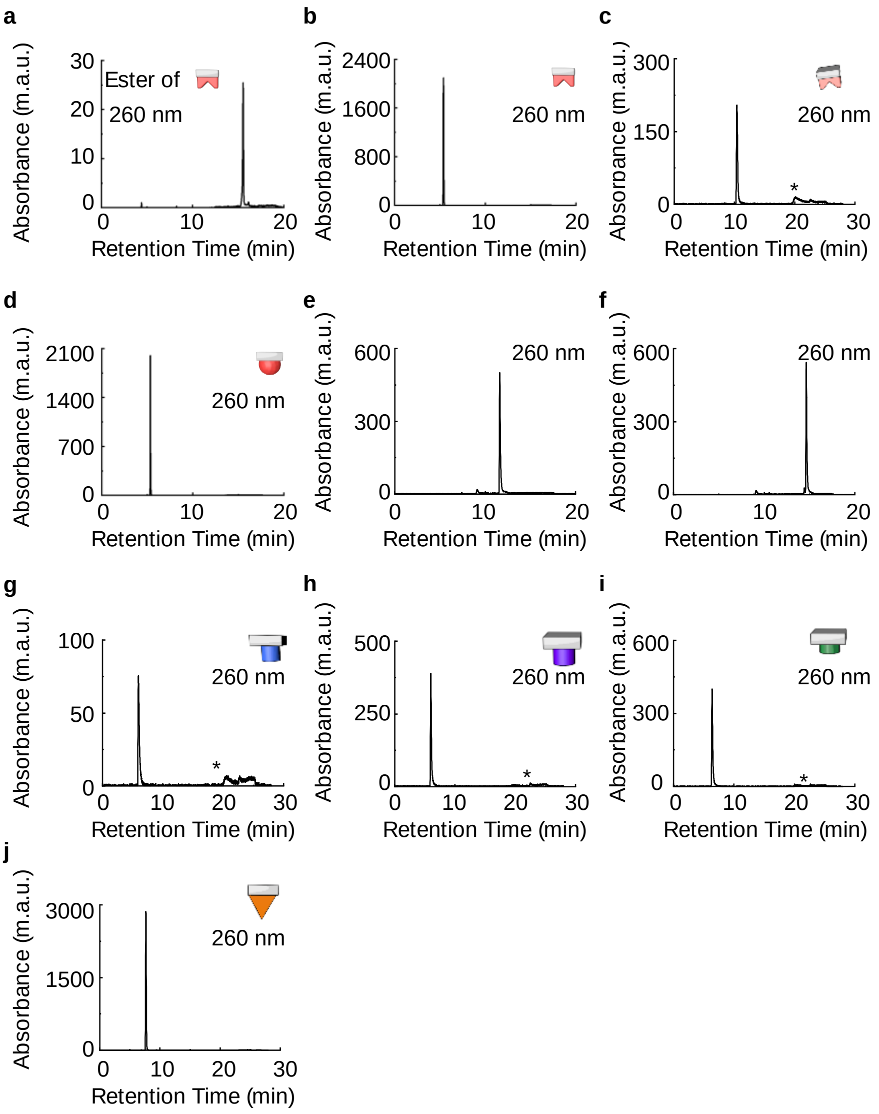

***Figure S7**. HPLC chromatogram of **(a) T**-ester, **(b) T**, **(c) Me-T**, **(d) Me**, **(e)** (Boc)$_2$-5-nitro-isophthalate, **(f)** (Boc)$_2$-5-amino-isophthalate, **(g) C**, **(h) Me-C**, **(i) 3-pyridyl IPA**, and **(j) U**.*
** impurities on the column.*

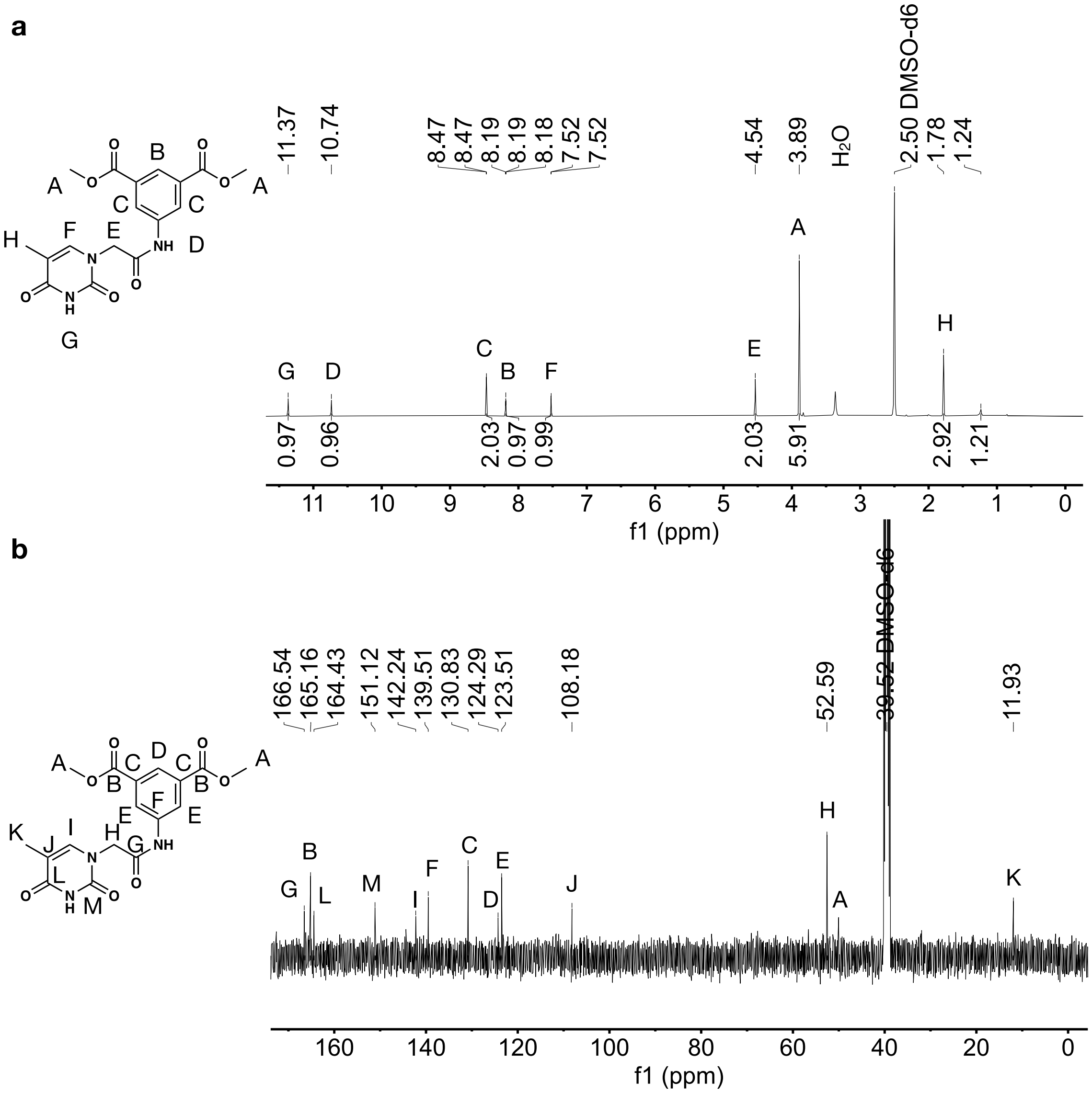

***Figure S8**. **(a)** ^{1}H-NMR and **(b)** ^{13}C-NMR spectra of **T**-ester.*

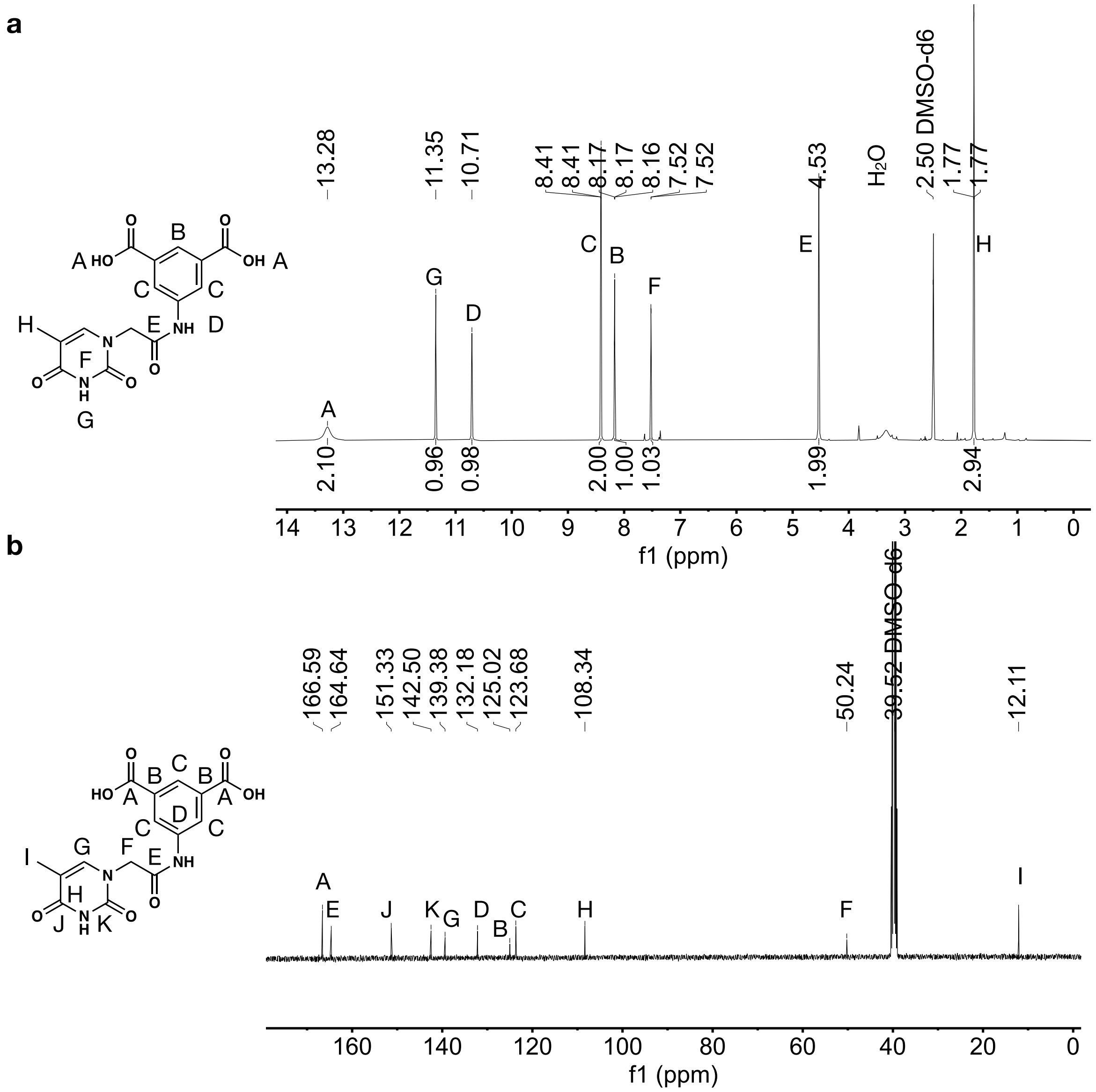

***Figure S9**. **(a)** ^{1}H-NMR and **(b)** ^{13}C-NMR spectra of **T**.*

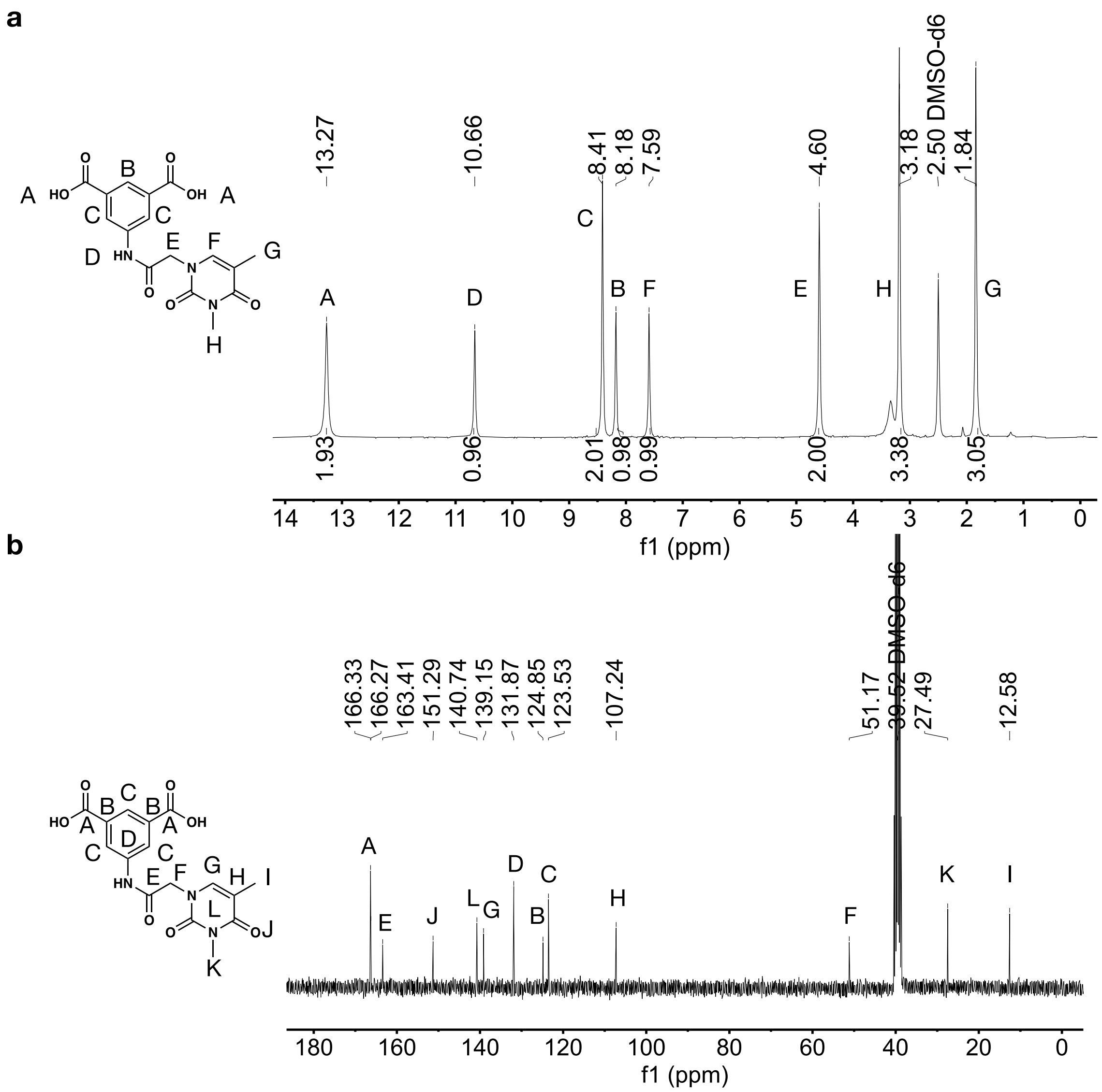

***Figure S10**. **(a)** ^{1}H-NMR and **(b)** ^{13}C-NMR spectra of **Me-T**.*

a

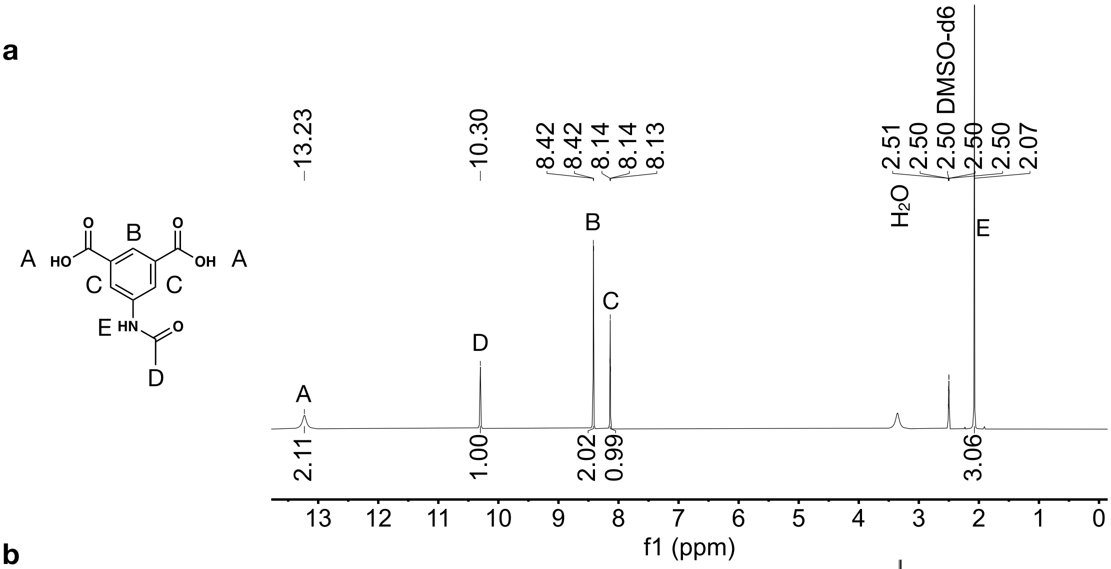

b

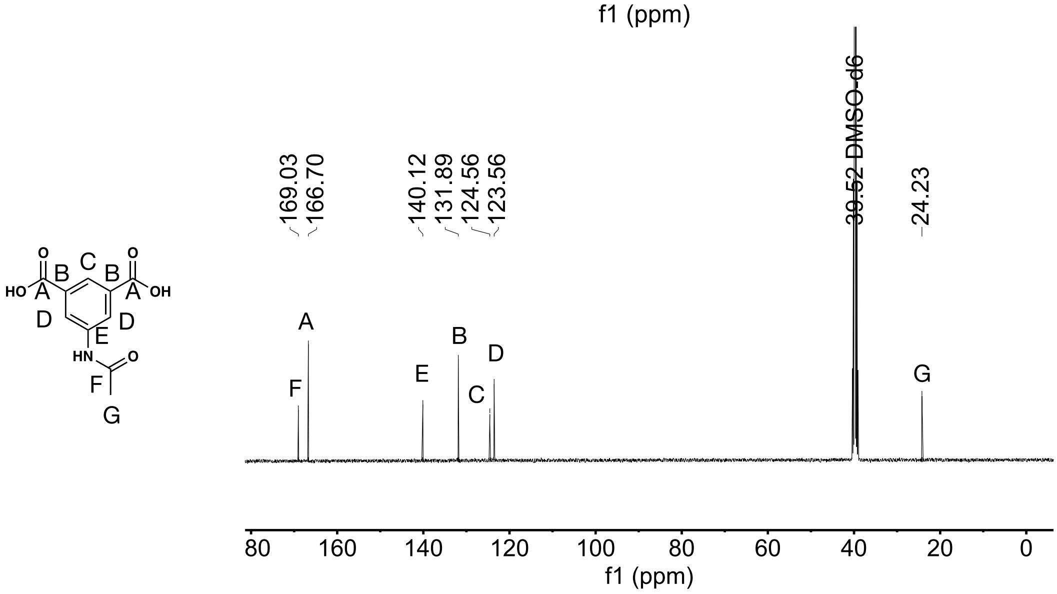

***Figure S11**. **(a)** ^{1}H-NMR and **(b)** ^{13}C-NMR spectra of **Me**.*

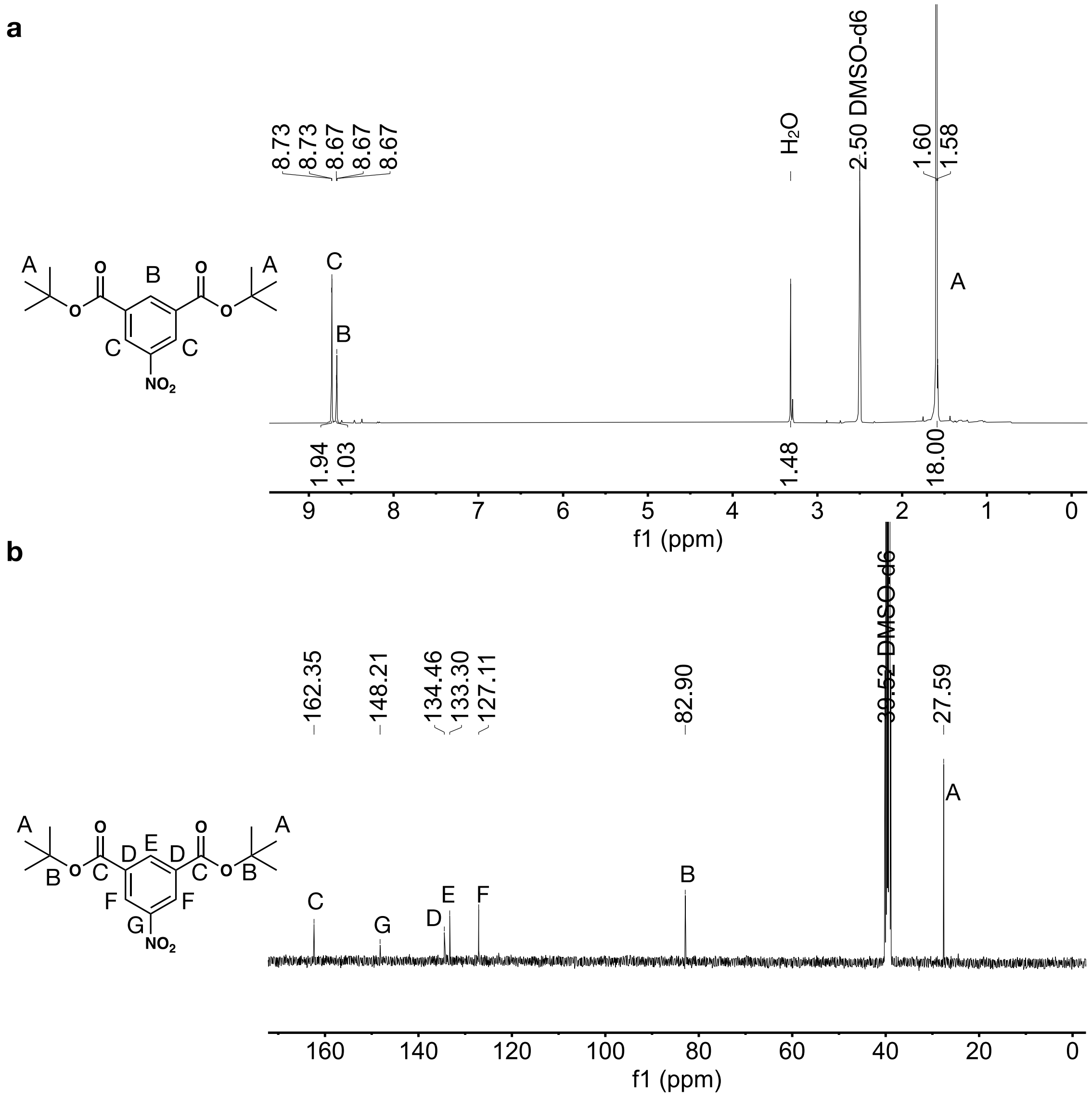

***Figure S12**. **(a)** ^{1}H-NMR and **(b)** ^{13}C-NMR spectra of (Boc)$_2$-5-nitroisophthalate.*

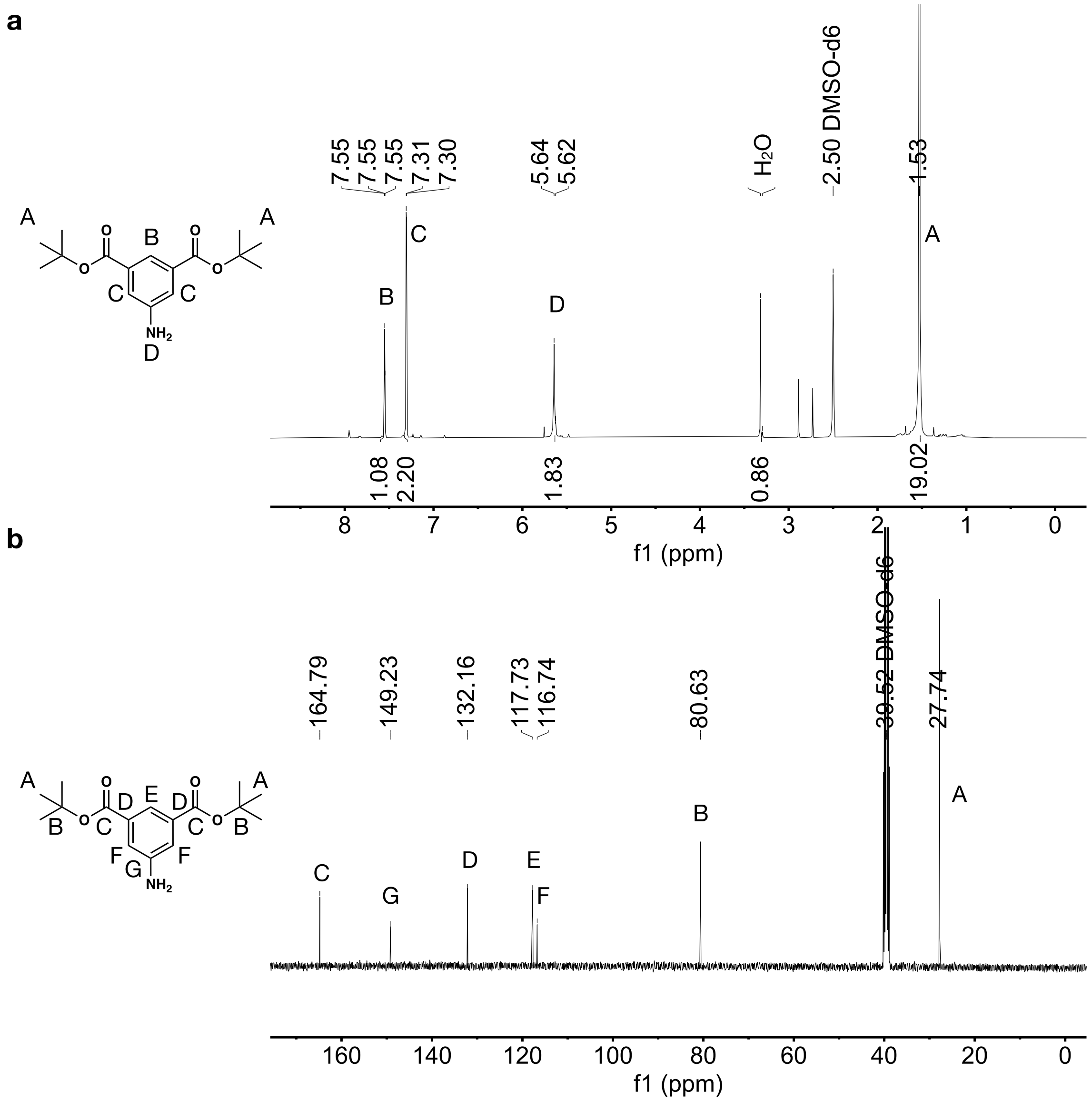

***Figure S13**. **(a)** ^{1}H-NMR and **(b)** ^{13}C-NMR spectra of Boc)$_2$-5-aminoisophthalate.*

a

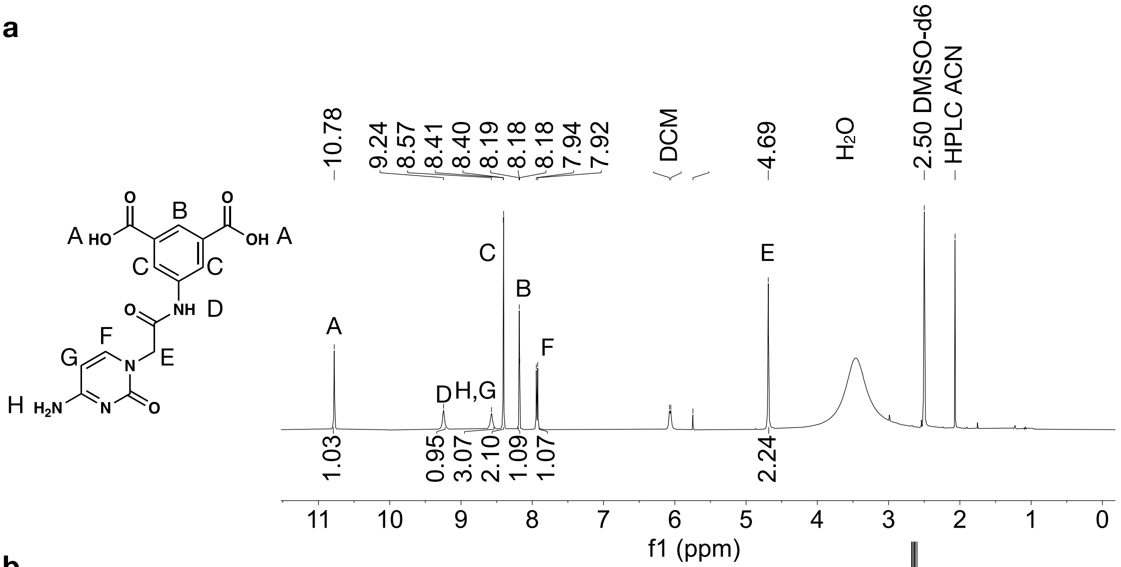

b

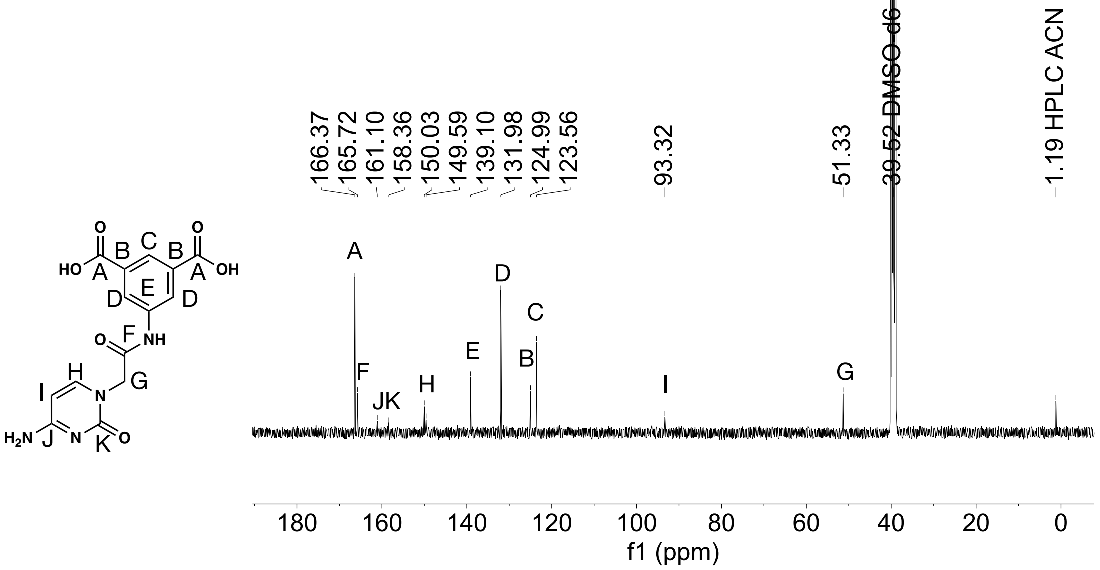

***Figure S14**. **(a)** ^{1}H-NMR and **(b)** ^{13}C-NMR spectra of **C**.*

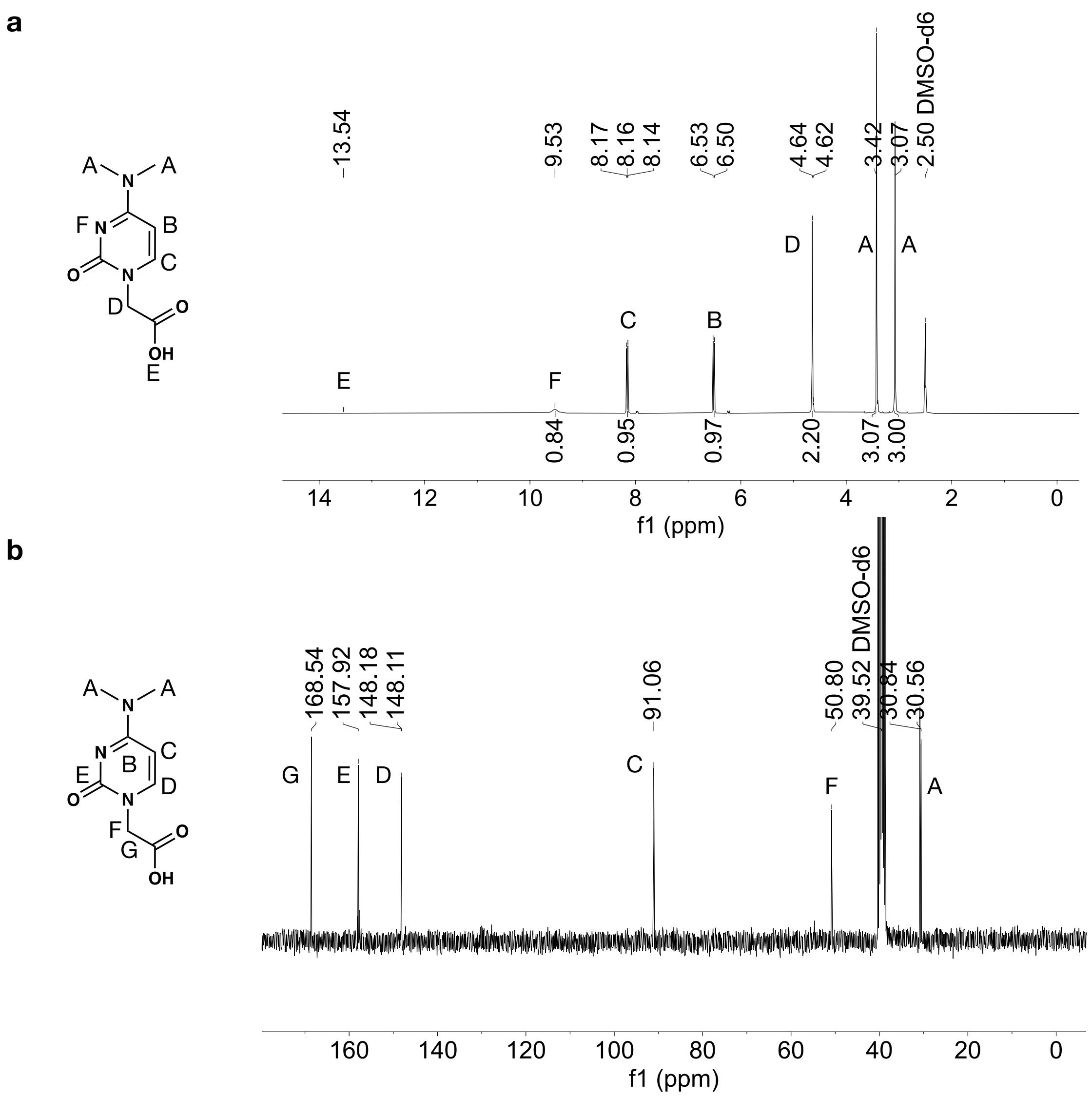

***Figure S15**. **(a)** ^{1}H-NMR and **(b)** ^{13}C-NMR spectra of N,N-dimethylcytosine-1-acetic acid ethyl acid.*

a

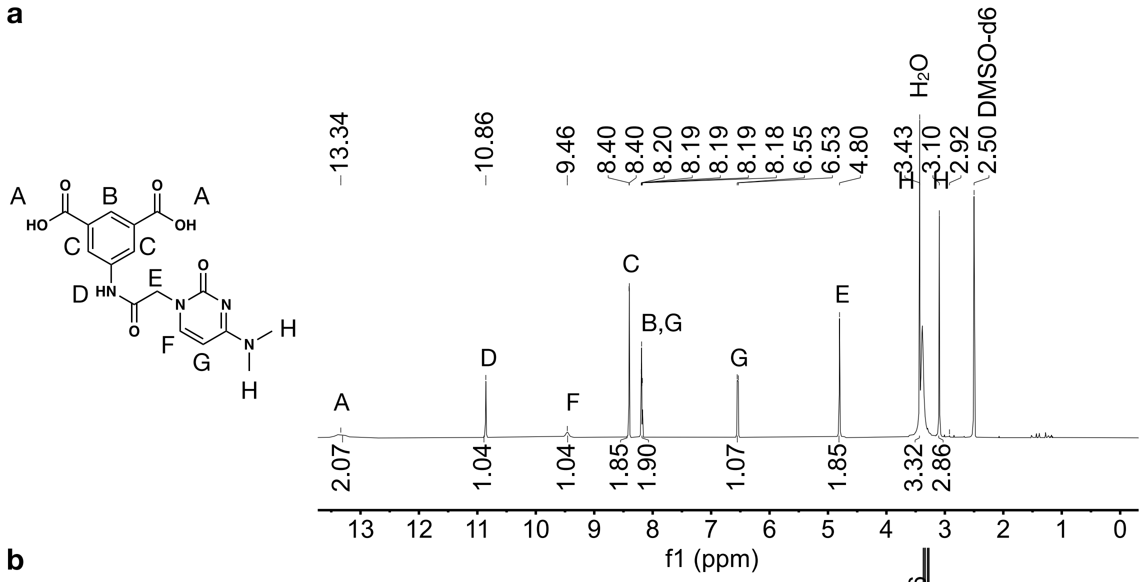

b

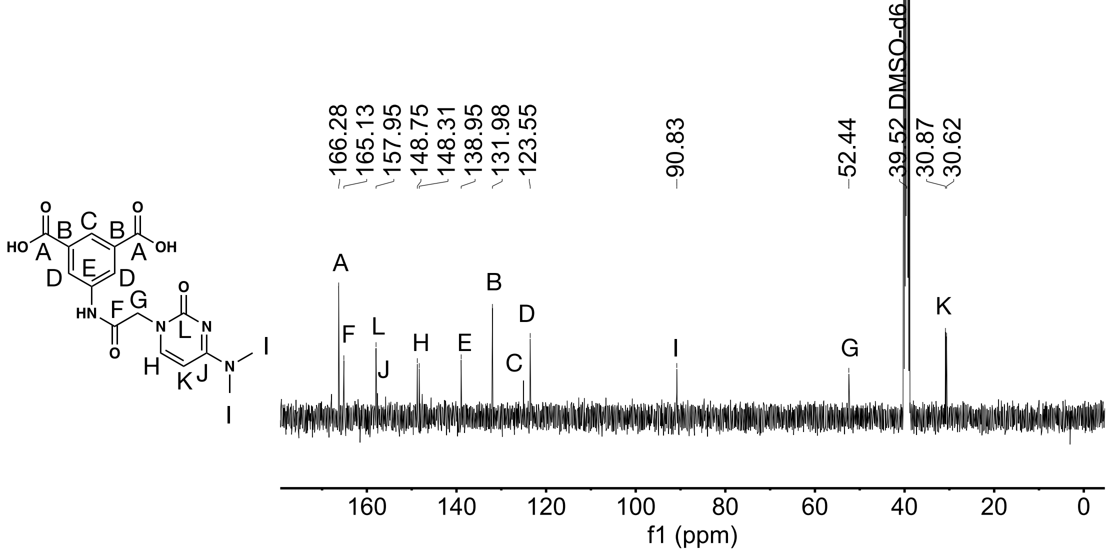

***Figure S16**. **(a)** ^{1}H-NMR and **(b)** ^{13}C-NMR spectra of **Me-C**.*

a

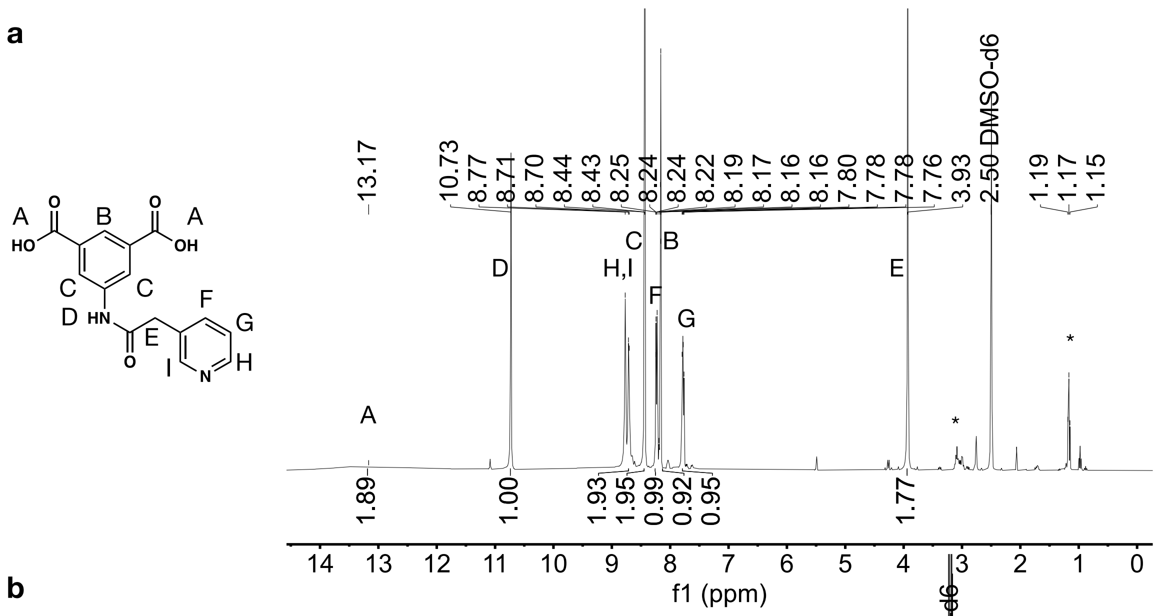

b

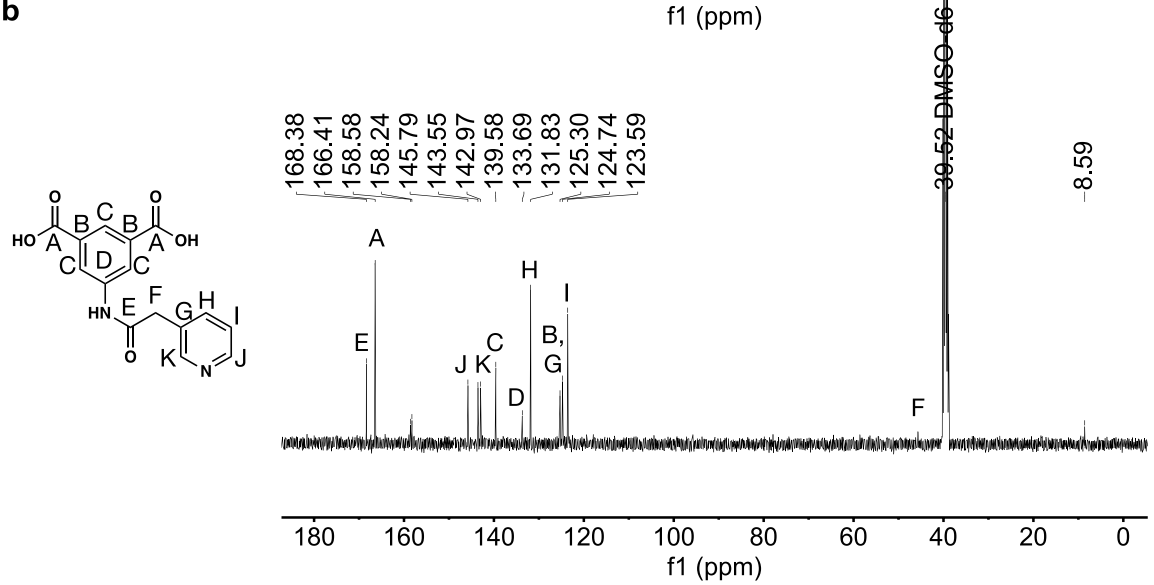

***Figure S17**. **(a)** ^{1}H-NMR and **(b)** ^{13}C-NMR spectra of **3-pyridyl-IPA**. * from acetate.*

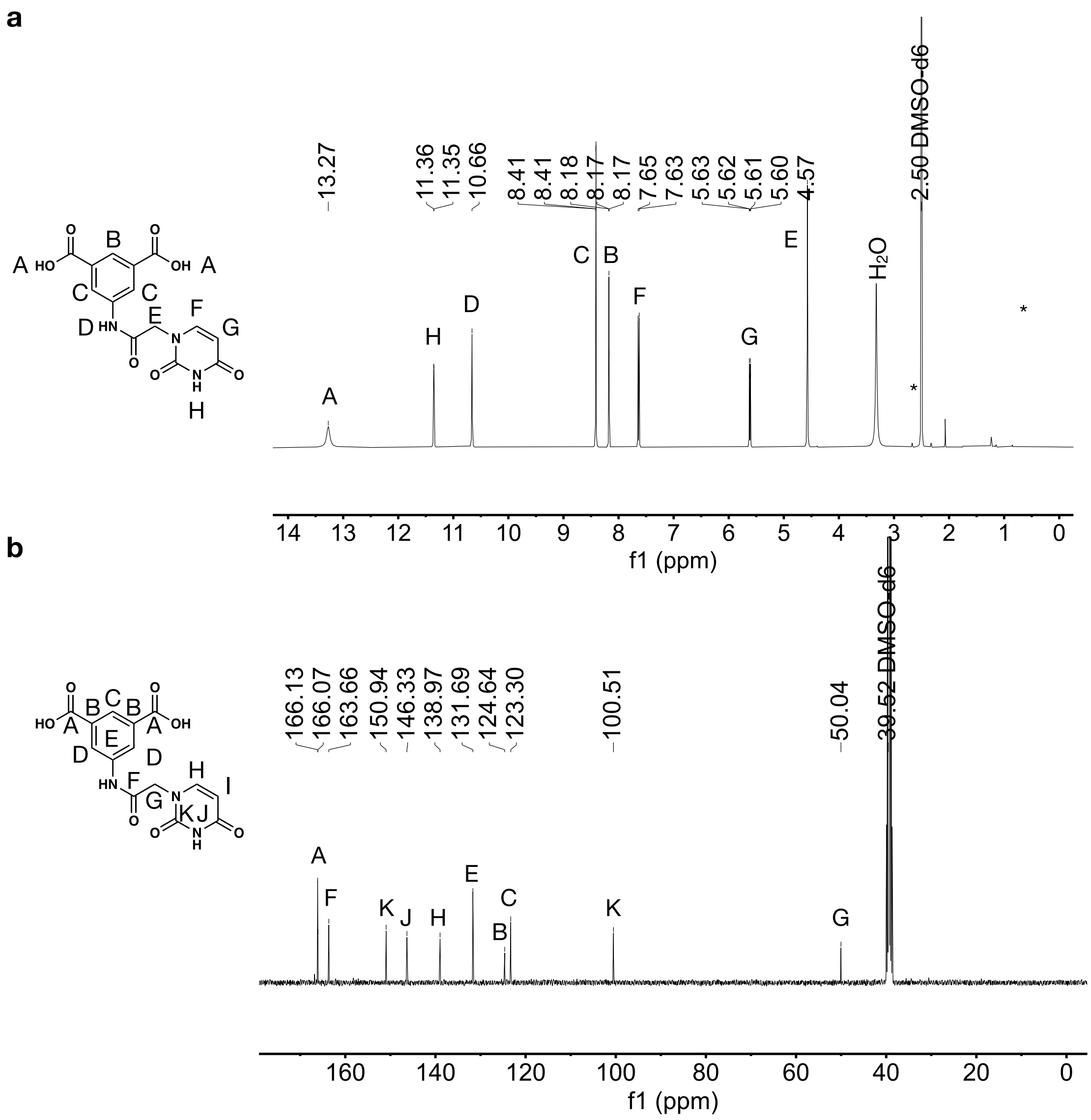

***Figure S18**. **(a)** ^{1}H-NMR and **(b)** ^{13}C-NMR spectra of **U**.*

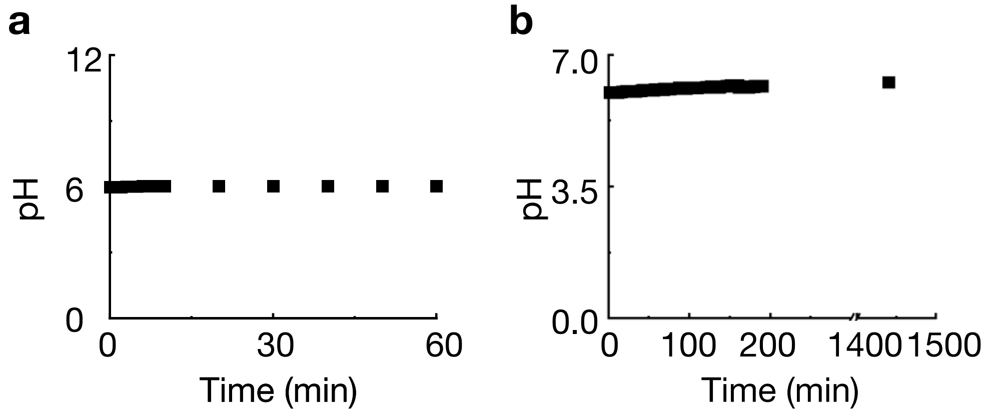

Figure S19. *pH profile of batch-fueled experiment with* ***(a) T*** *or* ***(b) C****.*

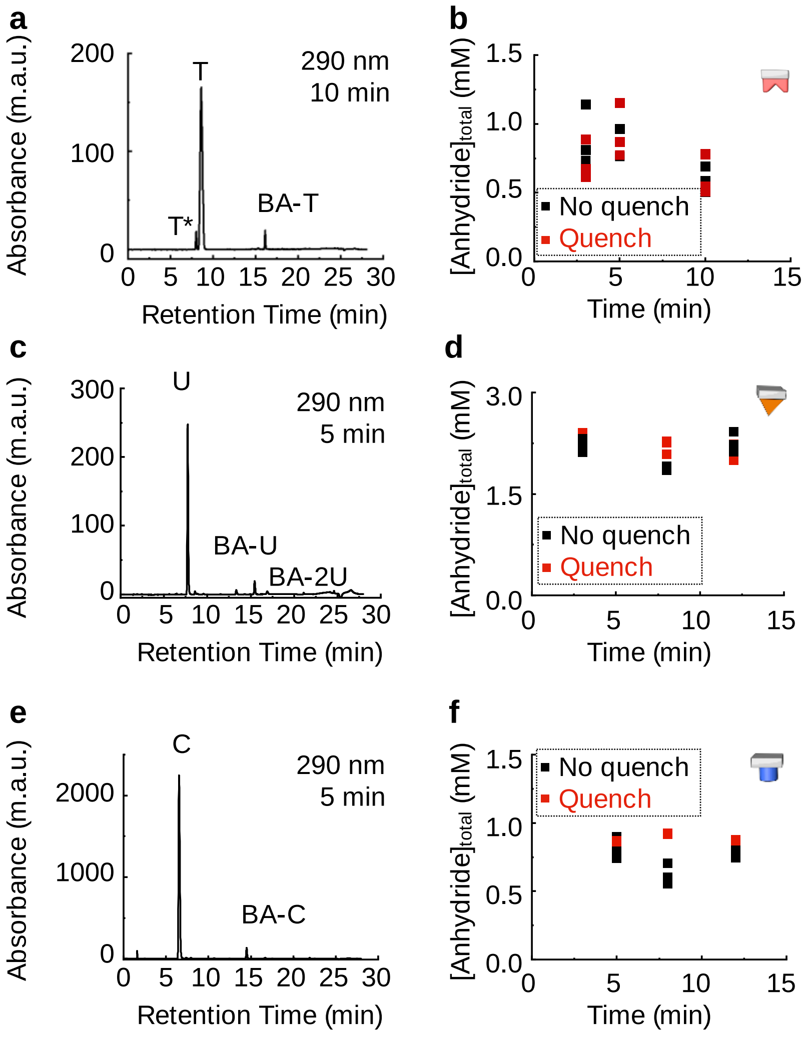

Figure S20. (a, c) *HPLC chromatogram after quenching the oligomer library made from* ***a) T****,* ***c) U****, and* ***e) C*** *with benzylamine 10 min* ***(a)****, 5 min* ***(c****,* ***e)****, or 3 min* ***(e)*** *after starting the reaction cycle adding 10 mM (****T****) or 25 mM (****U****,* ***C****) of EDC.* ***(b, d, f)*** *Concentration profile of the total anhydride as a function of time when fueling* ***T (b)****,* ***U (d)****, and* ***C (f)*** *with and without applying the benzylamine quench.*

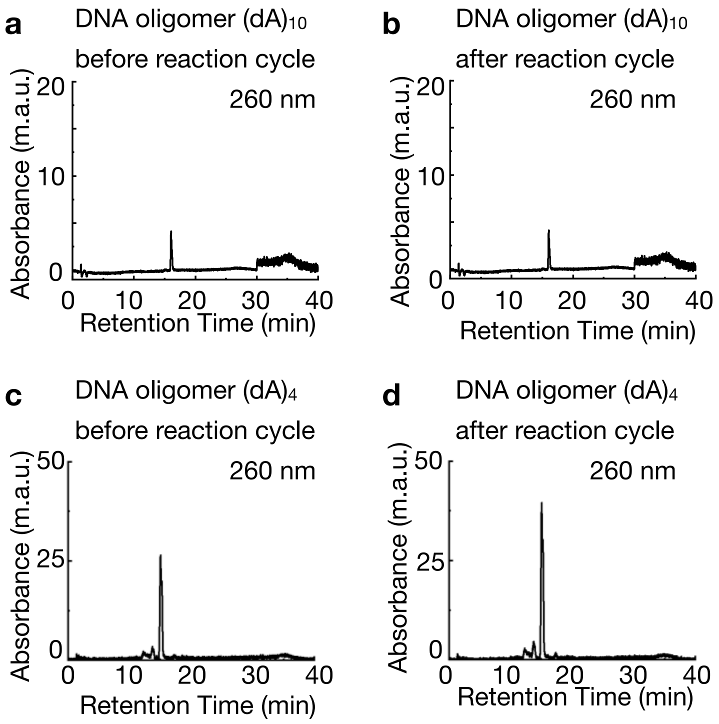

Figure S21. *HPLC trace of the repurified template* $(dA)_{10}$ ***(a, b)*** *and* $(dA)_4$ ***(c, d)*** *after the reaction cycle.*

References

1 Roberts, J. M. *et al.* Urea Metal–Organic Frameworks as Effective and Size-Selective Hydrogen-Bond Catalysts. *J. Am. Chem. Soc.* **134**, 3334-3337, doi:10.1021/ja2108118 (2012).

2 Wojciechowski, F. & Hudson, R. H. E. A Convenient Route to N-[2-(Fmoc)aminoethyl]glycine Esters and PNA Oligomerization Using a Bis-N-Boc Nucleobase Protecting Group Strategy. *J. Org. Chem.* **73**, 3807-3816, doi:10.1021/jo800195j (2008).

3 Schnitter, F. & Boekhoven, J. A Method to Quench Carbodiimide-Fueled Self-Assembly. *ChemSystemsChem* **3**, e2000037, doi:10.1002/syst.202000037 (2021).

4 Kriebisch, C. M. E., Bergmann, A. M. & Boekhoven, J. Fuel-Driven Dynamic Combinatorial Libraries. *J. Am. Chem. Soc.* **143**, 7719-7725, doi:10.1021/jacs.1c01616 (2021).

5 M.R. Green, J. S. Precipitation of DNA with Ethanol. *Cold Spring Harb. Protoc.*, doi:10.1101/pdb.prot093377 (2016).

6 Iwaura, R. *et al.* Oligonucleotide-Templated Self-Assembly of Nucleotide Bolaamphiphiles: DNA-Like Nanofibers Edged by a Double-Helical Arrangement of A–T Base Pairs. *Angew. Chem. Int. Ed.* **42**, 1009-1012, doi:10.1002/anie.200390257 (2003).

7 Simeth, N. A. *et al.* Photoswitchable architecture transformation of a DNA-hybrid assembly at the microscopic and macroscopic scale. *Chem. Sci.*, doi:10.1039/d1sc06490h (2022).

8 Spoelstra, W. K., van der Sluis, E. O., Dogterom, M. & Reese, L. Nonspherical Coacervate Shapes in an Enzyme-Driven Active System. *Langmuir* **36**, 1956-1964, doi:10.1021/acs.langmuir.9b02719 (2020).

9 Johanson, G. in *Comprehensive Toxicology (2nd Edition)*, (ed Charlene A. McQueen), 153-177 (Elsevier, 2010).

10 Sosson, M. & Richert, C. Enzyme-free genetic copying of DNA and RNA sequences. *Beilstein J. Org. Chem.* **14**, 603-617, doi:10.3762/bjoc.14.47 (2018).

11 Sosson, M., Pfeffer, D. & Richert, C. Enzyme-free ligation of dimers and trimers to RNA primers. *Nucleic Acids Res.* **47**, 3836-3845, doi:10.1093/nar/gkz160 (2019).

12 Todisco, M. & Szostak, J. W. Hybridization kinetics of out-of-equilibrium mixtures of short RNA oligonucleotides. *Nucleic Acids Res.* **50**, 9647-9662, doi:10.1093/nar/gkac784 (2022).

13 Hertel, S. *et al.* The stability and number of nucleating interactions determine DNA hybridization rates in the absence of secondary structure. *Nucleic Acids Res.* **50**, 7829-7841, doi:10.1093/nar/gkac590 (2022).

14 Gao, Y., Wolf, L. K. & Georgiadis, R. M. Secondary structure effects on DNA hybridization kinetics: a solution versus surface comparison. *Nucleic Acids Res.* **34**, 3370-3377, doi:10.1093/nar/gkl422 (2006).
