# Supplementary figures and images for: Template-based copying in chemically fuelled dynamic combinatorial libraries
Source: Nat Chem. 2024 Jul 16;16(8):1240–9. doi: 10.1038/s41557-024-01570-5 (PMC11321992; doi:10.1038/s41557-024-01570-5)

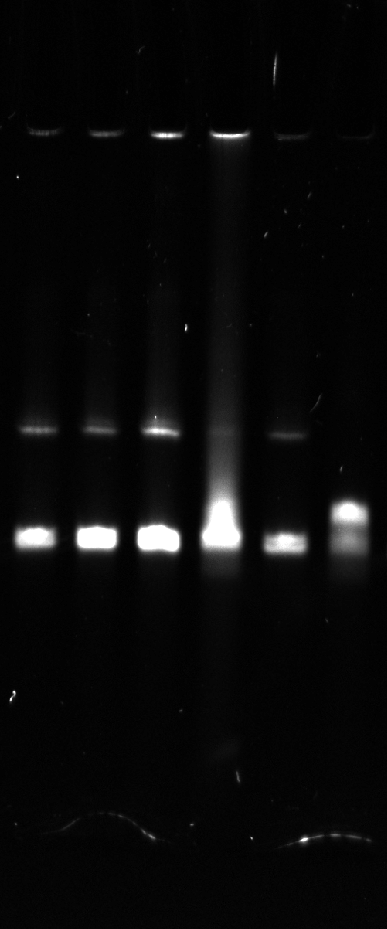

Supplement: Supplementary file 3 — Source data and unprocessed gel. [file 41557_2024_1570_MOESM3_ESM.zip › Fig3_SD/Fig3h_uncropped.tiff]

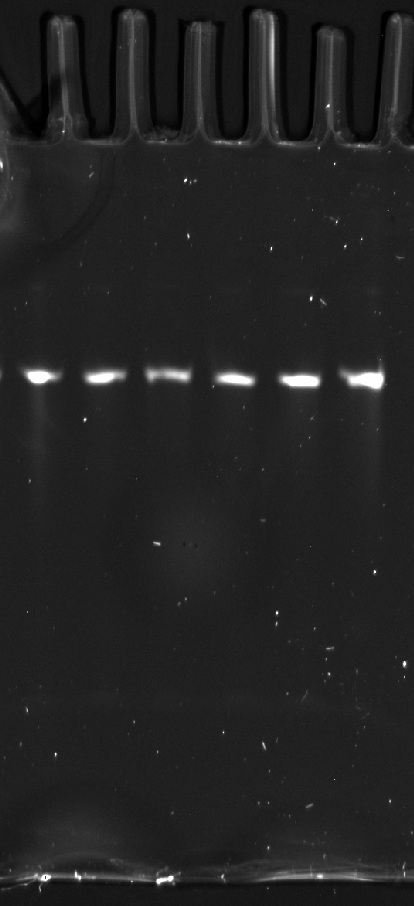

Supplement: Supplementary file 8 — Source data and unprocessed gel. [file 41557_2024_1570_MOESM8_ESM.zip › ExtDataFig3_SD/ExtFig3a_uncropped.tiff]
